# Mitochondria- and NOX4-dependent antioxidant defense mitigates progression to nonalcoholic steatohepatitis in obesity

**DOI:** 10.1172/JCI162533

**Published:** 2024-02-01

**Authors:** Spencer Greatorex, Supreet Kaur, Chrysovalantou E. Xirouchaki, Pei K. Goh, Florian Wiede, Amanda J. Genders, Melanie Tran, YaoYao Jia, Arthe Raajendiran, Wendy A. Brown, Catriona A. McLean, Junichi Sadoshima, Matthew J. Watt, Tony Tiganis

**Affiliations:** 1 Monash Biomedicine Discovery Institute,; 2 Department of Biochemistry and Molecular Biology,; 3 Department of Surgery, Alfred Hospital, Monash University, Melbourne, Victoria, Australia.; 4 Anatomical Pathology, Alfred Hospital, Prahran, Victoria, Australia.; 5 Department of Cell Biology and Molecular Medicine, Cardiovascular Research Institute, Rutgers New Jersey Medical School, Newark, New Jersey, USA.; 6 Department of Anatomy and Physiology, University of Melbourne, Melbourne, Victoria, Australia.

**Keywords:** Hepatology, Metabolism, Diabetes, Glucose metabolism, Obesity

## Abstract

Nonalcoholic fatty liver disease (NAFLD) is prevalent in the majority of individuals with obesity, but in a subset of these individuals, it progresses to nonalcoholic steatohepatitis (0NASH) and fibrosis. The mechanisms that prevent NASH and fibrosis in the majority of patients with NAFLD remain unclear. Here, we report that NAD(P)H oxidase 4 (NOX4) and nuclear factor erythroid 2–related factor 2 (NFE2L2) were elevated in hepatocytes early in disease progression to prevent NASH and fibrosis. Mitochondria-derived ROS activated NFE2L2 to induce the expression of NOX4, which in turn generated H_2_O_2_ to exacerbate the NFE2L2 antioxidant defense response. The deletion or inhibition of NOX4 in hepatocytes decreased ROS and attenuated antioxidant defense to promote mitochondrial oxidative stress, damage proteins and lipids, diminish insulin signaling, and promote cell death upon oxidant challenge. Hepatocyte NOX4 deletion in high-fat diet–fed obese mice, which otherwise develop steatosis, but not NASH, resulted in hepatic oxidative damage, inflammation, and T cell recruitment to drive NASH and fibrosis, whereas NOX4 overexpression tempered the development of NASH and fibrosis in mice fed a NASH-promoting diet. Thus, mitochondria- and NOX4-derived ROS function in concert to drive a NFE2L2 antioxidant defense response to attenuate oxidative liver damage and progression to NASH and fibrosis in obesity.

## Abstract 2

<p>Mitochondrial- and NOX4-derived ROS drive NFE2L2 antioxidant defense to attenuate the progression to non-alcoholic steatohepatitis and fibrosis in obesity.</p>

## Graphical abstract

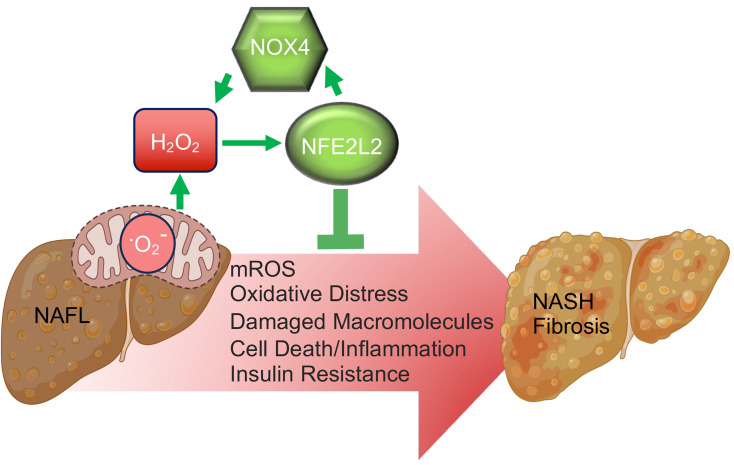

## Introduction

Nonalcoholic fatty liver disease (NAFLD) has reached epidemic proportions, affecting 20%–30% of the world’s population (1, 2). Although there are strong genetic determinants for disease onset and progression, the prevalence of NAFLD can be attributed predominantly to the entrenched and growing obesity and diabetes epidemics (1–3). NAFLD encompasses disorders ranging from simple steatosis, or nonalcoholic fatty liver (NAFL), to nonalcoholic steatohepatitis (NASH), which is evident in 20%–30% of patients with NAFLD and characterized by chronic lipid accumulation, liver damage, and lobular/portal inflammation involving the recruitment and activation of immune cells, especially T cells. The liver damage and inflammation elicit reparative processes that result in fibrosis (1). Ultimately, persistent reparative responses can lead to severe fibrosis or cirrhosis and end-stage liver disease, or even hepatocellular carcinoma (HCC) (1, 4). NASH is currently the second leading reason for liver transplantation, the fastest-growing cause of HCC, and a major contributor to cardiovascular disease (1, 4, 5).

It is well established that the severity of steatosis can predict progression to NASH, as well as the risk for cirrhosis (1, 6). Genetic polymorphisms in encodes patatin-like phospholipase domain–containing protein 3 (*PNPLA3*) that increase the risk for steatosis also increase the risk for NASH (1, 6). Steatosis occurs as a consequence of increased lipogenesis, increased uptake of dietary fatty acids and carbohydrates, and adipose tissue insulin resistance and the flux of free fatty acids to the liver (1, 6). However, the accumulation of lipids alone is not sufficient to drive progression to NASH and fibrosis (NASH and fibrosis). Indeed, the overexpression of diacylglycerol acyltransferase 2 (DGAT2) in mice or the deletion of adipose triglyceride lipase (ATGL) in hepatocytes increase steatosis without promoting inflammation, a key feature of NASH (7, 8). Chronic lipid accumulation in NAFL can increase mitochondrial β-oxidation and respiration (6, 9–11). The increased fatty acid oxidation and the excess supply of reduced substrates to the electron transport chain (ETC) are thought to result in increased electron leakage to generate superoxide (O_2_•^–^) (9, 12), but mitochondrial abnormalities or functional changes in the ETC may also contribute to O_2_•^–^ generation (9, 10, 13–15). Genetic and pharmacological studies in rodents point toward mitochondrial oxidative stress not only promoting insulin resistance, a key driver of NAFLD (15–21), but also NASH and fibrosis (9, 20, 21). Indeed, a common polymorphism in the gene encoding mitochondria-targeted superoxide dismutase 2 (SOD2), which converts O_2_•^–^ into hydrogen peroxide (H_2_O_2_), diminishes SOD2 function and is associated with more advanced fibrosis in NASH (22). However, several other processes including inflammation, ER stress, and increased NOX expression might also contribute to ROS generation (9, 23–26). In particular, the expression of NOX4, which can generate both O_2_•^–^ and H_2_O_2_ (27), is increased in the livers of patients with NAFLD, and its deletion in hepatocytes has been shown to attenuate NASH and fibrosis in mice fed a NASH-promoting diet (24). O_2_•^–^ can react with nitric oxide to generate toxic peroxynitrite, or promote the conversion of H_2_O_2_ into highly reactive hydroxyl radicals to damage proteins, lipids, and DNA (9). Increased ROS and the oxidative damage of macromolecules have been noted in the livers of rodents and patients with NAFLD (10, 28–31), and such oxidative damage can promote cell death and elicit reparative and inflammatory responses that result in fibrosis (9). Moreover, oxidative stress in NAFLD can result in the oxidative inactivation of protein tyrosine phosphatases in hepatocytes to promote tyrosine phosphorylation–dependent signaling, including STAT1 signaling, to facilitate T cell recruitment, inflammation, and the progression to NASH and fibrosis (32).

Given the potential for lipid accumulation to drive oxidative stress, it is perplexing why the majority of patients with NAFLD don’t progress to develop NASH and fibrosis. One possibility is that adaptive mechanisms temper the oxidative damage that may otherwise occur along the NAFLD continuum. One such mechanism may involve nuclear factor erythroid 2–related factor 2 (NFE2L2), a transcription factor that binds to antioxidant response elements (AREs) in the promoter regions of antioxidant and cytoprotective genes (33). Although NFE2L2 is normally targeted for degradation by the kelch-like ECH-associated protein 1 (KEAP1)–cullin-3 E3 ligase complex, ROS oxidize Cys residues on KEAP1 to facilitate the release and translocation of NFE2L2 to the nucleus to drive the expression of more than 200 endogenous antioxidant and xenobiotic detoxifying enzymes (33). Interestingly, the *NFE2L2* gene contains AREs within its promoter region so that the NFE2L2 protein can drive its own transcription to amplify the effects of NFE2L2 (34). Genetic studies in mice have yielded conflicting evidence as to the roles of NFE2L2 and KEAP1 in NAFLD. (35–40). We report that hepatic NFE2L2 target genes were elevated in NAFLD and that this served to attenuate the progression to NASH and fibrosis. We show that the induction and activation of NFE2L2 was reliant on ROS generated by both mitochondria and NOX4 and that *Nox4* deletion in hepatocytes was sufficient to abrogate the antioxidant defense response to promote oxidative lipid and protein damage, cell death, inflammation and the transition to NASH and fibrosis in obesity.

## Results

### NFE2L2 redox signatures in NAFLD.

**Figure 1:**
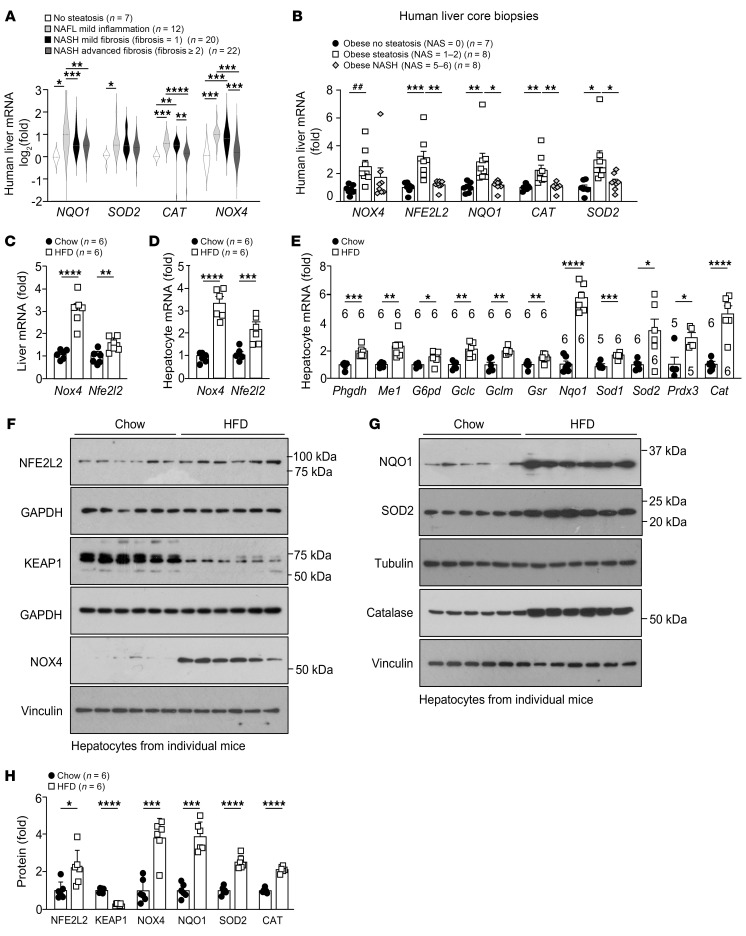
NOX4 and NFE2L2 antioxidant defense gene expression in NAFLD. (**A**) RNA-Seq analysis (GSE130970) of livers from patients without steatosis (*n* = 7), patients with NAFL (*n* = 13; NAS = 1–4, fibrosis score = 0), patients with NASH with mild fibrosis (*n* = 20; NAS = 3–6, fibrosis score = 1), and patients with NASH with advanced fibrosis (*n* = 22; NAS = 3–6, fibrosis score ≥2). (**B**) qPCR analysis of liver core biopsies from patients with obesity without steatosis (*n* = 7; NAS = 0), with NAFL (*n* = 8; NAS = 1–2), or with NASH and fibrosis (*n* = 8; NAS = 5–6, fibrosis score = 1–2). (**C**) C57BL/6 mice were fed a chow diet or a HFD for 12 weeks, and livers were processed for qPCR. (**D**–**H**) C57BL/6 mice were fed a chow diet or a HFD for 8–10 weeks, and hepatocytes were isolated and processed for (**D** and **E**) qPCR or (**F** and **G**) immunoblotting. (**H**) Quantification of protein levels for **F** and **G**. Representative and quantified results are shown as the mean ± SEM for the indicated number of mice. *P < 0.05, **P < 0.01, ***P < 0.001, and ****P < 0.0001, by 1-way ANOVA (**A** and **B**) or Student’s *t* test (**C**–**E** and **H**); ^##^*P* <0.05, by Student’s *t* test (**B**).

To assess redox balance in NAFLD, we first took advantage of a publicly available RNA-Seq data set (41) to assess the expression of redox genes in liver biopsies from lean or overweight individuals (body mass index [BMI] = 25.9 ± 5.8) without steatosis (NAFLD activity score [NAS] = 0–1) and from overweight or obese individuals (BMI = 33.9 ± 5.8) with NAFL and mild lobular inflammation (NAS = 1–4), NASH (NAS = 1–6) with mild fibrosis (fibrosis score = 1), or NASH (NAS = 2–6) with significant or advanced fibrosis (fibrosis score ≥2) (Supplemental Table 1). We found that the expression of genes targeted by NFE2L2 was increased in NAFL and NASH livers with mild fibrosis, but was decreased in NASH livers with more advanced fibrosis (Figure 1A). In particular, *NQO1*, which encodes the superoxide scavenger NAD(P)H dehydrogenase (quinone 1), *SOD2*, which converts O_2_•^–^ into H_2_O_2_ and catalase (*CAT*), which eliminates H_2_O_2_, were increased in individuals with NAFL or NASH with mild fibrosis, but tended to decline in individuals with NASH with significant/advanced fibrosis. Interestingly, the expression of *NOX4*, which has two NFE2L2-binding AREs in its promoter (42), also increased in patients with NAFL or NASH with mild fibrosis but declined in those with NASH with significant/advanced fibrosis (Figure 1A). Consistent with this, *NFE2L2*, *NQO1*, *SOD2*, *CAT*, and *NOX4* mRNA levels, as assessed by quantitative real-time PCR (qPCR) (Figure 1B), were also increased in liver core biopsies from patients with obesity (BMI = 36–61) with NAFL (NAS = 1–2), when compared with those from patients with obesity (BMI = 36–61) without steatosis (NAS = 0) (32). Moreover, except for *NOX4* expression, which trended lower, *NFE2L2*, *NQO1*, *SOD2*, and *CAT* gene expression declined in patients with obesity (BMI = 47–74) with NASH and fibrosis (NAS = 5–6; fibrosis score = 1–2) to levels evident in patients with obesity with nonsteatotic livers (Figure 1B). Therefore, NAFL, but not obesity per se, was accompanied by the increased expression of NFE2L2 targets genes, including *NOX4*, which encodes a ROS-producing enzyme, and *NQO1*, *SOD2*, and *CAT*, which encode key antioxidant defense enzymes, but these declined with more advanced disease and fibrosis.

As NOX4 and NFE2L2 are expressed in both hepatocytes and nonparenchymal cells, we next explored whether their induction in NAFL may be hepatocyte intrinsic. We assessed the expression of redox genes in the livers and hepatocytes of chow-fed lean versus high-fat diet–fed (HFD-fed) obese mice that developed liver steatosis but did not progress to NASH and fibrosis. Hepatocytes from HFD-fed mice were steatotic, as reflected by the accumulation of lipids and the expression of lipogenic genes (Supplemental Figure 1, A and B; supplemental material available online with this article; https://doi.org/10.1172/JCI162533DS1). As in humans, *Nfe2l2* and *Nox4* mRNA levels were induced in livers (Figure 1C) or hepatocytes (Figure 1D) from HFD-fed mice. The induction of *Nfe2l2* in hepatocytes from HFD-fed mice was accompanied by the increased expression of NFE2L2 target genes (Figure 1E) encoding enzymes involved in (a) NADPH production necessary for the reduction of oxidized glutathione (GSH) to reduced GSH, including phosphoglycerate dehydrogenase (*Phgdh*), malic enzyme 1 (*Me1*), and glucose-6-phosphate dehydrogenase (*G6pd*); (b) GSH production and regeneration, including glutamate-cysteine ligase (GCL) catalytic subunit (*Gclc*), GCL complex modifier subunit (*Gclm*) and glutathione reductase (*Gsr*); (c) quinone detoxification, including NQO1 (*Nqo1*); and (d) ROS detoxification, including SOD1 (*Sod1)* and SOD2 (*Sod2*), peroxiredoxin-3 (*Prdx3*), and *Cat*. Several of these genes were also increased in the livers of HFD-fed mice (Supplemental Figure 1C). The expression of *Cybb*, which encodes the O_2_•^–^ producing enzyme NOX2, was modestly increased, whereas the expression of *Ncf1* and *Rac1*, which encode the NOX2 regulatory subunits p47^phox^ and RAC1, was not altered (Supplemental Figure 1D). The induction of *Nfe2l2* and *Nox4* in hepatocytes from HFD-fed mice was accompanied by increased NFE2L2 and NOX4 protein levels, as well as increased NQO1, SOD2, and catalase (Figure 1, F–H), with no overt differences in NOX2, p47^phox^, or RAC1 protein levels (Supplemental Figure 1E). Moreover, in line with the increased NFE2L2 and NFE2L2 transcriptional targets, the abundance of KEAP1, which normally binds and targets NFE2L2 for degradation (33), was decreased (Figure 1F). Finally, in line with the induction of NOX4 being an outcome of increased NFE2L2-dependent transcription, we found that the NFE2L2 agonist isothiocyanate sulforaphane readily induced *Nox4* in hepatocytes (Supplemental Figure 1F). Thus, steatosis in obesity was accompanied by the induction of NOX4 and the NFE2L2 antioxidant defense response in hepatocytes.

### Lipids and mitochondrial ROS drive antioxidant defense in NAFLD.

**Figure 2:**
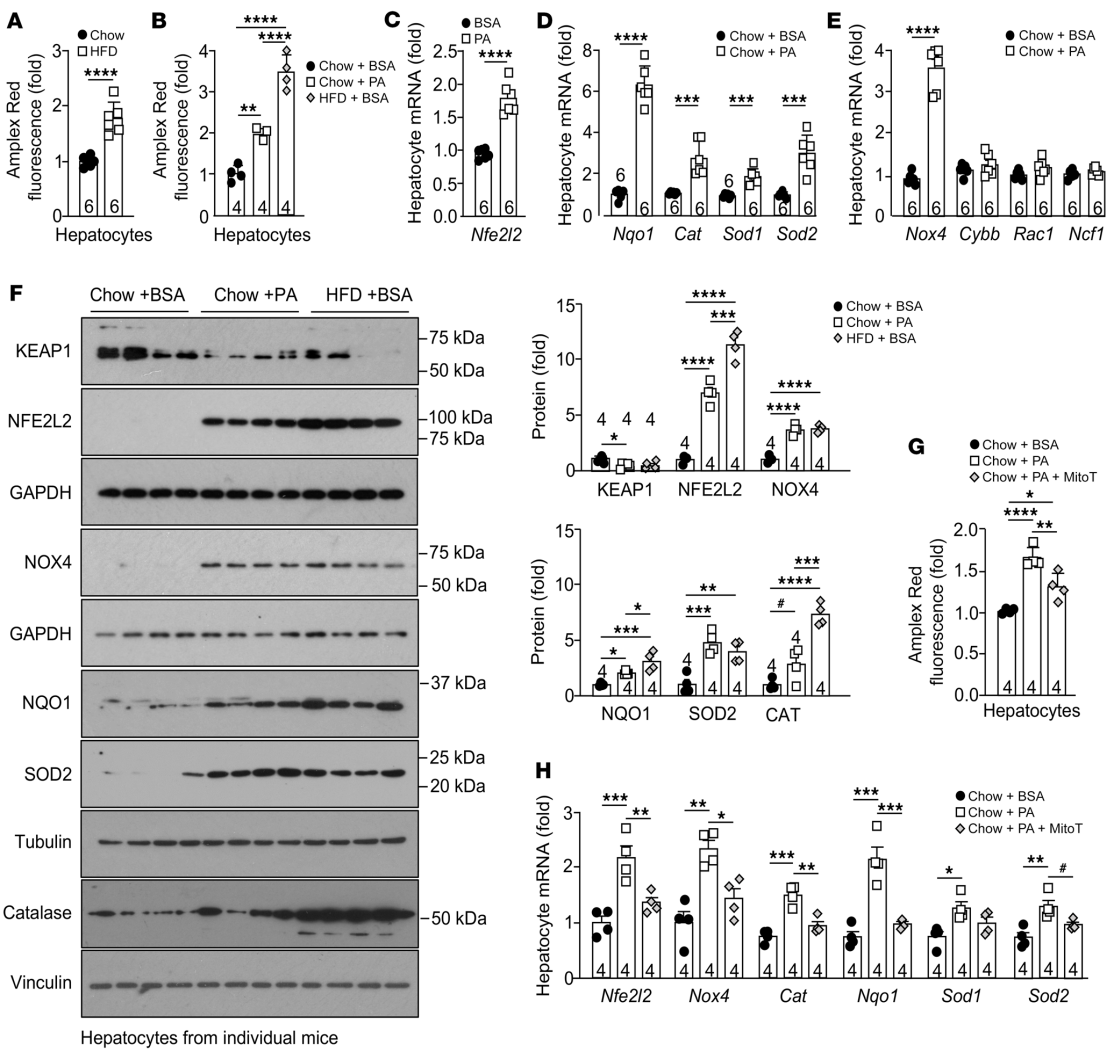
Lipids and mitochondrial ROS drive antioxidant defense. (**A** and **B**) C57BL/6 mice were fed a chow diet and/or a HFD for 8–10 weeks, and hepatocytes were isolated, cultured for 16 hours, and (**A**) processed for extracellular H_2_O_2_ measurement using Amplex Red; or (**B**) were treated with BSA-conjugated PA or BSA for 16 hours and processed for H_2_O_2_ measurement or (**C**–**E**) for qPCR; or (**F**) were treated with BSA or PA for 36 hours and processed for immunoblotting; or (**G** and **H**) were treated with BSA, PA, or PA plus 20 μM mitoTEMPOL (MitoT) for 16 hours and processed for qPCR. Representative and quantified results are shown as the mean ± SEM for the indicated number of mice. *P < 0.05, **P < 0.01, ***P < 0.001, and ****P < 0.0001, by Student’s *t* test (**A** and **C**–**E**), or 1-way ANOVA (**B** and **F**–**H**); ^#^*P* < 0.05, by Student’s *t* test.

Chronic hepatic lipid accumulation in NAFLD is accompanied by increased mitochondrial O2•^–^ and H_2_O_2_ generation (9, 43). Accordingly, we determined the extent to which hepatic lipid accumulation and heightened mitochondrial ROS might influence antioxidant defense. We first compared ROS generation by hepatocytes isolated from chow-fed versus HFD-fed mice with hepatocytes from chow-fed mice administered the saturated free fatty acid palmitate (PA). We monitored for the emission of H_2_O_2_ from live hepatocytes using the H_2_O_2_-selective probe Amplex Red (Amplex Red added to the culture medium). Hepatocytes from HFD-fed mice generated more ROS than did hepatocytes from chow-fed mice (Figure 2A). Also, hepatocytes from chow-fed mice treated with PA overnight generated more ROS than did vehicle-treated controls (Figure 2B and Supplemental Figure 2A). Importantly, the extent of PA-induced ROS generation approximated that seen in hepatocytes from HFD-fed mice (Figure 2B); after a more prolonged treatment, lipogenic gene expression was also increased (Supplemental Figure 2B), as noted in hepatocytes from HFD-fed mice. Next, we assessed whether PA-induced ROS might be sufficient to drive the antioxidant defense response. We found that PA treatment significantly increased the expression of *Nfe2l2* (Figure 2C) and NFE2L2 transcriptional targets, including *Nqo1*, *Sod1*, *Sod2*, *Cat*, and *Nox4* (Figure 2, D and E, and Supplemental Figure 2C); by contrast, mRNAs for NOX2 subunits were unaltered (Figure 2E). The PA-induced increase in *Nfe2l2* was accompanied by increased NFE2L2 and decreased KEAP1 protein levels (Figure 2F). Consistent with this, NOX4, NQO1, SOD2, and catalase protein levels were all increased (Figure 2F). Indeed, NFE2L2, KEAP1, NQO1, SOD1, catalase, and NOX4 levels in PA-treated hepatocytes from chow-fed mice approximated those seen in hepatocytes isolated from HFD-fed mice (Figure 2F). Finally, we assessed whether the PA-induced antioxidant defense response and *Nox4* expression might be ascribed to increased mitochondrial ROS generation. To this end, we examined whether the mitochondria-targeted O_2_•^–^ scavenger and antioxidant mitoTEMPOL could reduce ROS and thereby antioxidant defense and *Nox4* expression. We found that treatment with mitoTEMPOL not only attenuated the PA-induced increase in H_2_O_2_ (Figure 2G), but also the expression of the antioxidant defense genes *Nfe2l2*, *Nqo1*, *Sod1*, *Sod2*, and *Cat* as well as the expression of *Nox4* (Figure 2H). Therefore, the heightened antioxidant defense response and the induction of NOX4 in NAFLD might be linked to enhanced ROS production by mitochondria.

### NOX4 is essential for antioxidant defense in hepatocytes.

**Figure 3:**
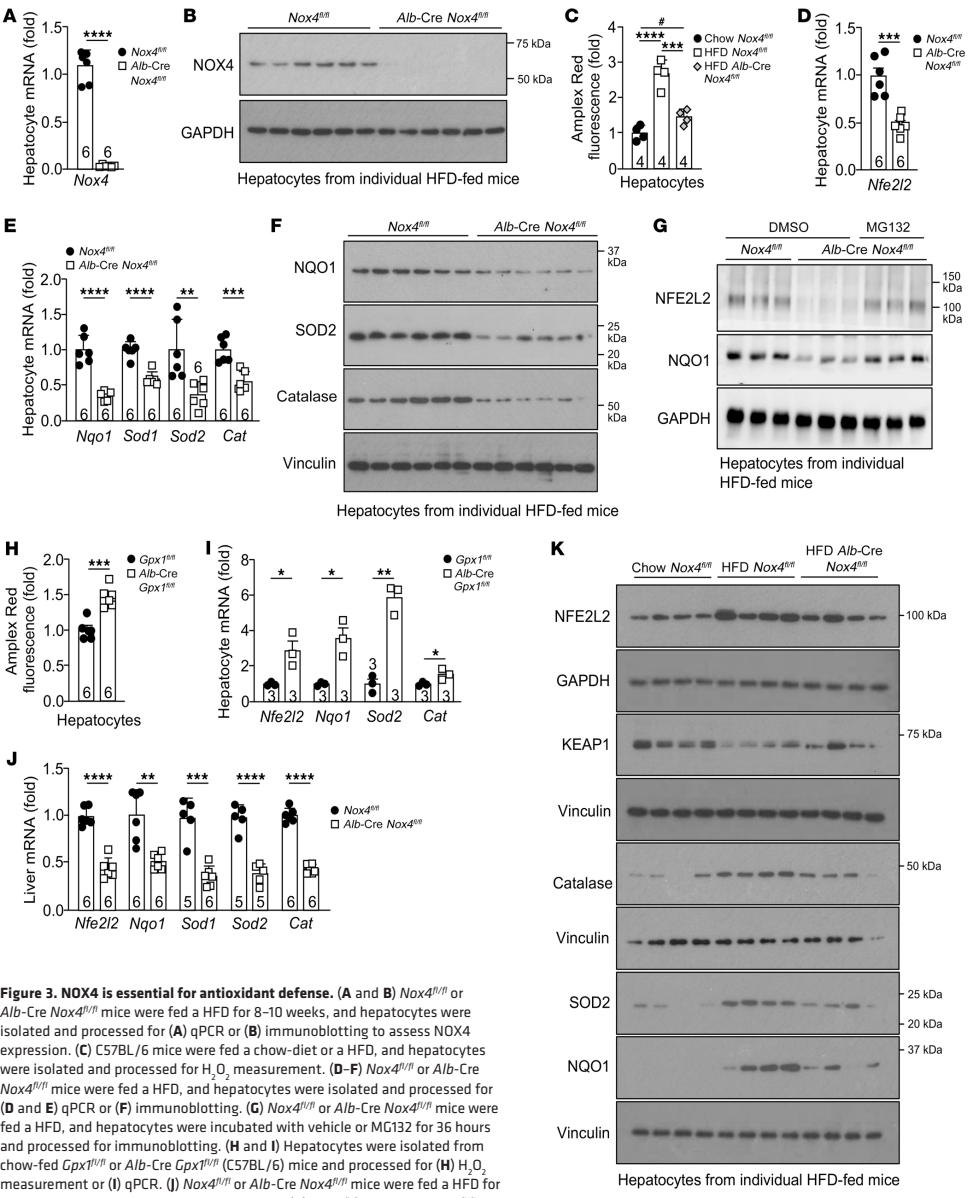
NOX4 is essential for antioxidant defense. (**A** and **B**) *Nox4^fl/fl^* or *Alb*-Cre *Nox4^fl/fl^* mice were fed a HFD for 8–10 weeks, and hepatocytes were isolated and processed for (**A**) qPCR or (**B**) immunoblotting to assess NOX4 expression. (**C**) C57BL/6 mice were fed a chow-diet or a HFD, and hepatocytes were isolated and processed for H_2_O_2_ measurement. (**D**–**F**) *Nox4^fl/fl^* or *Alb*-Cre *Nox4^fl/fl^* mice were fed a HFD, and hepatocytes were isolated and processed for (**D** and **E**) qPCR or (**F**) immunoblotting. (**G**) *Nox4^fl/fl^* or *Alb*-Cre *Nox4^fl/fl^* mice were fed a HFD, and hepatocytes were incubated with vehicle or MG132 for 36 hours and processed for immunoblotting. (**H** and **I**) Hepatocytes were isolated from chow-fed *Gpx1^fl/fl^* or *Alb*-Cre *Gpx1^fl/fl^* (C57BL/6) mice and processed for (**H**) H_2_O_2_ measurement or (**I**) qPCR. (**J**) *Nox4^fl/fl^* or *Alb*-Cre *Nox4^fl/fl^* mice were fed a HFD for 12 weeks, and livers were processed for qPCR. (**K**) *Nox4^fl/fl^* or *Alb*-Cre *Nox4^fl/fl^* mice were fed a chow diet or a HFD, and hepatocytes were isolated and processed for immunoblotting. Representative and quantified results are shown as the mean ± SEM for the indicated number of mice. *P < 0.05, **P < 0.01, ***P < 0.001, and ****P < 0.0001, by Student’s *t* test (**A**, **D**, **E**, and **H**–**J**) or 1-way ANOVA (**C**); ^#^*P* < 0.05, by Student’s *t* test (**C**).

**Figure 4:**
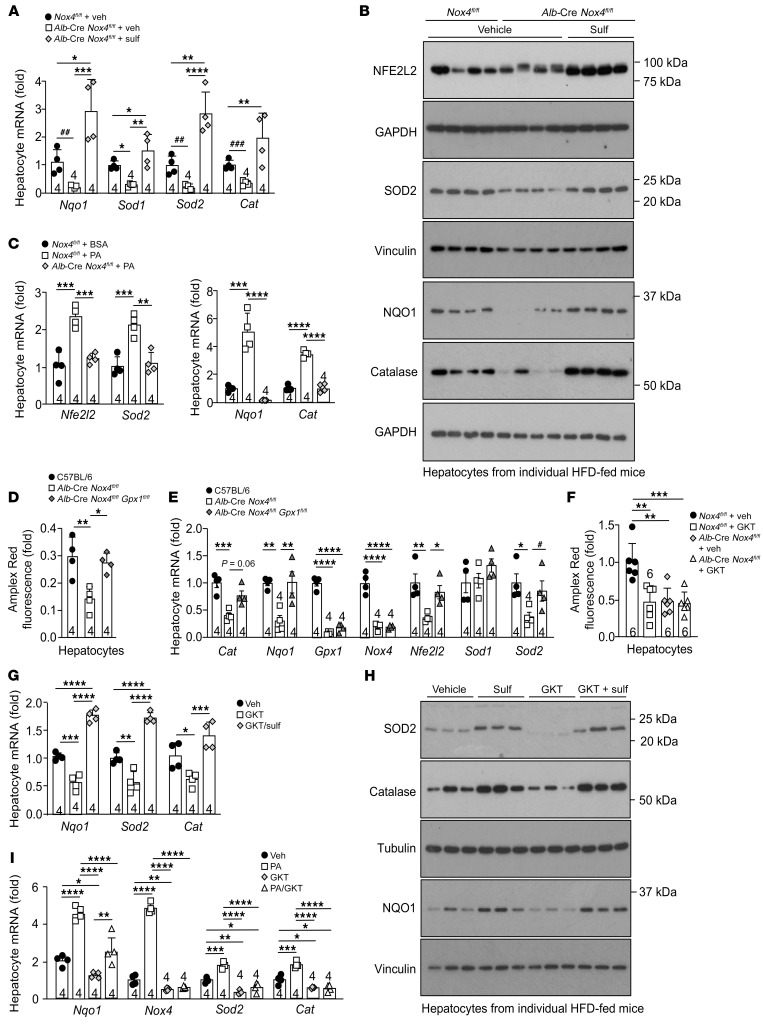
NOX4-derived H_2_O_2_ is essential for antioxidant defense. (**A** and **B**) *Nox4^fl/fl^* or *Alb*-Cre *Nox4^fl/fl^* mice were fed a HFD for 8–10 weeks, and hepatocytes were isolated and treated with vehicle (DMSO) or 1 μM sulforaphane (sulf) for (**A**) 16 hours and processed for qPCR or for (**B**) 36 hours and processed for immunoblotting. (**C**) Alternatively, hepatocytes were treated with BSA or PA for 16 hours and processed for qPCR. (**D** and **E**) Eight-week-old *Nox4^fl/fl^*, *Alb*-Cre *Nox4^fl/fl^*, and *Alb*-Cre *Nox4^fl/fl^* *Gpx1^fl/fl^* mice were fed a HFD for 8–10 weeks, and hepatocytes were isolated and processed for (**D**) H_2_O_2_ measurement and (**E**) qPCR. (**F**–**H**) *Nox4^fl/fl^* or *Alb*-Cre *Nox4^fl/fl^* mice were fed a HFD for 8–10 weeks, and isolated hepatocytes were treated with vehicle (DMSO), 40 μM GKT137831 (GKT), or GKT plus 1 μM sulforaphane for 16 hours and processed for (**F**) H_2_O_2_ measurement and (**G**) qPCR, or (**H**) for 36 hours and processed for immunoblotting. (**I**) Alternatively, hepatocytes were treated with vehicle, PA, GKT, or PA plus GKT for 16 hours and processed for qPCR. Representative and quantified results are shown as the mean ± SEM for the indicated number of mice. *P < 0.05, **P < 0.01, ***P < 0.001, and ****P < 0.0001, by 1-way ANOVA (**A**, **C**–**G**, and **I**); ^#^*P* < 0.05, ^##^*P* < 0.01, and ^###^*P* < 0.001, by Student’s *t* test (**A** and **E**).

Previously we have shown that NFE2L2 drives *Nox4* expression in muscle and, in turn, that NOX4-derived H_2_O_2_ enhances NFE2L2 antioxidant defense to attenuate muscle oxidative damage and insulin resistance (44). In this study, we have reaffirmed that the activation of NFE2L2 with sulforaphane was sufficient to induce *Nox4* expression in hepatocytes (Supplemental Figure 1F). We reasoned that the induction of NOX4 in hepatocytes in NAFLD might function as part of a feedback loop to exacerbate and/or sustain NFE2L2 antioxidant defense otherwise instigated by mitochondrial ROS. To test this, we isolated hepatocytes from control (*Nox4^fl/fl^*) (45) and hepatocyte-specific NOX4-deficient mice (*Alb*-Cre *Nox4^fl/fl^*) fed a HFD for 10 weeks to induce steatosis (Supplemental Figure 3A) and assessed H_2_O_2_ levels and the expression of NFE2L2 and its target genes. NOX4 was effectively ablated in hepatocytes (Figure 3, A and B). The deletion of *Nox4* attenuated the otherwise increased H_2_O_2_ emission by hepatocytes from HFD-fed mice (Figure 3C), but this still exceeded that from chow-fed *Nox4^fl/fl^* mice (Figure 3C), consistent with the contribution of both mitochondria and NOX4 to ROS generation. The deletion of *Nox4* also attenuated the expression of both *Nfe2l2* (Figure 3D) and NFE2L2 target genes, including *Nqo1*, *Sod1*, *Sod2*, and *Cat* (Figure 3E), as well as the corresponding NFE2L2, NQO1, SOD2, and catalase proteins (Figure 3, F and G). The reduced NFE2L2 and NQO1 levels could be rescued by incubating cells in the presence of the proteasome inhibitor MG132 (Figure 3G), consistent with NOX4-derived H_2_O_2_ otherwise preventing NFE2L2 degradation. Conversely, the deletion of *Gpx1*, encoding glutathione peroxidase 1 (GPX1), an enzyme that detoxifies H_2_O_2_, increased the emission of H_2_O_2_ by hepatocytes from HFD-fed mice (Figure 3H) and increased the expression of *Nfe2l2*, *Nqo1*, *Sod2*, and *Cat* (Figure 3I). Therefore, changes in H_2_O_2_ levels in hepatocytes from HFD-fed mice could elicit corresponding changes in antioxidant defense gene expression. The reduced expression of antioxidant defense genes and decreased NFE2L2 and NQO1 proteins associated with NOX4-deficiency in hepatocytes from HFD-fed mice were also evident in the livers of 12-week HFD-fed *Alb*-Cre *Nox4^fl/fl^* versus *Nox4^fl/fl^* mice (Figure 3J and Supplemental Figure 3B). The reduced antioxidant gene expression in hepatocytes from *Alb*-Cre *Nox4^fl/fl^* HFD-fed mice was accompanied by reduced NFE2L2 and increased KEAP1 protein levels, consistent with NOX4-derived ROS being required for the degradation of KEAP1 and the stabilization of NFE2L2 (Figure 3K and Supplemental Figure 3C). Importantly, as with ROS production, NFE2L2 levels in hepatocytes from HFD-fed *Alb*-Cre *Nox4^fl/fl^* mice tended to exceed those in hepatocytes from chow-fed *Nox4^fl/fl^* mice (Figure 3K and Supplemental Figure 3C), consistent with a contribution of both mitochondria- and NOX4-derived ROS to the antioxidant defense response. In line with this, SOD2, NQO1, and catalase levels were attenuated by NOX4 deficiency but still exceeded those in hepatocytes from chow-fed *Nox4^fl/fl^* mice (Figure 3K and Supplemental Figure 3C). Importantly, the reduced expression of antioxidant defense genes (Figure 4A) and NFE2L2, SOD2, NQO1, and catalase protein levels (Figure 4B and Supplemental Figure 3D) in hepatocytes from HFD-fed *Alb*-Cre *Nox4^fl/fl^* mice could be corrected by the administration of the NFE2L2 agonist sulforaphane, consistent with the abrogation of NFE2L2-dependent responses by NOX4 deficiency. Finally, we found that NOX4 deficiency in hepatocytes from chow-fed *Alb*-Cre *Nox4^fl/fl^* mice largely attenuated the PA-induced increase in antioxidant defense, as reflected by the expression of *Nfe2l2* and its target genes *Nqo1*, *Sod2*, and *Cat* (Figure 4C). Thus the induction of NOX4 in NAFLD, downstream of mitochondrial ROS, may be required for optimal NFE2L2 antioxidant defense responses.

To specifically assess whether NOX4-derived ROS in NAFLD contributes to antioxidant defense, we first examined the effect of *Gpx1* deletion. The compound deletion of *Gpx1* corrected the diminished H_2_O_2_ levels (Figure 4D) and the reduced antioxidant defense response (Figure 4E) in hepatocytes from HFD-fed *Alb*-Cre *Nox4^fl/fl^* mice. Next, to determine whether the effects of NOX4 deletion were attributed to decreased NOX4 activity, we took advantage of the NOX1/4 inhibitor GKT137831 (46). We treated hepatocytes from HFD-fed *Nox4^fl/fl^* and *Alb*-Cre *Nox4^fl/fl^* mice with vehicle or GKT137831 and measured ROS production and antioxidant defense. GKT137831 was just as efficient as NOX4 deletion in reducing H_2_O_2_ levels (Figure 4F). Since we could not detect *Nox1* in isolated hepatocytes by qPCR, we surmised that these effects were due to the inhibition of NOX4. Consistent with this, GKT137831 did not further reduce the emission of H_2_O_2_ by NOX4-deficient hepatocytes (Figure 4F). The inhibition of NOX4 and the reduction in ROS production were accompanied by reductions in the expression of NFE2L2 targets genes (Figure 4G) and corresponding proteins (Figure 4H). Importantly, the decreased antioxidant defense associated with the inhibition of NOX4 could be corrected by the administration of the NFE2L2 agonist sulforaphane (Figure 4, G and H). Furthermore, GKT137831 repressed the PA-induced expression of *Nfe2l2* target genes, including *Nqo1*, *Sod2*, and *Cat* (Figure 4I). Taken together, these results indicate that the induction of NOX4 and the accompanying increased ROS in hepatocytes were essential for the antioxidant defense response that was instigated by mitochondrial ROS in NAFLD.

### NOX4 deletion in hepatocytes promotes oxidative stress, insulin resistance, and cell death.

**Figure 5:**
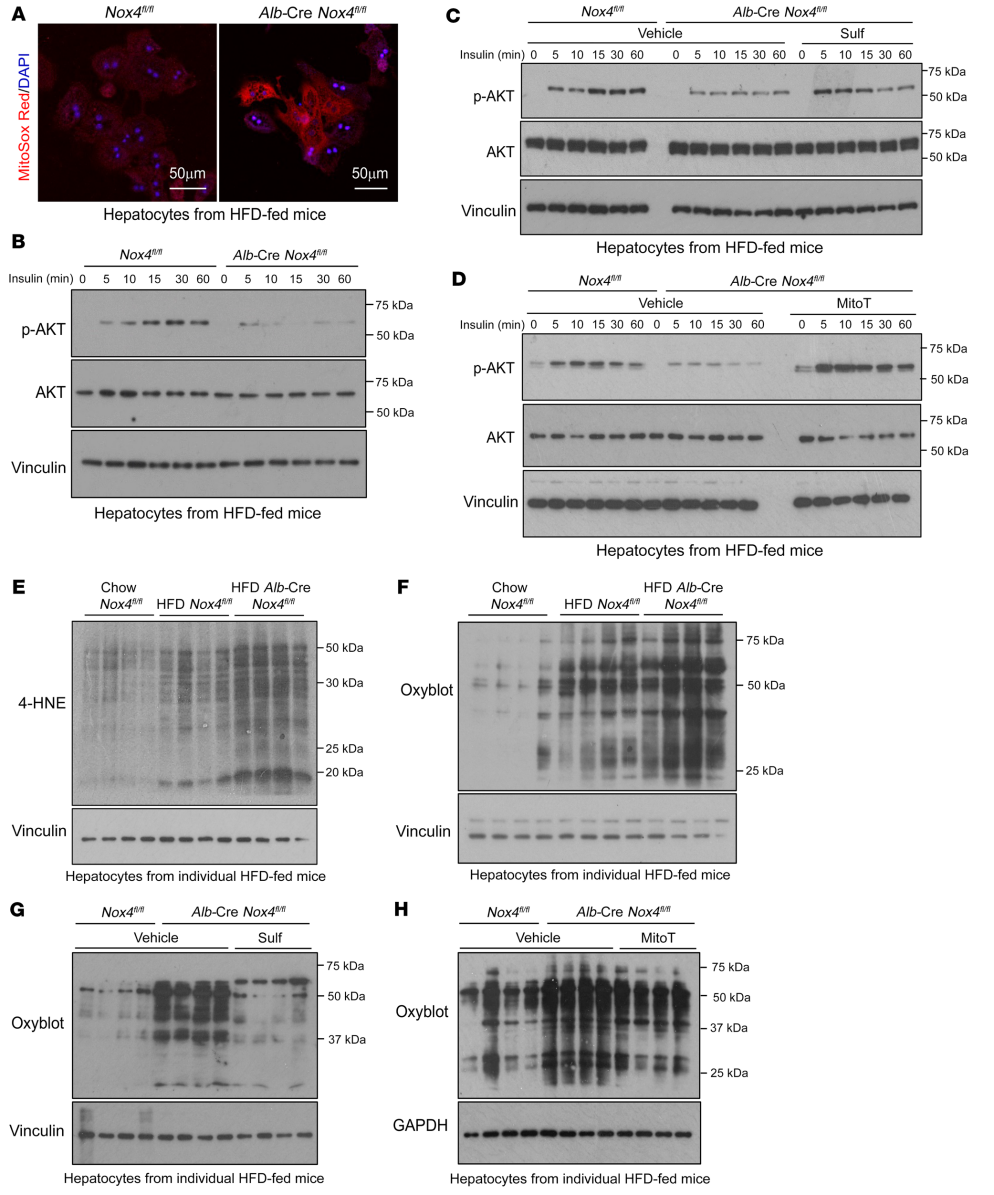
NOX4 deletion in hepatocytes promotes oxidative stress and insulin resistance. (**A** and **B**) *Nox4^fl/fl^* and *Alb*-Cre *Nox4^fl/fl^* mice were fed a HFD for 8–10 weeks, and isolated hepatocytes were either (**A**) stained with 1 μM MitoSOX Red and analyzed by confocal microscopy (counterstained with DAPI) or (**B**) serum starved and stimulated with 1 nM insulin and then processed for immunoblotting to measure p-AKT (Ser473) levels. Scale bars: 50 μm. (**C** and **D**) Alternatively, hepatocytes were treated with vehicle, 1 μM sulforaphane, or 20 μM MitoT for 16 hours and then serum starved. Next, they were stimulated with 1 nM insulin and processed for immunoblotting. (**E** and **F**) *Nox4^fl/fl^* and *Alb*-Cre *Nox4^fl/fl^*mice were fed a chow diet or a HFD for 8–10 weeks, and hepatocytes were isolated and processed for lipid peroxidation (4-HNE) or protein carbonylation (OxyBlot) analysis by immunoblotting. (**G** and **H**) *Nox4^fl/fl^* and *Alb*-Cre *Nox4^fl/fl^* mice were fed a HFD for 8–10 weeks, and isolated hepatocytes treated with vehicle, 1 μM sulforaphane, or 20 μM MitoT for 16 hours and processed for 4-HNE or OxyBlot immunoblotting. Results shown are representative of at least 3 independent experiments.

**Figure 6:**
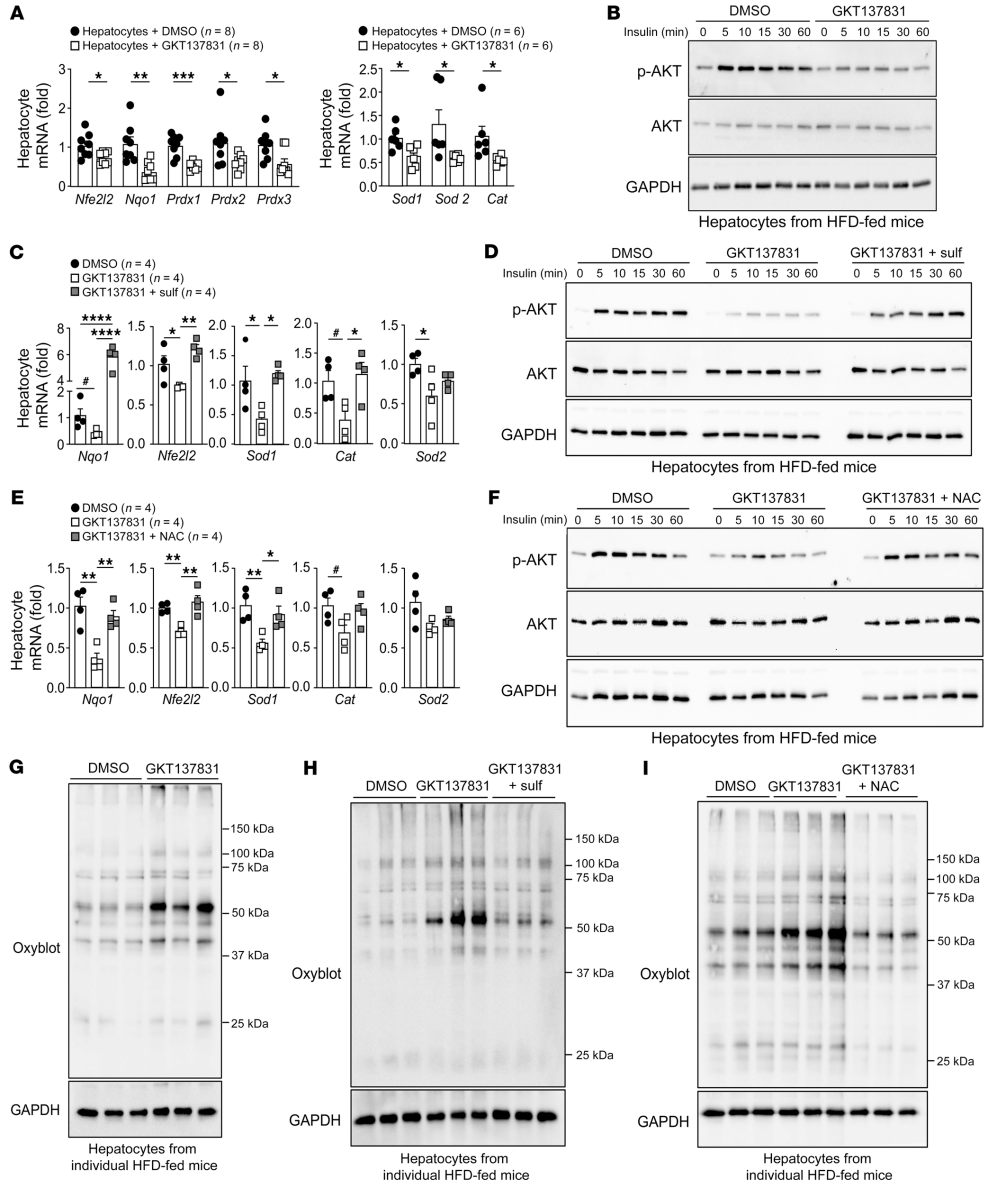
NOX4 inhibition diminishes antioxidant defense and promotes oxidative stress and insulin resistance. C57BL/6 mice were fed a HFD for 8–10 weeks, and hepatocytes were isolated and cultured for 16 hours at 5% O_2_. (**A** and **B**) Hepatocytes were treated with vehicle (DMSO) or 40 μM GKT137831 twice per day for 48 hours and processed for (**A**) qPCR or (**B**) were serum starved and stimulated with 2 nM insulin and processed for immunoblotting. (**C** and **D**) Hepatocytes were treated with vehicle, GKT137831 twice per day, or GKT137831 twice per day plus 1 μM sulforaphane once per day for 48 hours and processed for (**C**) qPCR or (**D**) immunoblotting. (**E**–**I**) Hepatocytes were treated with DMSO, GKT137831 twice per day, or GKT137831 twice per day plus NAC (1 mM) for 48 hours and then processed for (**E**) qPCR, or (**F**) stimulated with 2 nM insulin and processed for p-AKT immunoblotting, or (**G**–**I**) processed for OxyBlot immunoblotting. Representative and quantified results are shown as the mean ± SEM for the indicated number of mice. *P < 0.05, **P < 0.01, ***P < 0.001, and ****P < 0.0001, by Student’s *t* test (**A**) or 1-way ANOVA (**C** and **E**); ^#^*P* < 0.05, by Student’s *t* test (**C** and **E**).

Numerous studies have shown that oxidative stress and, in particular, increased mitochondrial ROS can contribute to the development of insulin resistance (16, 17, 19, 47). Indeed, the heterozygous deletion of *Sod2* and consequent increased mitochondrial O_2_•^–^ promote insulin resistance in chow-fed mice, whereas SOD2 overexpression, or the expression of mitochondria-targeted catalase attenuate systemic insulin resistance in HFD-fed mice (16, 17, 19). Therefore, we reasoned that ablating NOX4 and abrogating the resultant H_2_O_2_-dependent NFE2L2 antioxidant defense response, and, in particular, reducing SOD2, would promote mitochondrial oxidative stress to diminish insulin signaling and promote insulin resistance. To test this, we monitored by confocal microscopy for mitochondrial O_2_•^–^ levels using the mitochondrial O_2_•^–^ probe MitoSOX Red. The deletion of *Nox4* in hepatocytes increased mitochondrial O_2_•^–^ in NOX4-deficient hepatocytes isolated from HFD-fed mice (Figure 5A). The increase in mitochondrial O_2_•^–^ was accompanied by a reduction in insulin signaling, as monitored by phosphorylated AKT (Ser473) (p-AKT) in hepatocytes (Figure 5B and Supplemental Figure 4A). Importantly, treatment with the NFE2L2 agonist sulforaphane not only restored the decreased expression of antioxidant defense genes and the decreased abundance of NFE2L2, NQO1, SOD2, and catalase proteins (Figure 4B and Supplemental Figure 3D), but also partially restored insulin signaling in NOX4-deficient hepatocytes (Figure 5C and Supplemental Figure 4B). Moreover, treatment with the mitochondria-targeted O_2_•^–^ scavenger mitoTEMPOL restored insulin signaling in hepatocytes from HFD-fed *Alb*-Cre *Nox4^fl/fl^* mice (Figure 5D and Supplemental Figure 4C). Finally, the sustained inhibition of NOX4 with GKT137831 diminished the expression of antioxidant defense genes (Figure 6A) and insulin-induced p-AKT in hepatocytes from HFD-fed mice (Figure 6B), and both were prevented if hepatocytes were additionally cocultured with either sulforaphane (Figure 6, C and D) or the GSH precursor *N*-acetyl cysteine (NAC) (Figure 6, E and F). By contrast, the short-term administration of GKT137831 had no effect on insulin-induced p-AKT, arguing against the idea that NOX4-derived ROS had direct effects on insulin signaling (Supplemental Figure 4D). Therefore, defective NOX4-dependent antioxidant defense may exacerbate mitochondrial oxidative stress and the attenuation of insulin signaling in hepatocytes.

A potential outcome of defective antioxidant defense in NOX4-deficient hepatocytes in NAFLD might be increased oxidative damage. Consistent with this, we noted increased oxidative lipid damage, as assessed by immunoblotting NOX4-deficient hepatocytes from HFD-fed mice for 4-hydroxynonenal (4-HNE) (Figure 5E and Supplemental Figure 4E), a marker of lipid peroxidation, as well as increased oxidative protein damage, as assessed by immunoblotting for protein carbonylation (Figure 5F and Supplemental Figure 4F). The increased lipid peroxidation and protein carbonylation exceeded that otherwise induced in HFD-fed mice (Figure 5, E and F). The increased oxidative protein damage was attenuated when NOX4-deficient *Alb*-Cre *Nox4^fl/fl^* hepatocytes were cultured with sulforaphane (Figure 5G), which could rescue the defective antioxidant defense (Figure 4, A and B). Moreover, the increased oxidative protein damage was attenuated when NOX4-deficient *Alb*-Cre *Nox4^fl/fl^* hepatocytes were cultured in the presence of mitoTEMPOL (Figure 5H). Finally, the sustained inhibition of NOX4 with GKT137831 also increased oxidative protein damage in hepatocytes from HFD-fed mice (Figure 6G), and this could be corrected when cells were cocultured with sulforaphane (Figure 6H) or NAC (Figure 6I). These findings are consistent with the idea that the increased oxidative damage accompanying NOX4 deficiency was attributable to defective antioxidant defense and increased mitochondrial oxidative stress.

**Figure 7:**
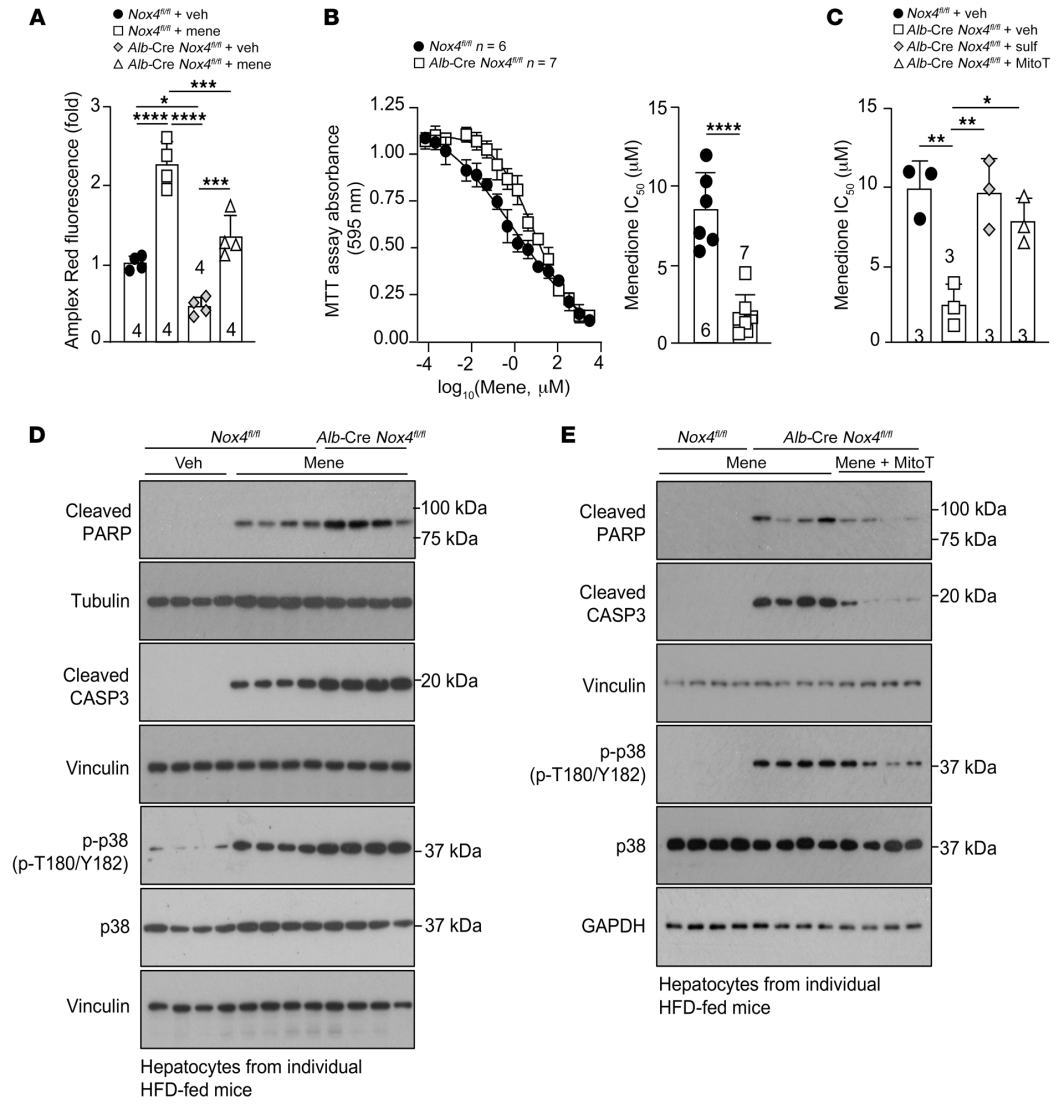
NOX4 deficiency promotes cell death. (**A**–**D**) *Nox4^fl/fl^* and *Alb*-Cre *Nox4^fl/fl^* mice were fed a HFD for 8–10 weeks, and hepatocytes were isolated and (**A**) treated with vehicle (veh) (EtOH) or 1 μM menadione (mene) for 16 hours and processed for H_2_O_2_ measurement, or (**B**) treated with varying concentrations of menadione (16.9 μM to 3 mM) for 48 hours or (**C**) with vehicle, 1 μM sulforaphane, or 20 μM MitoT for 16 hours and then with varying concentrations of menadione for 48 hours in the presence of 1 μM sulforaphane or 20 μM MitoT as indicated and (**B** and **C**) processed for the analysis of cell death using the 3-(4,5-dimethylthiazol-2-yl)-2,5-diphenyltetrazolium bromide (MTT) assay. (**D** and **E**) Alternatively, hepatocytes were incubated with (**D**) vehicle or 2.5 μM menadione for 36 hours or (**E**) vehicle or 20 μM MitoT for 16 hours and then 2.5 μM menadione for 24 hours in the presence of vehicle or 20 μM MitoT, as indicated, and then processed for immunoblotting to monitor for cleaved PARP, cleaved caspase-3 (CASP3), and p-p38 (T180/Y182) MAPK. Representative and quantified results are shown as the mean ± SEM for the indicated number of mice. *P < 0.05, **P < 0.01, ***P < 0.001, and ****P < 0.0001, by (**A** and **C**) 2-way ANOVA or (**B**) Student’s *t* test.

To further test whether the increased oxidative damage could be attributed to enhanced oxidant sensitivity, we treated hepatocytes from HFD-fed *Nox4^fl/fl^* versus *Alb*-Cre *Nox4^fl/fl^* mice with menadione. Menadione is a drug that generates ROS within the cytosol and mitochondria through futile redox cycling (48). Menadione increased the generation of ROS/H_2_O_2_ in hepatocytes isolated from HFD-fed mice (Figure 7A); overall H_2_O_2_ measured was reduced by NOX4 deficiency, but the relative increase in menadione-induced H_2_O_2_ was similar (Figure 7A). As expected, menadione treatment increased oxidative protein damage in both *Nox4^fl/fl^* and *Alb*-Cre *Nox4^fl/fl^* hepatocytes (Supplemental Figure 4G). However, the extent of oxidative damage was exacerbated by NOX4 deficiency (Supplemental Figure 4G). A potential consequence of increased oxidative damage is cell death, which in the context of the liver would elicit regenerative responses and lead to fibrosis. Consistent with this, we found that menadione, which promotes cell death in a ROS-dependent manner (48), induced cell death (assessed by monitoring for metabolically active live cells) and this was exacerbated by NOX4 deficiency (IC_50_ *Nox4^fl/fl^* = 8.4 μM; IC_50_ *Alb*-Cre *Nox4^fl/fl^* = 1.3 μM) (Figure 7B). Importantly, the enhanced menadione-induced cell death was blocked by sulforaphane or mitoTEMPOL (Figure 7C), indicating that NOX4 deficiency exacerbated cell death because of defective NFE2L2 antioxidant defense and increased mitochondrial ROS. Moreover, the enhanced menadione-induced cell death in NOX4-deficient hepatocytes was accompanied by increased cleaved caspase-3 and cleaved poly ADP ribose polymerase (PARP), consistent with the promotion of apoptosis (Figure 7D). The enhanced cell death was also accompanied by increased phosphorylation and activation of the mitogen-activated protein kinases JNK and p38 (Figure 7D and Supplemental Figure 4H), which are known to contribute to apoptosis and inflammatory responses in the liver (49, 50). Finally, the enhanced cleavage of caspase-3 and PARP and the increased p38 activation were reduced by treating hepatocytes with either mitoTEMPOL (Figure 7E) or the NFE2L2 agonist sulforaphane (Supplemental Figure 4I). These results point toward the idea that induction of NOX4 in the liver in obesity drives the antioxidant defense response to mitigate mitochondrial oxidative stress, macromolecular damage, and cell death in lipid-laden hepatocytes.

### Hepatocyte NOX4 deficiency promotes obesity, steatosis, and insulin resistance in mice.

**Figure 8:**
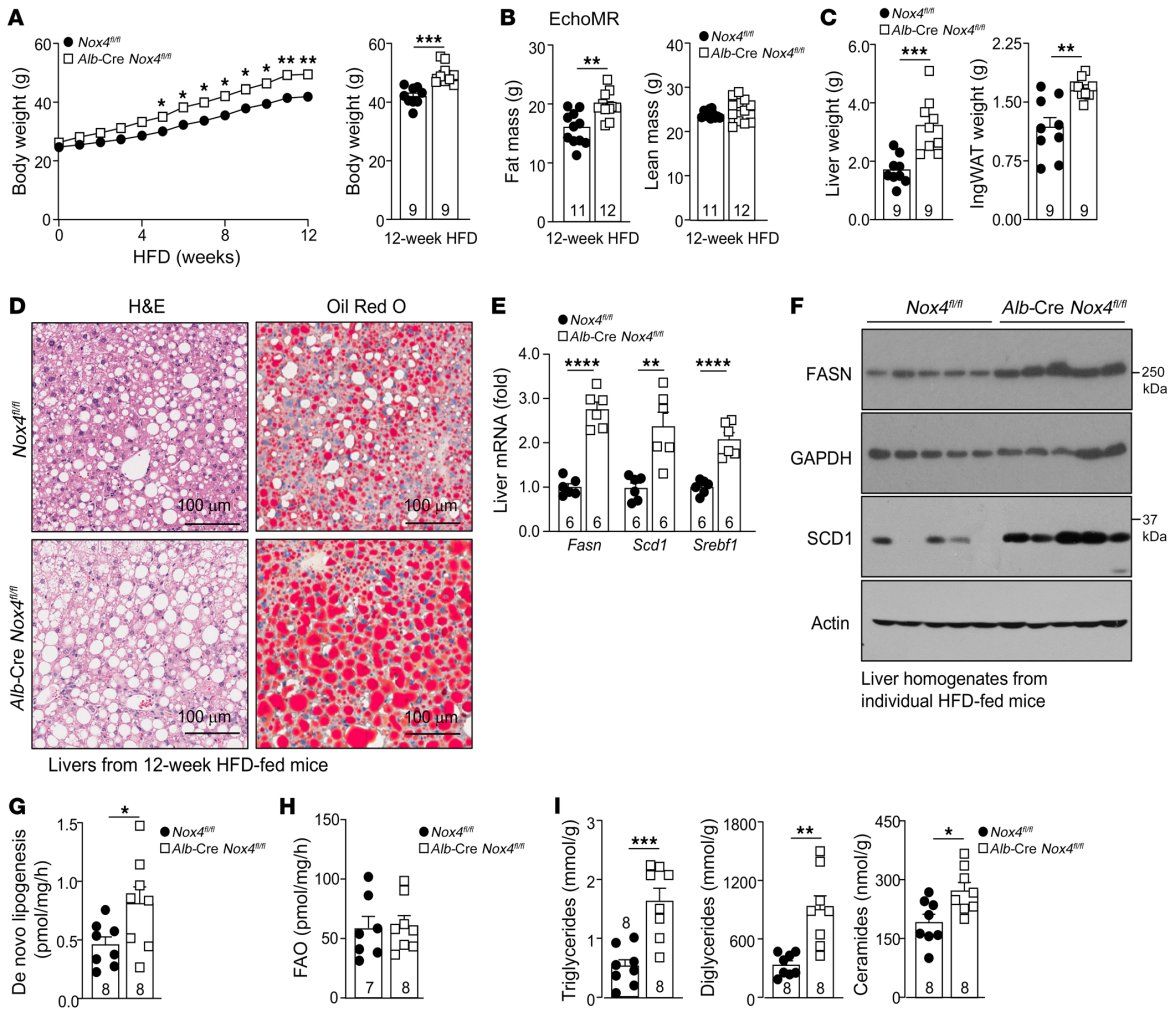
NOX4 deficiency promotes obesity, steatosis, and insulin resistance. *Nox4^fl/fl^* and *Alb*-Cre *Nox4^fl/fl^* male mice were fed a HFD for 12 weeks. (**A**) Body weights, (**B**) body composition, and (**C**) liver and inguinal white adipose (IngWAT) tissue weights. (**D**) Livers were processed for histology (H&E or Oil Red O). Scale bars: 100 μm. (**E**–**I**) Livers were processed for (**E**) qPCR, (**F**) immunoblotting, or biochemical assays to measure (**G**) de novo lipogenesis, (**H**) fatty acid oxidation (FAO), as well as (**I**) triglyceride, diglyceride, and ceramide levels. Representative and quantified results are shown as the mean ± SEM for the indicated number of mice. *P < 0.05, **P < 0.01, ***P < 0.001, and ****P < 0.0001, by 2-way ANOVA (**A**), Student’s *t* test (12-week body weight) (**A**), or Student’s *t* test (**B**, **C**, **E**, and **G**–**I**).

To explore the effect of NOX4 abundance on hepatic pathophysiology in diet-induced obesity (DIO), we sought to delete *Nox4* postnatally in hepatocytes using the *Alb*-Cre transgene; *Nox4* was efficiently deleted in the livers and hepatocytes of *Alb*-Cre *Nox4^fl/fl^* mice (Figure 3, A and B, and Supplemental Figure 5, A and B), but not in other metabolic tissues (Supplemental Figure 5, A and B). Eight-week-old *Nox4^fl/fl^* and *Alb*-Cre *Nox4^fl/fl^* mice were fed either a standard chow diet or a HFD for 12 weeks, and the effects on body weight, body composition, glucose metabolism, and liver steatosis were assessed. The deletion of *Nox4* in hepatocytes had no effect on body weight in mice fed a chow diet (Supplemental Figure 5C). By contrast, the deletion of hepatocyte *Nox4* in HFD-fed male (Figure 8A) or female (Supplemental Figure 6A) mice led to an increase in body weight. The increased body weight in male mice was accompanied by increased food intake in the dark cycle, but paradoxically also increased energy expenditure, as determined by indirect calorimetry (Supplemental Figure 7, A–C, and Supplemental Figure 8). NOX4 deficiency increased whole-body adiposity and fat pad weights in HFD-fed but not chow-fed mice, without any change in lean mass (Figure 8, B and C, Supplemental Figure 5, D and E, and Supplemental Figure 6B). Liver weights were also increased in HFD-fed mice (Figure 8C; Supplemental Figure 6C) but not chow-fed *Alb*-Cre *Nox4^fl/fl^* mice (Supplemental Figure 5E), and this was accompanied by increased steatosis, as assessed by H&E histology for lipid droplets and staining for neutral lipids with Oil Red O (Figure 8D and Supplemental Figure 6D); steatosis was not evident in chow-fed *Alb*-Cre *Nox4^fl/fl^* mice (Supplemental Figure 5F). The increased steatosis was in turn accompanied by the increased expression of de novo lipogenesis genes, including *Fasn*, *Scd1*, and *Srebf1* (Figure 8E), increased hepatic FASN and SCD1 protein levels (Figure 8F), and an approximately 2-fold increase in de novo lipogenesis (Figure 8G), as determined in liver slices ex vivo, without any significant change in fatty acid oxidation (Figure 8H). The enhanced de novo lipogenesis was consistent with studies showing that NFE2L2 negatively regulates lipid synthesis genes in the liver and protects against steatosis (35, 36, 51–53). Indeed, we found that expression of the lipogenesis genes *Fasn* and *Scd1* was also induced in GKT137831-treated hepatocytes (Supplemental Figure 9A) from HFD-fed mice, in which antioxidant defense was defective (Figure 6A). However, the enhanced expression of lipogenic genes in NOX4-deficient hepatocytes from HFD-fed mice was corrected by administration of the NFE2L2 agonist sulforaphane (Supplemental Figure 9B). Nonetheless, consistent with the overt steatosis, liver triglyceride, diglyceride, and ceramide levels were significantly elevated in HFD-fed *Alb*-Cre *Nox4^fl/fl^* mice (Figure 8I). Therefore, the deletion of NOX4 in hepatocytes exacerbated DIO and promoted steatosis that was attributable, at least in part, to increased de novo lipogenesis.

**Figure 9:**
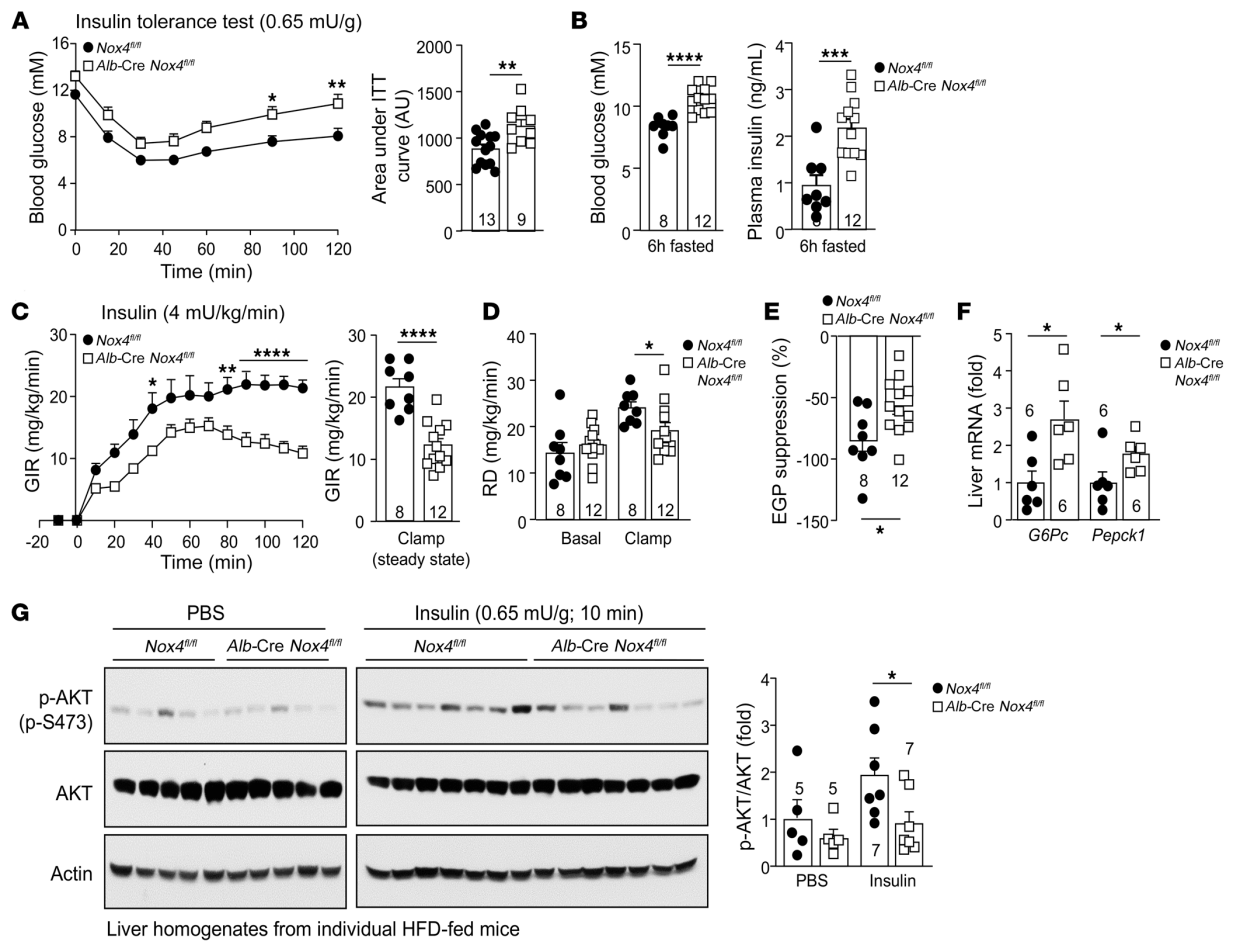
NOX4 deficiency promotes insulin resistance. (**A**–**G**) *Nox4^fl/fl^* and *Alb*-Cre *Nox4^fl/fl^* male mice were fed a HFD for 12 weeks. (**A**) Mice were subjected to insulin tolerance tests (ITTs) (areas under the ITT curves were determined; AU are shown) or fasted for 6 hours, followed by (**B**) blood glucose and plasma insulin level measurements, or mice were subjected to hyperinsulinemic-euglycemic clamps. (**C**) GIRs, (**D**) RD, and (**E**) EGP (percentage of suppression). (**F**) Clamped livers were processed for qPCR. (**G**) Mice were fasted for 6 hours, injected with PBS or insulin, and livers were processed for immunoblotting. Representative and quantified results are shown as the mean ± SEM for the indicated number of mice. *P < 0.05, **P < 0.01, ***P < 0.001, and ****P < 0.0001, by (**A**–**G**) Student’s *t* test, or (**A** and **C**) 2-way ANOVA (ITT curves and GIR time courses).

An expected outcome of DIO in C57BL/6 mice is the development of insulin resistance. Consistent with this, we found that HFD-fed *Alb*-Cre *Nox4^fl/fl^* mice were more insulin resistant, as reflected in insulin tolerance tests (Figure 9A and Supplemental Figure 6, E and F) and the heightened circulating glucose and insulin levels in fasted mice (Figure 9B and Supplemental Figure 6F). To explore the effect of NOX4 deficiency on glucose turnover and whole-body insulin sensitivity, we subjected conscious, free-moving *Nox4^fl/fl^* and *Alb*-Cre *Nox4^fl/fl^* male mice fed a HFD for 12 weeks to hyperinsulinemic-euglycemic clamps. We found that the glucose infusion rate (GIR) necessary to maintain euglycemia during the insulin clamp was reduced (Figure 9C and Supplemental Figure 7, D and E), consistent with the development of insulin resistance. The rate of glucose disappearance (RD), a measure of muscle and adipose tissue glucose uptake, was reduced in clamped *Alb*-Cre *Nox4^fl/fl^* mice (Figure 9D), whereas endogenous glucose production (EGP) in clamped *Alb*-Cre *Nox4^fl/fl^* mice was increased (Figure 9E and Supplemental Figure 7, F and G), consistent with hepatic insulin resistance. The latter was accompanied by an increased expression of genes encoding glucose-6 phosphatase (*G6pc*) and phosphoenolpyruvate carboxykinase (*Pck1*) (Figure 9F), which are rate-limiting enzymes in gluconeogenesis. The diminished insulin-induced repression of HGP in HFD-fed *Alb*-Cre *Nox4^fl/fl^* mice was also associated with a reduction in insulin-induced PI3K/AKT signaling, as assessed by immunoblotting for AKT (Ser473) phosphorylation in corresponding liver homogenates (Figure 9G), or in hepatocytes from HFD-fed *Alb*-Cre *Nox4^fl/fl^* mice (Figure 5, B–D). Taken together, our results show that NOX4 deficiency in the liver diminished antioxidant defense to promote hepatic and systemic insulin resistance in obesity.

### Hepatocyte NOX4 deficiency promotes NASH and fibrosis.

**Figure 10:**
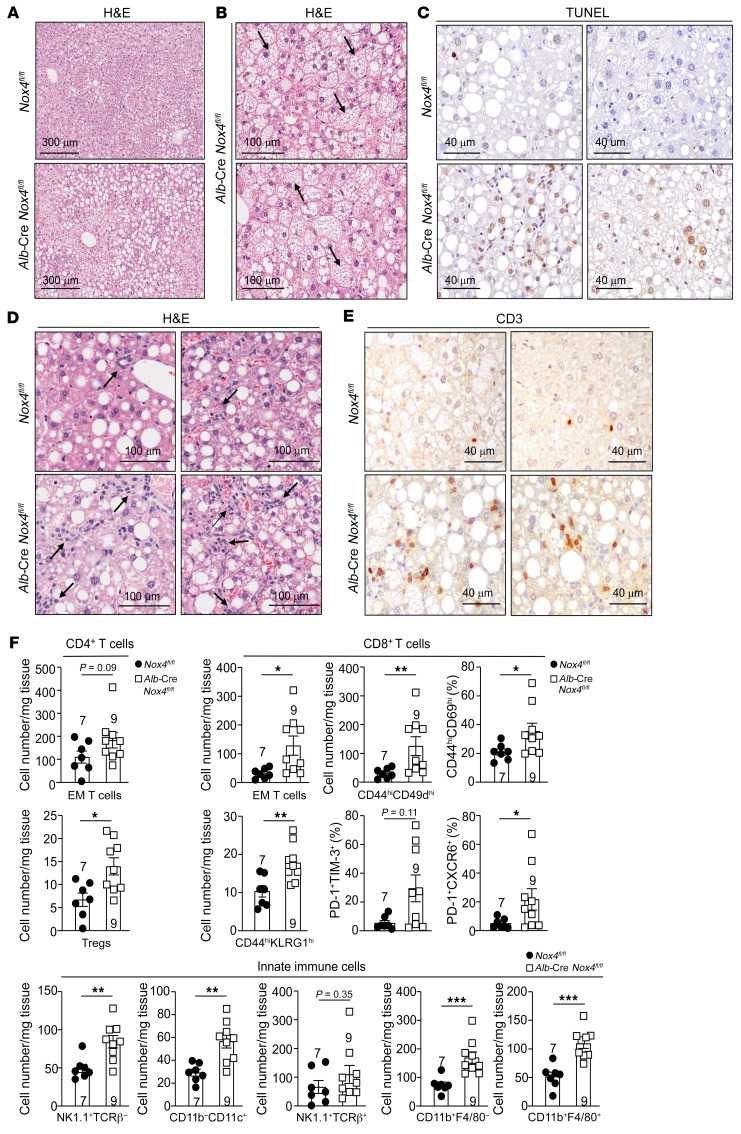
NOX4 deficiency promotes NASH. (**A**–**H**) *Nox4^fl/fl^* and *Alb*-Cre *Nox4^fl/fl^* male mice were fed a HFD for 12 weeks. (**A**–**E**) Livers were processed for (**A**, **B**, and **D**) histology (H&E) to monitor (**A**) steatosis and (**B**) hepatocyte ballooning (indicated by arrows), or (**D**) lymphocytic infiltrates (indicated by arrows) or (**C** and **E**) immunohistochemistry to monitor for (**C**) TUNEL^+^ apoptotic cells or (**E**) CD3^+^ T cells. (**F**) Liver lymphocytes including CD44^hi^CD62L^lo^ CD4^+^ and CD8^+^ effector/memory (EM) T cells, CD4^+^CD25^+^FoxP3^+^ Tregs, CD8^+^CD44^hi^CD49d^hi^ T cells, CD8^+^CD44^hi^CD69d^hi^ T cells, CD8^+^CD44^hi^KLRG1^hi^ T cells, CD8^+^PD-1^hi^TIM-3^hi^ T cells, CD8^+^PD-1^hi^CXCR6^hi^ T cells, NK1.1^+^TCRβ^–^ NK cells, NK1.1^+^TCRβ^+^ NK T cells, CD11b^–^CD11c^+^ lymphoid DCs, CD11b^+^F4/80^–^ hepatic macrophages, and CD11b^+^F4/80^+^ Kupffer cells were analyzed by flow cytometry. Representative and quantified results are shown as the mean ± SEM for the indicated number of mice. *P < 0.05, **P < 0.01, and ***P < 0.001, by 2-tailed Mann-Whitney *U* test (**F**).

**Figure 11:**
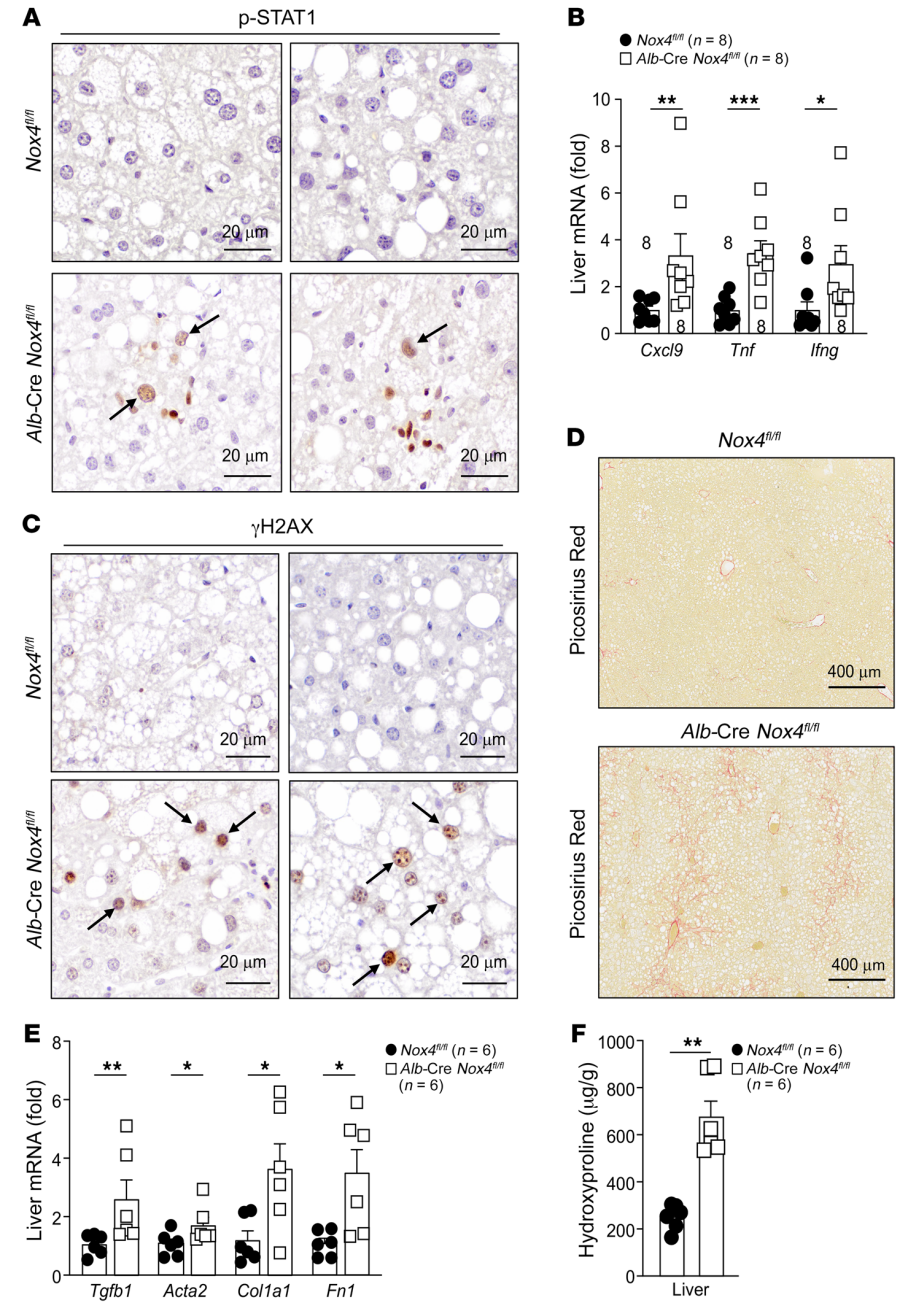
NOX4 deficiency promotes inflammation and fibrosis. (**A**–**F**) *Nox4^fl/fl^* and *Alb*-Cre *Nox4^fl/fl^* male mice were fed a HFD for 12 weeks. (**A**) Livers were processed for immunohistochemistry to monitor for STAT1 (Tyr701) phosphorylation (p-STAT1; p-STAT1^+^ hepatocytes indicated by arrows). (**B**) Liver *Cxcl9*, *Tnf*, and *Ifng* mRNA levels were assessed by qPCR. (**C**) Livers were processed for immunohistochemistry to monitor for DNA damage (γH2AX^+^ hepatocytes indicated by arrows). (**D**) Livers were processed for Picrosirius Red staining to monitor for fibrosis. (**E**) Liver *Acta2*, *Tgfb*, *Col1a1*, and *Fn1* mRNA levels were assessed by qPCR. (**F**) Liver hydroxyproline levels. Scale bars: 300 μm (**A**), 40 μm (**C** and **E**), and 100 μm (**B** and **D**). Representative and quantified results are shown as the mean ± SEM for the indicated number of mice. *P < 0.05, **P < 0.01, and ***P < 0.001, by Student’s *t* test (**B**, **E**, and **F**).

C57BL/6 mice fed a HFD become obese and develop NAFL, but do not develop NASH (54). Beyond exacerbating steatosis and insulin resistance, hepatocyte NOX4 deficiency in HFD-fed C57BL/6 mice also facilitated the progression to NASH (Figure 7), with many of the key diagnostic features of human NASH (1), including hepatocyte ballooning (Figure 10, A and B), hepatocyte cell death, as reflected by TUNEL staining (Figure 10C), and lymphocytic infiltration, including CD3^+^ T cell infiltration, as assessed by histology (Figure 10D), immunohistochemistry (Figure 10E), and flow cytometry (Figure 10F and Supplemental Figure 10). The lymphocytic infiltrates included CD4^+^ and CD8^+^ T cells with an effector/memory phenotype (CD44^hi^CD62L^lo^), including antigen-experienced (CD49d^hi^) and activated (CD69^hi^) CD8^+^ T cells, as well as those with terminally differentiated (KLRG1^hi^) and exhausted (PD-1^hi^TIM-3^hi^) phenotypes (Figure 10F and Supplemental Figure 10), as seen previously in humans with NASH (32, 54–56). It also included “autoaggressive” PD-1^hi^CXCR6^hi^ CD8^+^ T cells (Figure 10F), which have been proposed to contribute to NAFLD pathogenesis in humans (57). NK cells, DCs, NK T (NKT) cells, macrophages, and Kupffer cells that are frequently elevated in NASH (56) were also increased (Figure 10F and Supplemental Figure 10), as were immunosuppressive CD4^+^ Tregs (Figure 10F). The increased T cell infiltration was in turn accompanied by increased inflammation, as reflected by heightened p-STAT1 (Tyr701) in hepatocytes (Figure 11A), expression of the STAT1 target gene *Cxcl9* (encodes the T cell chemoattractant CXCL9), and increased expression of genes encoding proinflammatory cytokines, including IFN-γ (*Ifng*) and TNF (*Tnf*) (Figure 11B). An expected consequence of T cell recruitment and activation, inflammation, and hepatocyte death in NAFLD is the induction of reparative processes that lead to DNA damage in dividing hepatocytes and increased fibrosis due to hepatic stellate cell activation. Consistent with this, hepatocytes with double-stranded DNA (dsDNA) breaks (as assessed by γH2AX staining) were readily evident in the livers of HFD-fed *Alb*-Cre *Nox4^fl/fl^* mice (Figure 11C). In addition, the livers of HFD-fed *Alb*-Cre *Nox4^fl/fl^* mice showed overt signs of fibrosis, as reflected by Picrosirius red staining (stains collagen) (Figure 11D), increased expression of fibrosis-related genes, including α–smooth muscle actin (*Acta2*) and transforming growth factor β (*Tgfb*), indicative of stellate cell activation, and expression of the extracellular matrix genes α-1 type 1 collagen (*Col1a1*) and fibronectin (*Fn1*) (Figure 11E), as well as an increased abundance of hydroxyproline (Figure 11F), a measure of collagen degradation and severity of fibrosis. Thus, the deletion of NOX4 in the liver not only promoted steatosis but also facilitated the transition to NASH and ensuing fibrosis in DIO.

**Figure 12:**
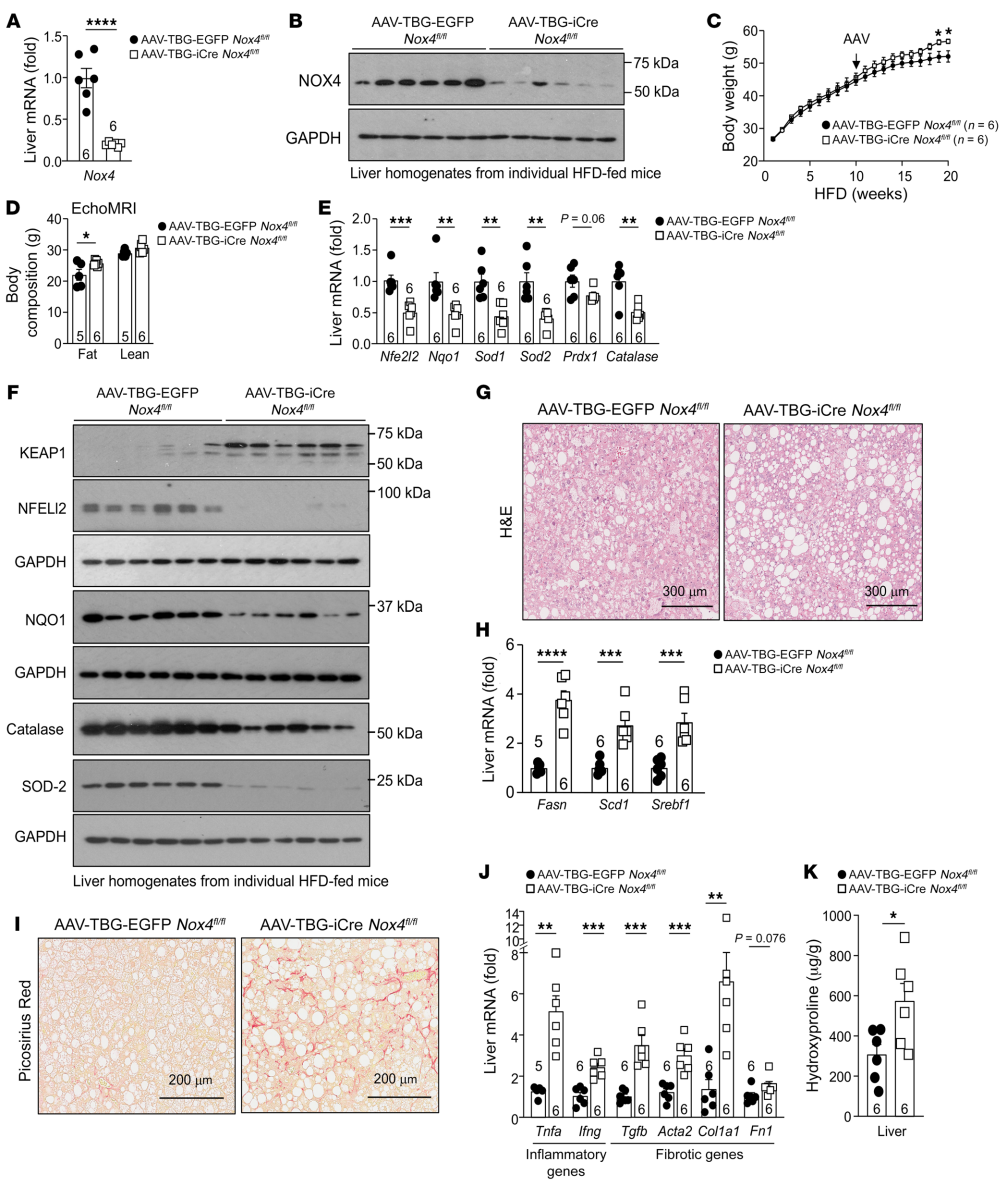
NOX4 deficiency in obese adult mice promotes NASH and fibrosis. (**A**–**I**) Male *Nox4^fl/fl^* mice fed a HFD for 10 weeks were administered i.v. AAV-TBG-iCre or AAV-TBG-EGFP and then fed a HFD for another 10 weeks. Livers were processed for (**A**) qPCR or (**B**) immunoblotting. (**C**) Body weights. (**D**) Body composition. Livers were processed for (**E**) qPCR and (**F**) immunoblotting to monitor for antioxidant defense; (**G**) histology (H&E) and (**H**) qPCR to monitor for steatosis; and (**I**) histology (Picrosirius red) and (**J**) qPCR to monitor for fibrosis and inflammation. (**K**) Liver hydroxyproline levels. Representative results from at least 2 independent experiments are shown as the mean ± SEM. Scale bars: 300 μm (**G**) and 200 μm (**I**). *P < 0.05, **P < 0.01, ***P < 0.001, and ****P < 0.0001, by Student’s *t* test (**A**, **C**–**E**, **H**, **J**, and **K**).

Next, we sought to determine whether NOX4 deletion might be sufficient to drive NASH and fibrosis in adult mice that were already obese and had established steatosis. To this end, we deleted *Nox4* using an adeno-associated virus (AAV) thyroxine-binding globulin (TBG) promoter construct that expressed Cre specifically in hepatocytes (58). Eight-week-old *Nox4^fl/fl^* mice were fed a HFD for 10 weeks to promote obesity and steatosis and then administered either AAV-TBG-EGFP control or AAV-TBG-iCre, with HFD feeding continued for a further 10 weeks. The administration of AAV-TBG-iCre efficiently deleted *Nox4* so that *Nox4* mRNA was reduced by approximately 70% and NOX4 protein by 60% (Figure 12, A and B). The deletion of *Nox4* was accompanied by modest increases in body weight (Figure 12C) attributed to increased whole-body adiposity (Figure 12D). The deletion of *Nox4* was also accompanied by decreased hepatic expression of antioxidant defense genes, including *Nfe2l2*, *Nqo1*, *Sod1*, *Sod2*, *Prdx1*, and *Cat* (Figure 12E), and reduced NFE2L2 (Figure 12F), increased KEAP1 (Figure 12F), and decreased NQO1, SOD2, and catalase protein expression (Figure 12F), consistent with an abrogated antioxidant defense response. Moreover, *Nox4* deletion increased steatosis (Figure 12G) and the expression of lipogenic genes (Figure 12H). Notably, the deletion of hepatocyte *Nox4* in adult obese mice also facilitated the transition to NASH with fibrosis, as assessed histologically by Picrosirius red staining (Figure 12I) and by measuring hepatic expression of inflammatory (*Tnf*, *Ifng*) and fibrosis-related (*Acta2*, *Tgfb*, *Col1a1*, *Fn1*) genes (Figure 12J) and hepatic hydroxyproline levels (Figure 12K). Therefore, the deletion of hepatocyte *Nox4* in adult obese mice could abrogate the antioxidant defense response otherwise induced during NAFL to exacerbate steatosis and facilitate the transition to NASH and fibrosis.

**Figure 13:**
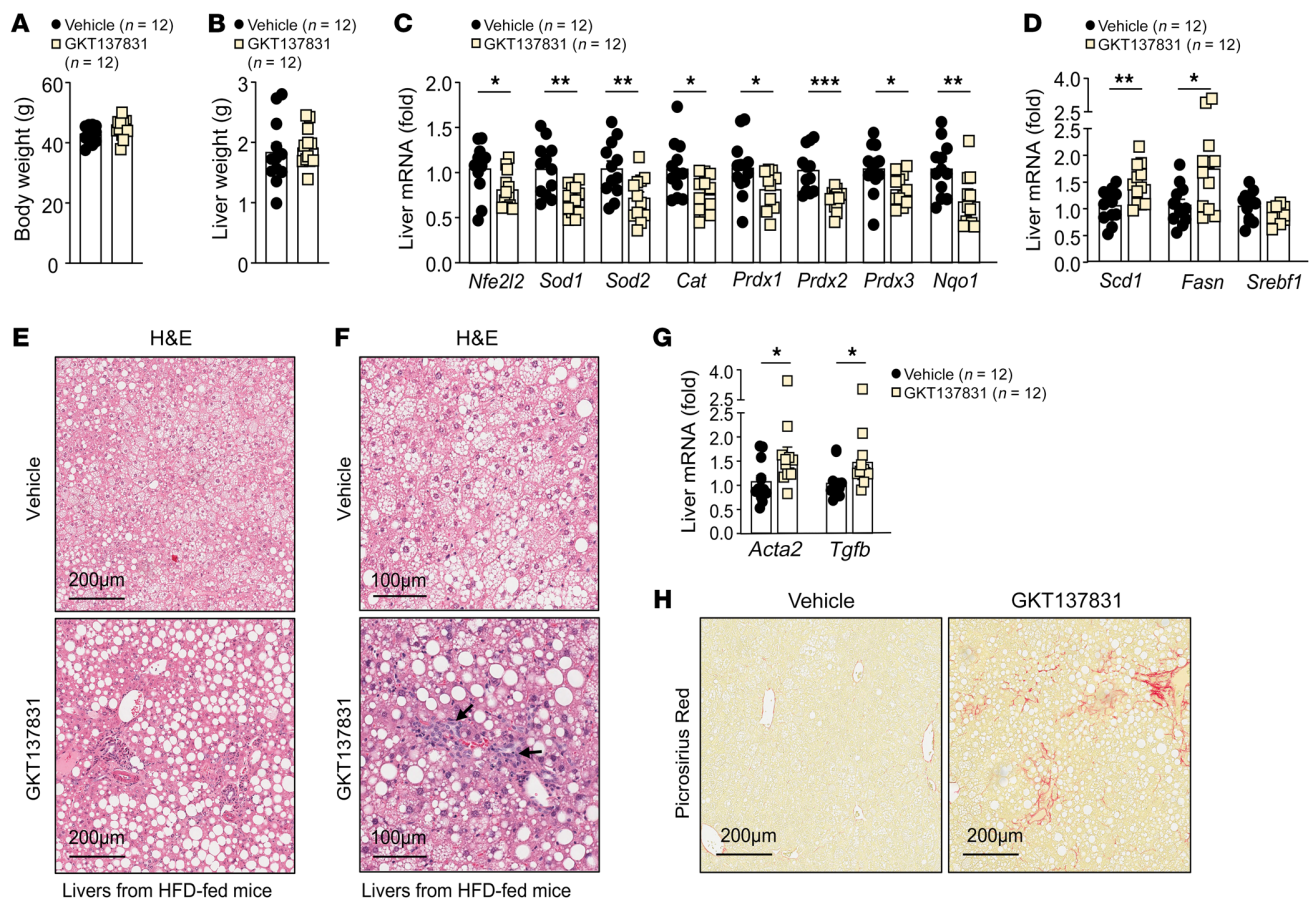
NOX4 inhibition promotes NASH and fibrosis in HFD-fed mice. (**A**–**F**) C57BL/6 male mice were fed a HFD for 15 weeks and administered GKT137831 by oral gavage (40 mg/kg) 3 times per week for 5 weeks. (**A**) Body weights. (**B**) Liver weights. Livers were processed for (**C** and **D**) qPCR to assess the expression of (**C**) antioxidant defense and (**D**) lipogenic genes. Livers were processed for (**E** and **F**) H&E staining to monitor for (**E**) steatosis and (**F**) lymphocytic infiltrates (indicated by arrows). Livers were processed for (**G**) qPCR to assess the expression of fibrosis-related genes or (**H**) for histology (Picrosirius red). Scale bars: 100 μm (**F**) and 200 μm (**E** and **H**). Representative and quantified results are shown as the mean ± SEM for the indicated number of mice. *P < 0.05, **P < 0.01, and ***P < 0.001, by Student’s *t* test (**A**–**D** and **G**).

To explore whether the progression to NASH and fibrosis associated with the deletion of *Nox4* might be ascribed to decreased NOX4-derived ROS generation and antioxidant defense, we examined whether the NOX1/4 inhibitor GKT137831 could promote NASH and fibrosis in HFD-fed C57BL/6 mice (Figure 13). The administration of GKT137831 (40 mg/kg, 3 times/week for 5 weeks) to obese C57BL/6 mice fed a HFD for 15 weeks modestly increased body weights (Figure 13A) and had no significant effect on liver weights (Figure 13B), but decreased *Nfe2l2* expression in the liver along with the expression of key antioxidant defense genes, including *Sod1*, *Sod2*, *Cat*, and *Nqo1* (Figure 13C), while increasing the expression of lipogenic genes (Figure 13D and Supplemental Figure 9A). This was accompanied by increased steatosis (Figure 13E) and lymphocytic infiltrates (Figure 13F), as well as by increased fibrosis (Figure 13, G and H), as determined by the hepatic expression of fibrosis-related genes (Figure 13G) and by histology (Figure 13H). These results are consistent with the notion that the induction of NOX4 in the livers of obese mice with steatosis contributes to the ROS-dependent induction of antioxidant defense to limit steatosis, mitochondrial oxidative stress, and tissue damage to thereby limit the progression to NASH and fibrosis.

### Reduced NOX4 and antioxidant defense gene expression in NASH with advanced fibrosis.

**Figure 14:**
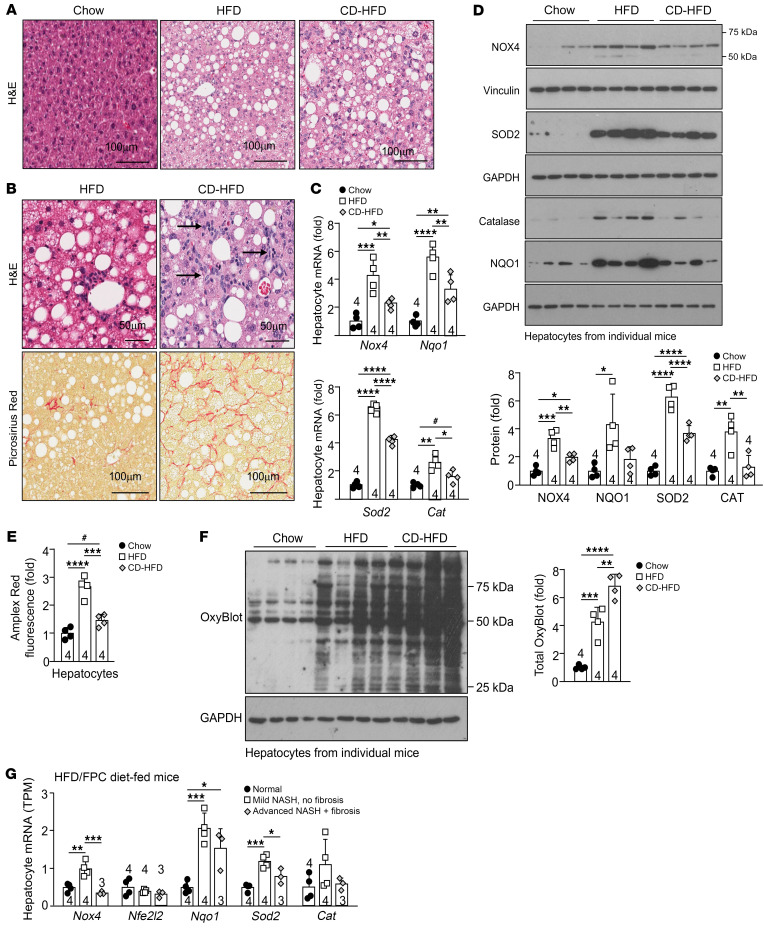
Reduced NOX4 and antioxidant defense gene expression in NASH with advanced fibrosis. (**A**–**F**) C57BL/6 male mice were fed a chow-diet, a HFD, or a CD-HFD for 12 weeks. Livers were processed for histology (**A**) and stained with H&E to monitor for steatosis or (**B**) lymphocytic infiltrates (indicated by arrows), or were stained with Picrosirius red to monitor for fibrosis. (**C**–**F** Hepatocytes were isolated and processed for (**C**) qPCR, (**D**) immunoblotting, (**E**) H_2_O_2_ measurements, or (**F**) protein carbonylation analysis. (**G**) RNA-Seq analysis (GSE162876) of hepatocyte nuclei from hepatocyte–isolation of nuclei tagged in specific cell types (HEP-INTACT) mice (59) fed a low-fat control diet (*n* = 4) versus a HFD rich in fructose, PA, and cholesterol (HFD/FPC diet) for 20 weeks; HFD/FPC diet–fed mice were stratified into groups of those with mild fibrosis (*n* = 4) or overt inflammation and advanced fibrosis (*n* = 3). Scale bars: 50 μm and 100 μm (**A** and **B**). Representative and quantified results are shown as the mean ± SEM for the indicated number of mice. *P < 0.05, **P < 0.01, ***P < 0.001, and ****P < 0.0001, by 1-way ANOVA (**C**–**G**); ^#^*P* < 0.05, by Student’s *t* test (**C** and **E**) TPM, transcripts per kilobase million.

**Figure 15:**
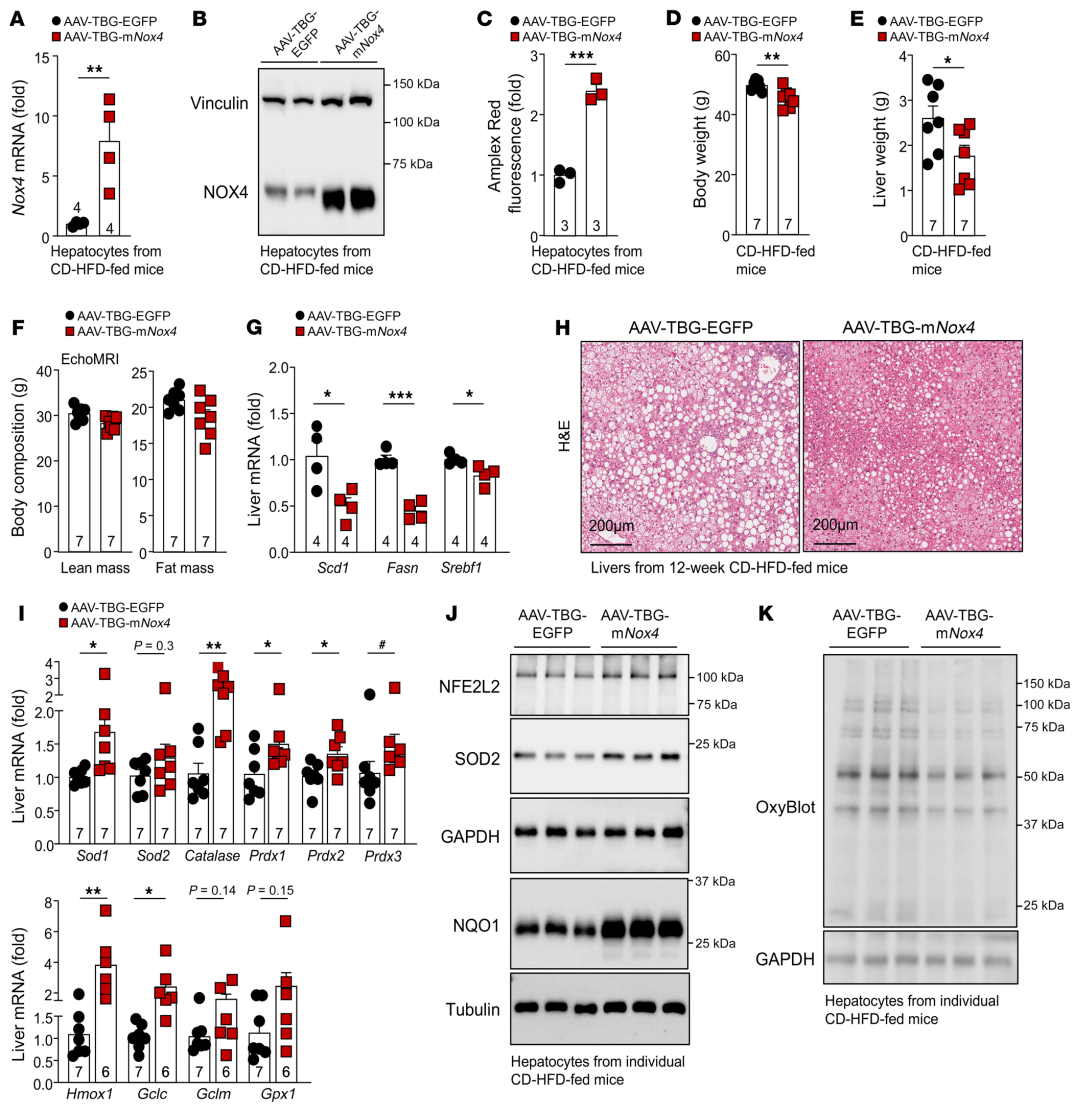
NOX4 overexpression promotes antioxidant defense and tempers steatosis and oxidative damage. C57BL/6 male mice were administered (i.v.) AAV-TBG-EGFP or AAV-TBG-m*Nox4* and fed a CD-HFD for up to 12 weeks. (**A**–**C**) Hepatocytes were isolated and processed for (**A**) qPCR, (**B**) immunoblotting, or (**C**) extracellular H_2_O_2_ measurements. (**D**) Body weights, (**E**) liver weights, and (**F**) body composition of mice fed a CD-HFD for 12 weeks. Livers were processed for (**G**) qPCR to assess the expression of lipogenic genes, (**H**) histology to monitor for steatosis, or (**I**) qPCR to assess antioxidant defense gene expression. (**J**–**K**) Hepatocytes were isolated and (**J**) the abundance of antioxidant defense proteins and (**K**) protein carbonylation (OxyBlot) were assessed by immunoblotting. Scale bars: 200 μm (**H**). Representative and quantified results are shown as the mean ± SEM for the indicated number of mice. *P < 0.05, **P < 0.01, and ***P < 0.001, by Student’s *t* test (**C**–**E**, **G**, and **I**); ^#^*P* < 0.05, by 2-tailed Mann-Whitney *U* test (**I**).

**Figure 16:**
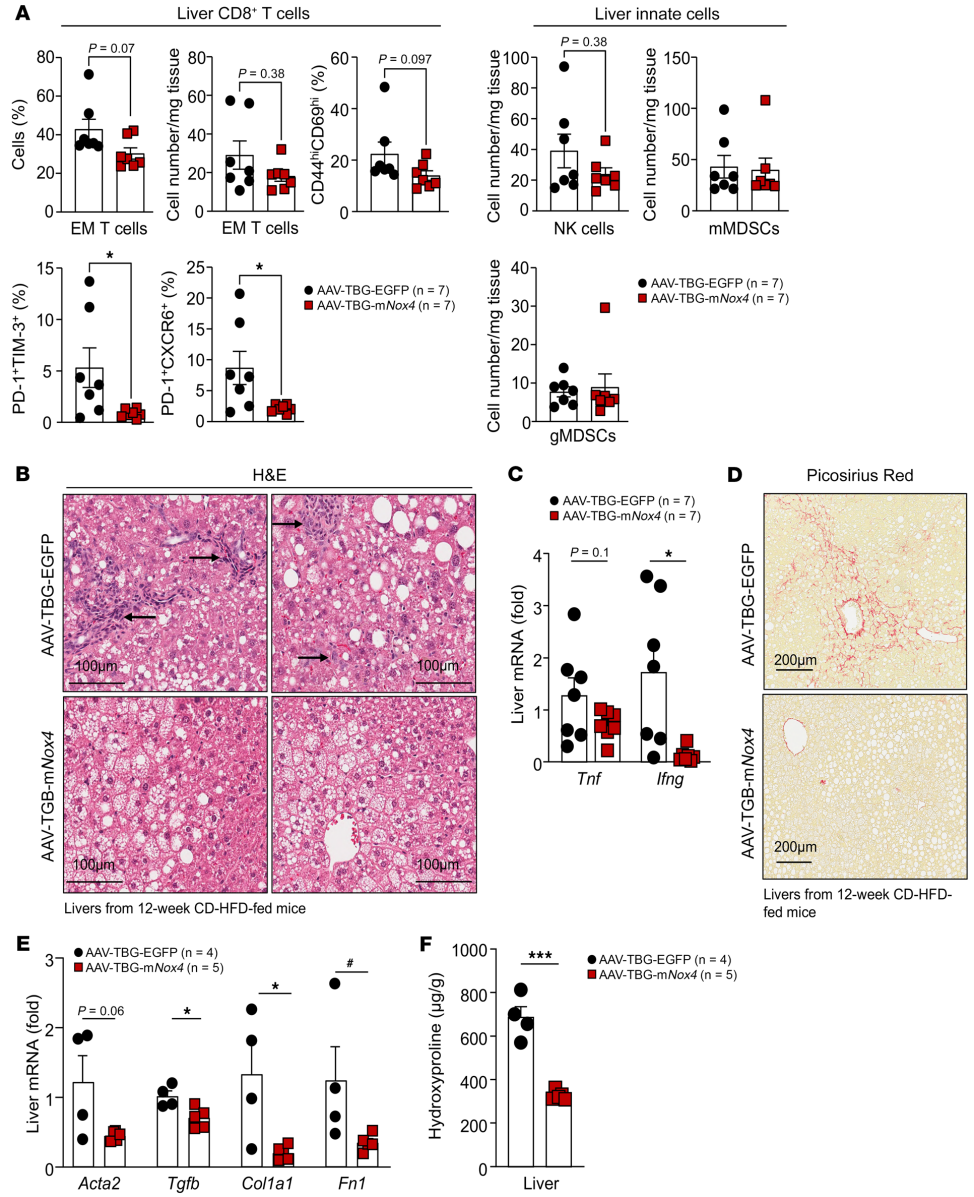
NOX4 overexpression tempers NASH and fibrosis. C57BL/6 male mice were administered AAV-TBG-EGFP or AAV-TBG-m*Nox4* and fed a CD-HFD for 12 weeks. (**A**) Liver lymphocytes including CD8^+^ EM T cells, CD8^+^CD44^hi^CD69^hi^ T cells, CD8^+^PD-1^hi^TIM-3^hi^ T cells, CD8^+^PD-1^hi^CXCR6^hi^ T cells, NK1.1^+^TCRβ^–^ NK cells, monocytic myeloid-derived CD11b^+^F4/80^hi/lo^Ly6C^+^Ly6G^–^ (mMDSCs), and granulocytic myeloid-derived CD11b^+^F4/80^hi/lo^Ly6C^+^Ly6G^+^ (gMDSCs) suppressor cells were analyzed by flow cytometry. Livers were processed for (**B**) histology or (**C**) qPCR to monitor for immune cell infiltrates (H&E; lymphocytes are indicated by arrows) or inflammation (*Ifng* and *Tnf*). Livers were processed for (**D**) histology (Picrosirius red), (**E**) qPCR, or (**F**) measurement of hydroxyproline levels to monitor for fibrosis. Scale bars: 100 μm (**B**) and 200 μm (**D**). Representative and quantified results are shown as the mean ± SEM for the indicated number of mice. *P < 0.05 and ***P < 0.001, by Student’s *t* test (**C**, **E**, and **F**) or 2-tailed Mann-Whitney *U* test (**A**); ^#^*P* < 0.05, by 2-tailed Mann-Whitney *U* test (**E**).

Our analysis of publicly available RNA-Seq data (41) and our qPCR analyses of liver core biopsies revealed that the expression of *NOX4* and antioxidant genes tended to decline in humans with NASH and advanced fibrosis when compared with those with NAFL (Figure 1A). We reasoned that the decline in *NOX4* and antioxidant defense may exacerbate disease and contribute to NASH and fibrosis. Accordingly, we first asked whether the reduced expression of *NOX4* and antioxidant defense genes might have been hepatocyte intrinsic or otherwise indicative of changes in the abundance and activation of nonparenchymal cells in NASH and fibrosis. To this end, we isolated hepatocytes from C57BL/6 mice that were fed either a standard chow-diet, a HFD to promote obesity, insulin resistance, and steatosis, or a choline-deficient HFD (CD-HFD) to promote obesity, insulin resistance, and progression to NASH and fibrosis. CD-HFD–fed mice exhibited NASH with steatosis, lymphocytic infiltrates, and fibrosis (Figure 14, A and B). As noted already, *Nox4*, *Sod2*, *Cat*, and *Nqo1* mRNA levels were increased in lipid-laden hepatocytes from HFD-fed C57BL/6 mice (Figure 14C and Supplemental Figure 11). Importantly, as noted in humans with NASH and advanced fibrosis, the expression of *Nox4*, *Sod2*, *Cat*, and *Nqo1* was reduced in hepatocytes from CD-HFD fed mice (Figure 14C). The reduced expression of *Nox4*, *Sod2*, *Cat*, and *Nqo1* was accompanied by decreased NOX4, NQO1, SOD2, and catalase protein levels (Figure 14D) and reduced H_2_O_2_ production (Figure 14E). Importantly, the reduced levels of NOX4 and H_2_O_2_ and the decreased expression of antioxidant defense proteins in hepatocytes from CD-HFD fed mice were in turn accompanied by increased oxidative protein damage (Figure 14F). To independently assess the extent to which NASH and fibrosis may affect *Nox4* expression in vivo, we took advantage of a publicly available cell type–resolved transcriptional data set (59) to assess the expression of *Nox4*, as well as the expression of antioxidant defense genes in hepatocyte nuclei from mice fed a low-fat control diet versus those given a HFD rich in fructose, PA, and cholesterol (HFD-FPC diet) for 20 weeks; the HFD-FPC diet promotes variable degrees of NASH and fibrosis and recapitulates key aspects of human NASH (59). We found that *Nox4*, *Sod1*, *Sod2*, *Cat*, *Nfe2l2*, and *Nqo1* mRNA levels were increased in hepatocytes from HFD-FPC mice with mild NASH with minimal inflammation and no appreciable fibrosis (Figure 14G), but tended to decline in hepatocytes from mice with advanced NASH with comparable levels of steatosis but more inflammatory foci and overt fibrosis (Figure 14G). Therefore, the decline in NOX4 and NFE2L2 antioxidant defense associated with more advanced NASH and fibrosis may contribute to disease progression. To test this, we sought to induce the overexpression of NOX4 and ascertain whether this might sustain the antioxidant defense response and temper NASH and fibrosis in mice fed a CD-HFD. We took advantage of AAVs to overexpress either GFP (AAV-TBG-EGFP) or murine NOX4 (AAV-TBG-m*Nox4*) in hepatocytes before feeding mice a CD-HFD for 12 weeks. Hepatic *Nox4* was overexpressed by 7.9-fold (Figure 15A), and this was accompanied by increased NOX4 protein (Figure 15B) and a greater than 2-fold increase in extracellular H_2_O_2_ in isolated hepatocytes (Figure 15C). The overexpression of NOX4 was accompanied by modestly decreased body (Figure 15D) and liver (Figure 15E) weights but no change in body composition (Figure 15F). The decreased liver weights were accompanied by decreased expression of lipogenic genes (Figure 15G) and decreased steatosis (Figure 15H). Moreover, overexpression of NOX4 increased the hepatic expression of select antioxidant defense genes (Figure 15I) and proteins, including NFE2L2, SOD2, and NQO1 (Figure 15J and Supplemental Figure 12) and decreased oxidative damage as assessed by measuring protein carbonylation in hepatocytes from the corresponding mice (Figure 15K). The decreased oxidative damage was accompanied by a trend toward decreased hepatic CD8^+^ T cell infiltrates including decreased activated and effector/memory CD8^+^ T cells and a significant decrease in the proportion of exhausted and autoaggressive CD8^+^ T cells (Figure 16A); innate immune cells remained unaltered (Figure 16A). In addition, CD-HFD–fed mice overexpressing NOX4 were characterized by a trend toward decreased hepatic inflammation, as demonstrated by a decrease in immune infiltrates (Figure 16B) and a decrease in the expression of proinflammatory genes (Figure 16C). This was accompanied by decreased fibrosis, as assessed histologically (Figure 16D), and by the decreased hepatic expression of fibrosis-related genes (Figure 16E) and the decreased hepatic hydroxyproline levels (Figure 16F). Therefore, forced NOX4 overexpression could mitigate the development of NASH and fibrosis otherwise associated with the decline in *Nox4* expression in mice fed a NASH- and fibrosis-promoting CD-HFD.

## Discussion

In this study, we report that the induction of the NFE2L2 antioxidant defense response in NAFL, orchestrated first by mitochondria- and then NOX4-derived ROS, prevents liver damage and the progression to NASH with fibrosis. Conversely, reduced NOX4 levels and antioxidant defense might be a key feature of the progression to NASH and fibrosis.

The dysregulation of redox balance and the resulting oxidative stress can affect the progression of many human diseases (60). In particular, the increased production of mitochondrial oxidants in metabolic tissues has been causally linked to the development of insulin resistance (10, 15–21, 23, 28, 61, 62), a key pathological feature of type 2 diabetes and a leading risk factor for NAFLD (1). Genetic and pharmacological studies in rodents also point toward mitochondrial oxidative distress facilitating the progression to NASH and fibrosis (9, 10, 14, 20–22). However, beyond the potential to drive disease progression, ROS also have important physiological functions (60). For example, ROS such as H_2_O_2_ can function as second messengers to facilitate signaling in response to physiological stimuli by oxidizing and inactivating protein tyrosine phosphatases (63). Moreover, we have shown previously that H_2_O_2_ generated by NOX4 in skeletal muscle in mice during exercise can activate NFE2L2 and elicit adaptive responses that enhance muscle function and maintain insulin sensitivity (44). Similarly, others have reported that NOX4 also activates NFE2L2 in the heart to prevent oxidative stress and maintain cardiac function during exercise (64), whereas studies in humans have shown that antioxidants can negate the beneficial effects of exercise on insulin sensitivity and that this is accompanied by the decreased expression of muscle genes encoding mediators of antioxidant defense, including SOD1/-2 and GPX1 (65). Therefore, beyond their direct effect on signaling and physiological processes, ROS also drive adaptive responses that mitigate disease.

In this study, we have shown that mitochondria- and NOX4-derived ROS functioned in concert to elicit adaptive responses in the liver to temper NAFLD pathogenesis. We have shown that mitochondria- and NOX4-derived ROS drive NFE2L2 antioxidant defense in hepatocytes to limit the oxidative damage of macromolecules, the development of insulin resistance, and the progression to NASH and fibrosis. Our studies indicate that this response was graded, with mitochondrial ROS first stabilizing NFE2L2 to increase the expression of NOX4 and then further increasing ROS/H_2_O_2_ and the abundance of NFE2L2, thereby driving a robust antioxidant defense response. The importance of mitochondria-derived ROS/H_2_O_2_ in driving such adaptive/protective responses in the liver has been noted previously. Carter et al. reported that the beneficial effects of static magnetic and electric fields on insulin sensitivity were reliant on mitochondrial-derived O_2_•^–^ (66). Our own studies have shown that the deletion of *Gpx1* in hepatocytes and the resultant increased H_2_O_2_ levels enhanced insulin sensitivity in HFD-fed mice and attenuated NASH and fibrosis in mice fed a choline-deficient, amino acid–defined (CDAA) diet (67). In this study, we report that ROS generation by NOX4 was essential for the mitochondria-orchestrated adaptive response in NAFLD. The deletion of *Nox4* increased KEAP1 and decreased NFE2L2 protein levels, thereby reducing the expression of NFE2L2 target genes, including those encoding SOD2 and catalase. Previous studies have shown that the heterozygous deletion of *Sod2* in mice can promote mitochondrial oxidative stress (16), whereas genetic polymorphisms in *SOD2* in humans that decrease SOD2 mitochondria targeting and activity may be associated with NASH and the severity of fibrosis (22, 68). Thus, we propose that the reductions in SOD2 and catalase are key contributors to the oxidative distress evident in NOX4-deficient hepatocytes. The promotion of mitochondrial oxidative stress associated with the deletion/inhibition of NOX4 in hepatocytes diminished insulin signaling, whereas NOX4 deficiency in vivo promoted hepatic and systemic insulin resistance, a key driver of NAFLD. The deletion of *Nox4* and the diminished antioxidant defense also exacerbated the ability of mitochondrial oxidants to promote hepatocyte death. The progression to NASH and fibrosis in HFD-fed *Alb*-Cre *Nox4^fl/fl^* mice was accompanied by hepatocyte death, inflammation, and the recruitment and accumulation of CD8^+^ T cell subsets that have been causally linked to NASH (32, 54–57). The extent to which the progression to NASH and fibrosis may have been ascribable to oxidant-dependent cell death and ensuing inflammation, or otherwise to the oxidative inactivation of tyrosine phosphatases and the promotion of STAT1 signaling in hepatocytes to drive T cell recruitment, inflammation, and cell death, as we noted previously (32), remains unclear. Since both DNA damage and heightened hepatocyte STAT1 signaling were readily evident, we propose that both mechanisms are likely contributors. Interestingly, the deletion of *Nox4* in hepatocytes also increased DIO and enhanced steatosis, which is a risk factor for the progression to NASH. Although the molecular basis for the increased body weights of HFD-fed *Alb*-Cre *Nox4^fl/fl^* mice remains unclear, food consumption was increased, pointing toward potential crosstalk with the CNS and the control of feeding. The increased body weight/adiposity and systemic insulin resistance in HFD-fed *Alb*-Cre *Nox4*^fl/fl^ mice may have contributed to the increased steatosis by promoting the flux of lipids from adipose tissue. However, the increased steatosis was also accompanied by increased de novo lipogenesis. Previous studies have shown that NFE2L2 can negatively regulate the expression of lipid synthesis genes and protect from steatosis (35, 36, 51–53). Consistent with this, we found that the increase in lipogenic gene expression in hepatocytes resulting from the deletion of *Nox4* was corrected by administration of the NFE2L2 agonist sulforaphane. However, neither obesity per se nor steatosis is sufficient to drive the progression from NAFL to NASH in C57BL/6 mice or, indeed, humans (1, 7, 8). Therefore, although the increased weight gain and steatosis may have influenced disease severity, they would not have instigated disease progression.

Our findings seemingly contrast with those of previous studies reporting that the deletion of *Nox4* in hepatocytes attenuates NASH and fibrosis in mice fed diets that promote NASH and fibrosis, with or without obesity (24). Yet other studies have shown that systemic NOX1/4 inhibition with GKT137831 can repress liver fibrosis induced by bile duct ligation or CCl_4_ treatment (46, 69). Our studies do not preclude the possibility that NOX4-derived ROS in the context of diminished antioxidant defense contributes to oxidative distress and NAFLD pathogenesis. Moreover, our findings do not preclude a role for NOX1/4 in other cell types, including hepatic stellate cells, where NOX1/4 activation may drive an activated/fibrogenic state (70). Our studies demonstrate that, whereas steatosis and resultant increases in mitochondrial ROS drove *NOX4* expression in NAFL, the progression toward more advanced disease with fibrosis was accompanied by reduced *NOX4* and concomitantly reduced antioxidant defense. This is consistent with other studies that have shown that catalase activity declines in patients with overt NASH and fibrosis (10), as well as studies showing that hepatic *Nox4* and *Cat* transcripts are reduced in mice fed a NASH and fibrosis-promoting methionine- and choline-deficient diet (71). Importantly, we found that NOX4 overexpression in hepatocytes was able to increase antioxidant defense and decrease steatosis, inflammation, and fibrosis in mice fed a NASH- and fibrosis-promoting CD-HFD. Precisely why NOX4 and the antioxidant defense response may decline in NASH and fibrosis remains unclear, but may relate to the mitochondrial dysfunction associated with advanced disease (9, 10, 72). Nonetheless, in the context of reduced NOX4 and a resultant decreased adaptive antioxidant defense, any ROS generated by NOX4 might contribute to oxidative distress and exacerbate disease severity, such that its complete deletion or inhibition would be beneficial. This is line with a contribution of NOX4 to both adaptive redox signaling to mitigate NAFLD progression as well to oxidative distress to promote NASH pathogenesis.

The results of this study underscore the importance of redox balance in governing the transition from NAFL to NASH and the progressive development of fibrosis in obesity. Our studies define the interplay between mitochondria and NOX4 in eliciting optimal NFE2L2-dependent antioxidant defense in hepatocytes and point toward perturbations in this adaptive response being an important contributor to NAFLD pathogenesis.

## Methods

The experimental procedures and reagents used in this study can be found in the Supplemental Methods.

### Statistics.

Statistical significance was set at a *P* value of less than 0.05 and was determined with a 2-tailed Student’s *t* test, a Mann-Whitney *U* test, or a 1- or 2-way ANOVA with multiple comparisons. The data are presented as the mean ± SEM.

### Study approval.

Animal experiments were approved by the Monash University School of Biomedical Sciences Animal Ethics Committee (project IDs: 22138, 23077, 36631, 17687, 14368). The use of human tissue was approved by the Monash University Human Research Ethics Committee (CF12/2339-2012001246; CF15/3041-2015001282). Participants gave written consent before entering the study. Liver core biopsies were from men and women with obesity undergoing bariatric surgery (32) and were processed for RNA isolation. Sex difference analyses were not performed because of the low number of suitable donors.

### Data availability.

All data are available from the corresponding author and are provided in the Supplemental Supporting Data Values file.

## Author contributions

TT conceived of and conceptualized the study, designed experiments, wrote the manuscript, and interpreted the data with intellectual input from all authors. SG, SK, and CEX conceptualized and designed experiments, conducted and analyzed experiments, and contributed to the review and editing of the manuscript. AJG, AR, CAM, FW, JS, MJW, MT, PKG, WAB, and YYJ performed and/or analyzed experiments and/or contributed to the review and editing of the manuscript.

## Supplementary Material

Supplemental data

Unedited blot and gel images

Supporting data values

## Version 1 12/07/2023

In-Press Preview

## Version 2 02/01/2024

Electronic publication
