# Supplementary material for: Mitochondria- and NOX4-dependent antioxidant defense mitigates progression to nonalcoholic steatohepatitis in obesity
Source: J Clin Invest. 2024 Feb 1;134(3):e162533. doi: 10.1172/JCI162533 (PMC10849767; doi:10.1172/JCI162533)
Supplement: Supplemental data [file jci-134-162533-s017.pdf]

SUPPLEMENTAL DATA

Mitochondrial- and NOX4-dependent antioxidant defense mitigates progression to non-alcoholic steatohepatitis in obesity

Spencer Greatorex, Supreet Kaur, Chrysovalantou E. Xirouchaki, Pei K., Goh, Florian Wiede, Amanda Genders, Melanie Tran, YaoYao Jia, Arthe Raajendiran, Wendy A. Brown, Catriona A. McLean, Junichi Sadoshima, Matthew J. Watt and Tony Tiganis

Table of contents

Supplementary Materials and Methods.

Supplementary Table 1. Patient Characteristics – Related to Fig. 1.

Supplementary Figure 1. Antioxidant defense in NAFLD - Related to Fig. 1.

Supplementary Figure 2. Palmitate treatment increases ROS and the expression of lipogenesis and antioxidant defense genes - Related to Fig. 2.

Supplementary Figure 3. Diminished antioxidant defense in NOX4-deficient hepatocytes - Related to Fig. 3.

Supplementary Figure 4. Oxidative stress in NOX4-deficient hepatocytes - Related to Fig. 5 and 7.

Supplementary Figure 5. Unaltered body weight and adiposity in chow fed Alb-Cre;Nox4$^{fl/fl}$ mice - Related to Fig. 8.

Supplementary Figure 6. Hepatic Nox4 deletion promotes obesity, steatosis, and insulin resistance in female mice - Related to Fig. 8.

Supplementary Figure 7. Hepatic Nox4 deletion increases food intake, energy expenditure and EGP in male mice - Related to Fig. 8.

Supplementary Figure 8. Hepatic Nox4 deletion increases energy expenditure but does not alter RER in male mice - Related to Fig. 8.

Supplementary Figure 9. Hepatocyte NOX4 deletion or inhibition increases lipogenic gene expression - Related to Fig. 8 and Fig. 13.

Supplementary Figure 10. Gating strategy for the analysis of hepatic lymphocytes - Related to Fig. 10.

Supplementary Figure 11. Brightfield images of hepatocytes from mice fed chow, HFD and CD-HFD - Related to Fig. 14.

Supplementary Figure 12. Hepatocyte NOX4 overexpression increases antioxidant defense protein expression - Related to Fig. 15.

SUPPEMENTARY MATERIALS AND METHODS

Antibodies and reagents

Rabbit α-Phospho-AKT-S473 (p-AKT; Cat #4058, RRID:AB_331168), α-cleaved PARP (Asp214; Cat #9544, RRID:AB_2160724), α-Phospho-p38 MAPK (Cat #9219, RRID:AB_331294), α-cleaved caspase-3 (Asp175; Cat #9664, RRID:AB_2070042), α-Phospho-STAT1 (Y701) (p-STAT1; Cat #9167, RRID:AB_561284) , α-Phospho-Histone H2A.X-Ser139 (to detect γH2AX; Cat #9718, RRID:AB_2118009), α-Phospho-SAPK/JNK (Thr183/Tyr185) (Cat #4668, RRID:AB_823588), α-PRDX1 (Cat #8499, RRID:AB_10950824), α-SCD1 (Cat #2794, RRID:AB_2183099) and α-FASN (Cat #3180, RRID:AB_2100796) and mouse α-AKT (pan) (Cat #2920, RRID:AB_1147620) were from Cell Signaling Technology (Beverly, MA). Rabbit α-catalase (Cat #ab1877, RRID:AB_302649), α-PRDX3 (Cat #sc-130336, RRID:AB_2188475), α-NFE2L2 (Cat #sc-365949, RRID:AB_10917561) and α-NOX4 (Cat #sc-30141, RRID_AB_2151703, discontinued antibody) and mouse α-PRDX2 (Cat #sc-59658, RRID:AB_1128598) and α-SOD2 (Cat #sc-133134, RRID:AB_2191814) were from Santa Cruz Biotechnology (Santa Cruz, CA). Mouse α-tubulin (Cat #T5168, RRID:AB_477579) and α-vinculin (Cat #V9131, RRID:AB_4776298) and rabbit α-NFE2L2 (Cat #AV38745, RRID:AB_1854419) were from Sigma-Aldrich (St Louis, MO). Rabbit α-4-Hydroxynonenal (4-HNE) (Cat #ab46545, RRID:AB_722490), α-KEAP1 (Cat #139729, RRID:AB_2891077), α-CD3 (SP7; Cat #ab16669, RRID:AB_443425), goat α-NCF1/p47^{phox} (Cat #ab795, RRID:AB_306163) and α-catalase (Cat 1877, RRID:AB_302649) were from Abcam (San Francisco, CA). Mouse α-gp91^{phox} (Cat #611415, RRID:AB_398937) and α-RAC1 (Cat #610651, RRID:AB_397978) were from BD Bioscience (San Jose, CA), mouse α-GAPDH (Cat #AM4300,

RRID:AB_2536381) from Thermo Fisher Scientific (Waltham, MA), mouse α-NQO1 (Cat #NB200-209, RRID:AB_10002706) from Novus Biologicals (Littleton, CO) and sheep α-NQO1 from R&D Systems (Minneapolis, MN). Sodium palmitate and bovine and human insulin were from Sigma-Aldrich (St Louis, MO), fatty acid (FA)-free BSA from Bovogen Biologicals (Australia), recombinant murine EGF from PeproTech (Rocky Hill, NJ) and AAV-TBG-EGFP (serotype 8; VB1743), AAV-TBG-iCre (serotype 8; VB1724) or AAV8-TBG-m*Nox4* (NM_015760.5; serotype 8; VB220713-1312mkr) from Vector Biolabs (Malvern, PA). DeadEnd Colorimetric TUNEL System (Cat #G7130) was from Promega (Madison, WI).

Mice

Mice were maintained on a 12 h light-dark cycle in a temperature-controlled pathogen-free facility (Monash ARL) with *ad libitum* food and water. Age- and sex-matched mice were used for experiments. *Nox4*$^{fl/fl}$ (C57BL/6) and *Alb*-Cre;*Gpx1*$^{fl/fl}$ (C57BL/6) mice have been described previously (1, 2). *Nox4*$^{fl/fl}$ mice were mated with *Alb*-Cre (C57BL/6) mice (JAX) to generate *Alb*-Cre;*Nox4*$^{fl/fl}$ (C57BL/6) mice. Where indicated *Nox4*$^{fl/fl}$ mice were injected with AAV-TBG-EGFP (serotype 8; Vector Biolabs, Malvern, PA) as a control, or AAV-TBG-iCre (serotype 8; Vector Biolabs, Malvern, PA) to delete *Nox4* in hepatocytes in adult mice. Alternatively, C57BL/6J male mice were injected with AAV-TBG-EGFP as a control, or AAV-TBG-m*Nox4* (NM_015760.5; serotype 8; Vector Biolabs, Malvern, PA) to overexpress *Nox4* in hepatocytes. C57BL/6J male mice were purchased from the Monash Animal Research Platform or the WEHI Animal Facility (Kew, Australia). Mice were fed a breeding diet (8.5% fat; Barastoc, Ridley AgriProducts, Australia), or where indicated a standard chow diet (20% protein, 4.8% fat, and 4.8% crude fiber; Specialty Feeds), a HFD [18.4% protein, 23.5% fat (46% energy from fat), 4.7% fibre; Specialty Feeds #SF04-027), or a CD-HFD [no added choline chloride, 22.6% protein, 23.5% fat (43% energy from fat), 5.4% fibre; Specialty Feeds

#SF15-078]. Animal experiments were approved by the Monash University School of Biomedical Sciences Animal Ethics Committee (Project IDs: 22138, 23077, 36631, 17687, 14368) and performed in accordance with the NHMRC Australian Code of Practice for the Care and Use of Animals.

Hepatocytes

Mouse primary hepatocytes were isolated by the two-step collagenase perfusion method as described previously (3). Briefly, mice were anaesthetised (50 mg/kg pentobarbitone intraperitoneal), the hepatic portal vein cannulated with a 24G catheter (BD Insyte-N Autogard Shielded IV Catheter, BD Biosciences) and after cutting the inferior vena cava perfused with wash buffer [Hank's Balanced Salt Solution (HBSS) without $Ca2^{+}$, $Mg2^{+}$ and Phenol Red (Gibco Life Technologies, Carlsbad, CA) containing 1 mM EDTA and 25 mM HEPES pH 7.4] and then with perfusion buffer [HBSS with $Ca2^{+}$, Mg2+ and Phenol Red (Gibco Life Technologies, Carlsbad, CA), 25 mM HEPES pH 7.4 and 5 μg/mL Liberase™ (Roche, Germany)] for 5-10 min (50-100 ml). Perfused livers from adult male mice were dissected and minced with surgical scissors, washed 3-4 times in ice-cold HBSS with $Ca2^{+}$, Mg2+ and Phenol Red and cultured on 30 μg/ml entactin-collagen-laminin cell attachment matrix (Merck, Millipore, CA)-coated 6-well tissue culture dishes in M199 media (Sigma Aldrich St. Louis, MO) supplemented with 2 mM glutamine, 10% (v/v) heat inactivated FBS, 100 U/ml penicillin/streptomycin, 10 ng/ml EGF at 37°C, 5% CO_2, 5%O_2 (HERACELL VIOS 160i, CO_2 incubator, Thermo Fisher Scientific, Waltham, MA) Unless otherwise indicated, hepatocytes were cultured for 16 h and then processed for RNA isolation or immunoblotting.

Hepatocytes were treated with vehicle control (DMSO), 1 μM sulforaphane (Sigma Aldrich, St. Louis, MO), 20 μM Mito-TEMPO (Sigma Aldrich, St. Louis, MO), 40 μM GKT137831 (Sigma Aldrich, St. Louis, MO) or 3 μM MG132 (Sigma Aldrich, St. Louis, MO)

for 16 h when assessing effects on gene expression, or 36 h when assessing effects on protein levels or signalling. For insulin stimulations, hepatocytes were serum-starved in M199 supplemented with 0.1% (v/v) FBS for 6 h and then stimulated with 1 nM bovine insulin for the indicated times. Where indicated hepatocytes were pre-treated with vehicle control, sulforaphane or Mito-TEMPO for 16 h. For assessing sensitivity to oxidative stress, hepatocytes from high fat fed mice were treated with vehicle control (EtOH), 2.5 μM menadione (Sigma Aldrich, St. Louis, MO) or varied dilutions of menadione for 24 h for immunoblotting and 48 h for analysis of cell survival. Hepatocyte cell death was assessed in 96 well plates (10,000 cells plated/well) using the MTT assay (Roche, Germany) according to the manufacturer's instructions and absorbance measured using a BMG Labtech Clariostar plus plate reader (Hamilton, Reno, NV) at 595 nm. Where indicated menadione treated hepatocytes were pre-treated with sulforaphane or Mito-TEMPO for 16 h. For assessing responses to fatty acids (FAs), hepatocytes from chow-fed mice were treated with vehicle [FA-Free-BSA 0.12% (w/v)] or palmitate (PA)-conjugated BSA (PA-BSA; equivalent to 200 μM of free-FA) prepared freshly as described previously (3) for 16 h for mRNA analyses or 36 h for immunoblotting. Where indicated PA-BSA treated cells were pre-treated with Mito-TEMPO or GKT137831 for 16 h.

Hepatocyte-specific Nox4 deletion or overexpression in adult mice

To delete *Nox4* in hepatocytes in obese mice, 7-week-old *Nox4*$^{fl/fl}$ male mice were HFD-fed for 10 weeks to render them obese and promote steatosis and then injected via the tail vein with 2×10^{11} genome copies/body weight AAV-TBG-EGFP (serotype 8), or AAV-TBG-iCre (serotype 8) and high fat feeding continued for a further 10 weeks. To overexpress murine *Nox4* in hepatocytes, 8-week-old C57BL/6 male mice were injected via the tail vein with 2 ×

10^{11} genome copies/body weight AAV-TBG-EGFP (serotype 8) as a control, or AAV-TBG-m*Nox4* (serotype 8) and mice then fed a CD-HFD for up to 12 weeks.

Metabolic and blood measurements

Mouse body weights were monitored weekly and body composition was assessed by EchoMRI (Echo Medical Systems, Houston, TX) or dual energy X-ray absorptiometry (DEXA, Lunar PIXImus2, GE Healthcare). Insulin tolerance tests (ITTs) and glucose tolerance tests (GTTs) were performed as described previously (4). Blood was collected from conscious mice by submandibular bleeding and plasma insulin levels determined using the mouse insulin ELISA (ALPCO, Salem, NH) or an in-house ELISA (Monash Antibody Technologies Facility) and the corresponding blood glucose levels determined with an Accu-Check glucometer. For an assessment of hepatic insulin signaling, mice were fasted for 4 h, injected intraperitoneally with 0.65 mU/g human insulin and after 10 min the livers excised and frozen in liquid N_2 for subsequent analyses.

Food intake, VO_2, respiratory exchange ratios (RERs) and energy expenditure were assessed over 72 h after 24 h acclimation using a Promethion Metabolic Screening System (Sable Systems International, North Las Vegas, NV) fitted with indirect open circuit calorimetry, running wheels and food consumption and activity monitors.

Hyperinsulinaemic-euglycaemic clamps

Hyperinsulinaemic-euglycaemic clamps were performed as described previously (5). Briefly, mice were anesthetized under 2% (v/v) isoflurane (250 ml/min O_2) and the left common carotid artery and the right jugular vein catheterized for sampling and infusions. On the day of the clamp food was removed and after 3.5 h fasting, a primed (2 min, 0.5 μCi/min) continuous infusion (0.05 μCi/min) of [3-^{3}H]-glucose was administered to measure basal

glucose turnover. After 5 h fasting, mice received a continuous insulin infusion (4 mU/kg/min), and blood glucose was maintained at basal levels (euglycaemia) by a variable infusion of a 50% (w/v) glucose solution. Arterial blood samples were collected at basal and during steady state conditions. Mice were then euthanised and tissues extracted and frozen for subsequent gene expression.

Biochemical analyses

Livers were dissected and snap-frozen in liquid N_2. Livers were homogenised mechanically or by using disposable tissue grinders (Fisherbrand™ Pellet Pestles™, Thermo Fisher Scientific, Waltham, MA) for 30-60 sec on ice in 10 volumes of ice-cold RIPA lysis buffer [50mM HEPES (pH 7.4), 1% (w/v) Triton X-100, 1% (v/v) sodium deoxycholate, 0.1% (w/v) SDS, 150 mM NaCl, 10% (v/v) glycerol, 1.5 mM $MgCl_2$, 1 mM EDTA, 50 mM NaF, 1 µg/ml pepstatin A , 1 mM benzamidine, 2 mM phenylmethylsulphonyl fluoride, 10 mM beta-glycerophosphate, and 1 mM sodium vanadate], incubated on ice for 30 min and clarified by centrifugation (16,000 x g for 30 min, 4°C). Hepatocytes in 6-well tissue culture plates were washed twice with ice-cold PBS and then lysed with 100 µl of ice-cold RIPA lysis buffer/well, incubated on ice for 30 min and clarified by centrifugation (16,000 x g for 30 min, 4°C). Proteins were resolved by SDS-PAGE (10%) and transferred to PVDF and immunoblotted as described previously (6).

Carbonylated proteins were assessed by immunoblotting using the OxyBlot™ Protein Oxidation Detection Kit (Merck Millipore, Burlington, MA) according to the manufacturer's instructions.

Triglycerides, diglycerides and ceramide were extracted and quantified as described previously (4). Hepatic *de novo* lipogenesis was assessed by measuring the incorporation of ^{14}C-glucose (Glucose, D-[^{14}C(U)]-, 100 µCi/ml, Perkin Elmer) into triglycerides in liver slices

ex vivo as described previously (4, 7). Fatty acid oxidation was assessed my measuring the oxidation of exogenous [1-^{14}C]palmitate in liver slices as described previously (4).

Liver hydroxyproline levels were determined using the colorimetric Hydroxyproline Assay Kit (ab222941) from Abcam (San Francisco, CA) according to the manufacturer's instructions.

ROS measurements

H_2O_2 released by live hepatocytes was measured using Amplex® Red hydrogen peroxide assay kit (Invitrogen, Carlsbad, CA). Hepatocytes were seeded overnight on a Perkin-Elmer 96-well View Plate (Waltham, MA), serum-starved in M199 supplemented with 0.1 (v/v) FBS for 4 h and then incubated in PBS containing 200 µM Amplex® Red reagent and 0.5 U/ml HRP. Fluorescence measured over 45 min on a BMG Labtech Clariostar plus plate reader (Hamilton, Reno, NV) at an excitation of 545 nm and emission of 590 nm and fluorescence within the linear range recorded and normalised to total protein measured using the Bradford assay.

Mitochondrial superoxide production was monitored using MitoSOX™ Red (Invitrogen, Carlsbad, CA), a live-cell permeable dye that is targeted to the mitochondria and fluoresces red after oxidation by superoxide. Hepatocytes (25,000 cells/well) were grown on 8 well Nunc® Lab-Tek® II Chamber Slides ™ (Sigma-Aldrich, St Louis, MO) in M199 medium supplemented with 2 mM glutamine, 10% (v/v) heat inactivated FBS, 100 U/ml penicillin/streptomycin, 10 ng/ml EGF at 37°C, 5% CO_2, 5% O_2 for 16 h. Hepatocytes were then incubated with 2. 5 µM MitoSOX™ Red in HBSS at 37°C for 10 min and then washed three times with HBSS, counter-stained with DAPI 50 ng/ml in HBSS and fixed in 4% (w/v) paraformaldehyde (Thermo Fisher Scientific, Waltham, MA) for 20 min at 4°C. MitoSOX™ Red fluorescence was visualized by confocal microscopy (Leica SP8 confocal microscope,

Leica MICROSYSTEMS, Wetzlar, Germany) at an excitation of 510 nM and an emission of 580 nM.

Histology

Livers were fixed overnight in a neutral-buffered formalin solution and then transferred to 70% (v/v) ethanol. Tissue processing and paraffin embedding were performed by the Monash Histology Platform (Monash University, Clayton Campus, Australia) and sections were stained with Hematoxylin-Eosin or PicroSirius Red. Alternatively, livers were frozen in OCT and stained with Oil red-O. Slides were scanned using an Aperio Scanscope AT Turbo (Aperio, Vista, CA) and analysed using OlyVIA imaging software (Olympus, Tokyo, Japan).

Immunohistochemistry

Sections from formalin-fixed and paraffin-embedded liver blocks were dewaxed in Histopure (CSA Pathology, Australia) for 3 x 5 min, dehydrated in 100% ethanol for 3 x 5 min and antigen-retrieved in Tris/EDTA pH 8.0 buffer for 5 min in a pressure cooker (70 kpa). Endogenous peroxidase activity was blocked in 3% (v/v) H_2O_2 for 10 min and sections were blocked with 3% (v/v) normal goat serum in phosphate-buffered saline (PBS) for 1 h at room temperature and incubated overnight at 4^oC with primary antibodies to CD3, p-STAT-1 or γH2AX. Positive cells were visualised using rabbit IgG VECTORSTAIN ABC Elite and DAB (3,3'-diaminobenzidine) Peroxidase Substrate Kits (Vector Laboratories, UK) and nuclei were counterstained with haematoxylin. TUNEL staining was performed following the manufacturer's instructions (Promega, Madison, WI) with some minor changes. Briefly, sections were dewaxed and dehydrated, blocked in 3% H_2O_2 for 10 min, treated with 20 μg/ml Proteinase K for 5 min, blocked in 3% (w/v) BSA in PBS for 1 h at room temperature and incubated for 1 h with TdT reaction mix at 37°C. Apoptotic cells were visualised using

Streptavidin-HRP and DAB Substrate and nuclei were counterstained with hematoxylin. Sections were visualised on an Olympus CX33 microscope (Olympus) and imaged at 10x or 20x magnification.

Flow cytometry

Hepatic lymphocytes were isolated from livers strained through a 200 µm metal sieve using a 33% Percoll (GE Healthcare, Chicago, IL) gradient. Contaminating red blood cells were removed with Red Blood Cell Lysing Buffer Hybri-Max™ (Sigma-Aldrich, St Louis, MO). Lymphocytes were stained with the specified antibodies on ice for 30 min in PBS/2% FBS and analyzed using a FACSymphony A3 (BD Biosciences, Franklin Lakes, NJ) and data was analysed using FlowJo10.8 (Tree Star Inc.) software. For cell quantification, a known number of Flow-Count Fluorospheres (Beckman Coulter) were added to samples before analysis. Dead cells were excluded with propidium iodide (1 µg/ml; Sigma-Aldrich, St Louis, MO) or LIVE/DEAD® Fixable Near IR-stain (ThermoFisher Scientific, Waltham MA). For the detection of intracellular FoxP3 the Foxp3/Transcription Factor Staining Buffer Set (eBioscience, San Diego, CA) was used according to the manufacturer's instructions. The following antibodies from BD Biosciences (Franklin Lakes, NJ), BioLegend (San Diego, CA) or eBioscience (San Diego, CA) were used for flow cytometry: Brilliant™ Violet 605 (BV605)-conjugated TCR-β (H57-597); Phycoerythrin-cyanine 7 (PE-Cy7) or Peridinin-chlorophyll cyanine 5.5 (PerCP-Cy5.5)-conjugated CD4 (RM4-5); BV711 or Brilliant™ Ultraviolet 395 (BUV395)-conjugated CD8 (53-6.7); BV711-conjugated CD11b (M1/70); PE-conjugated CD25 (P61); BV786-conjugated CD44 (IM7); BUV737-conjugated CD62L (Mel-14); V450-conjugated CD69 (H1.2F3); Fluorescein isothiocyanate (FITC)-conjugated CD279 (PD-1, RMP1-14); BV711-conjugated CD366 (TIM-3, RMT3-23); Allophycocyanin (APC)-conjugated KLRG1 (2F1); PE-Dazzle™ 594-conjugated CXCR6 (SA051D1); FITC-

conjugated FoxP3 (FKJ-16s); V421-conjugated NK1.1 (PK136) and PE-conjugated F4/80 (BM8). PE-Cy7-conjugated Streptavidin was used to detect biotinylated CD49d (9C10).

Quantitative PCR

Liver RNA (medial lobe) was extracted using RNAzol (Sigma-Aldrich, St Louis, MO) and total RNA quality and quantity determined using a NanoDrop 1000 (Thermo Fisher Scientific, Waltham MA). mRNA was reverse transcribed using the High-Capacity cDNA Reverse Transcription Kit (Applied Biosystems, Foster City, CA) and processed for quantitative real-time PCR using Quantinova SYBR Green Master Mix (Qiagen, Hiden, Germany) and Biorad Prime PCR primers. *Gapdh* was used as an internal control/reference gene. The real-time PCR primers included SYBR gene expression assays (Biorad, CA) for mouse *Gapdh* (qMmuCED0027497), *Nox4* (qMmuCIP0035663), *Cat* (qMmuCIP0031341), *Nqo1* (qMmuCIP0033090), *Sod2* (qMmuCIP0006109), *Nfe2l2* (qMmuCIP0033901), *Sod1* (qMmuCIP0036673), *Pgd* (qMmuCIP0019196), *Me1* (qMmuCIP0011443), *G6pd* (qMmuCIP0035943), *Gclc* (qMmuCIP0032546), *Gclm* (qMmuCIP0017192), *Gss* (qMmuCIP0054780), Gsr (qMmuCID0005414), *Prdx1* (qMmuCIP0047858), *Prdx3* (qMmuCIP0054955), *Acta2 (qMmuCIP0032840), Col1a1 (*qMmuCID0021007*), Fn1 (*qMmuCID0019534*), Hmox1 (*qMmuCID0040051*), Cxcl9 (qMmuCID0023784), Tgfb1 (qMmuCEP0053152), Ifng (qMmuCID0006268), Gpx1 (qMmuCED0003929), Tnf (qMmuCED0004141)* or and for human *GAPDH* (qHsaCED0038674), *NOX4* (qHsaCID0012395), *SOD2* (qHsaCED0036418), *SOD1* (qHsaCID0008628) *NQO1* (qHsaCED0036869), *NFE2L2* (qHsaCED0038543) and *CAT* (qHsaCED0043914). Alternatively, cDNA was processed for quantitative real-time PCR using the TaqMan Universal PCR Master Mix and the following TaqMan Gene Expression probes for mouse (Applied Biosystems) *Catalase* (Mm00437992_m1), *Cybb* (Mm01287743_m1),

Gpx1(Mm00656767_g1), *Fasn* (Mm00662319_m1), *Gapdh* (Mm99999915_g1), *Ncf1* (Mm00447921_m1), *Nox4* (Mm00479246_m1), *Rac1* (Mm01201653_mH), *Sod1* (Mm01344233_g1), *Sod2* (Mm01313000_m1), *Scd1* (Mm00772290_m1), *Srebf1* (Mm00550338_m1).

Transcriptome analysis

Raw trimmed RNA-seq data corresponding to human livers (GSE130970) from lean healthy versus NAFLD patients (8) or hepatocyte nuclei (GSE162876) from the livers of HEP-INTACT mice fed a chow diet or a NASH promoting HFD rich in fructose, palmitate and cholesterol (9) was mapped to the human hg19 or mouse mm10 genomes respectively using STAR aligner (v2.4.2a). Raw BAM files were converted to sequencing reads per gene, with genes having less than an average of 10 reads across all samples were filtered out, then data was normalised to total read count using DEseq2 (v1.24.0). Samples were divided into groups according to the Kleiner classification.

Statistics

All data is represented as population mean ± standard error of the mean (SEM). Statistical significance was determined with a 2-tailed Student's t test or a 2-tailed Mann-Whitney U Test for groups consisting of two experimental groups, or a one-way or two-way ANOVA with multiple comparisons (Tukey or Sidak respectively) when comparing three or more experimental groups. The level of significance was set at $p < 0.05$: * or $^{\#}$ $p<0.05$, ** or $^{\#\#}$ $p<0.01$, *** $p<0.001$ and **** $p<0.0001$. Statistical details for individual experiments such as exact values of n can be found in figures and figure legends.

SUPPLEMENTARY REFERENCES:

1. Merry TL, Tran M, Dodd GT, Mangiafico SP, Wiede F, Kaur S, et al. Hepatocyte glutathione peroxidase-1 deficiency improves hepatic glucose metabolism and decreases steatohepatitis in mice. *Diabetologia.* 2016;59(12):2632-44.
2. Kuroda J, Ago T, Matsushima S, Zhai P, Schneider MD, and Sadoshima J. NADPH oxidase 4 (Nox4) is a major source of oxidative stress in the failing heart. *Proc Natl Acad Sci USA.* 2010;107(35):15565-70.
3. Gurzov EN, Tran M, Fernandez-Rojo MA, Merry TL, Zhang X, Xu Y, et al. Hepatic oxidative stress promotes insulin-STAT-5 signaling and obesity by inactivating protein tyrosine phosphatase N2. *Cell Metab.* 2014;20(Jul 1):85-102.
4. Loh K, Deng H, Fukushima A, Cai X, Boivin B, Galic S, et al. Reactive oxygen species enhance insulin sensitivity. *Cell Metab.* 2009;10(4):260-72.
5. Dodd GT, Lee-Young RS, Bruning JC, and Tiganis T. TCPTP Regulates Insulin Signaling in AgRP Neurons to Coordinate Glucose Metabolism With Feeding. *Diabetes.* 2018;67(7):1246-57.
6. Tiganis T, Bennett AM, Ravichandran KS, and Tonks NK. Epidermal growth factor receptor and the adaptor protein $p52^{Shc}$ are specific substrates of T-cell protein tyrosine phosphatase. *Mol Cell Biol.* 1998;18:1622-34.
7. Grohmann M, Wiede F, Dodd GT, Gurzov EN, Ooi GJ, Butt T, et al. Obesity Drives STAT-1-Dependent NASH and STAT-3-Dependent HCC. *Cell.* 2018;175(5):1289-306.
8. Hoang SA, Oseini A, Feaver RE, Cole BK, Asgharpour A, Vincent R, et al. Gene Expression Predicts Histological Severity and Reveals Distinct Molecular Profiles of Nonalcoholic Fatty Liver Disease. *Sci Rep.* 2019;9(1):12541.

9. Loft A, Alfaro AJ, Schmidt SF, Pedersen FB, Terkelsen MK, Puglia M, et al. Liver-fibrosis-activated transcriptional networks govern hepatocyte reprogramming and intra-hepatic communication. *Cell Metab.* 2021;33(8):1685-700.

Supplementary Table 1. Patient Characteristics – Related to Figure 1.

**Patient Characteristics**	**No steatosis**	**NAFL**	**NASH Mild Fibrosis**	**NASH Advanced Fibrosis**
	**Number of patients**			
**Total**	7	13	20	22
**Lobular Inflammation Score**				
0	5	2		0
1	2	10	17	16
2		1	3	6
**Ballooning Score**				
0	7	13		
1			8	9
2			12	13
**Steatosis Score**				
0	7			
1		6	6	9
2		4	7	9
3		3	7	4
**NAS**				
0	5			
1	2	1		
2		6		
3		3	3	3
4		3	5	6
5			8	9
6			4	4
**Fibrosis Score**				
0	7	13		
1			20	
2				7
3+				15

Liver histological assessment of core biopsies described previously (8) reanalysed for the expression of antioxidant defense genes (GSE130970). The NAFLD Activity Score (NAS), which is the unweighted sum of the steatosis (0-3), lobular inflammation (0-3) and hepatocellular ballooning (0-2) scores, and the fibrosis score are shown.

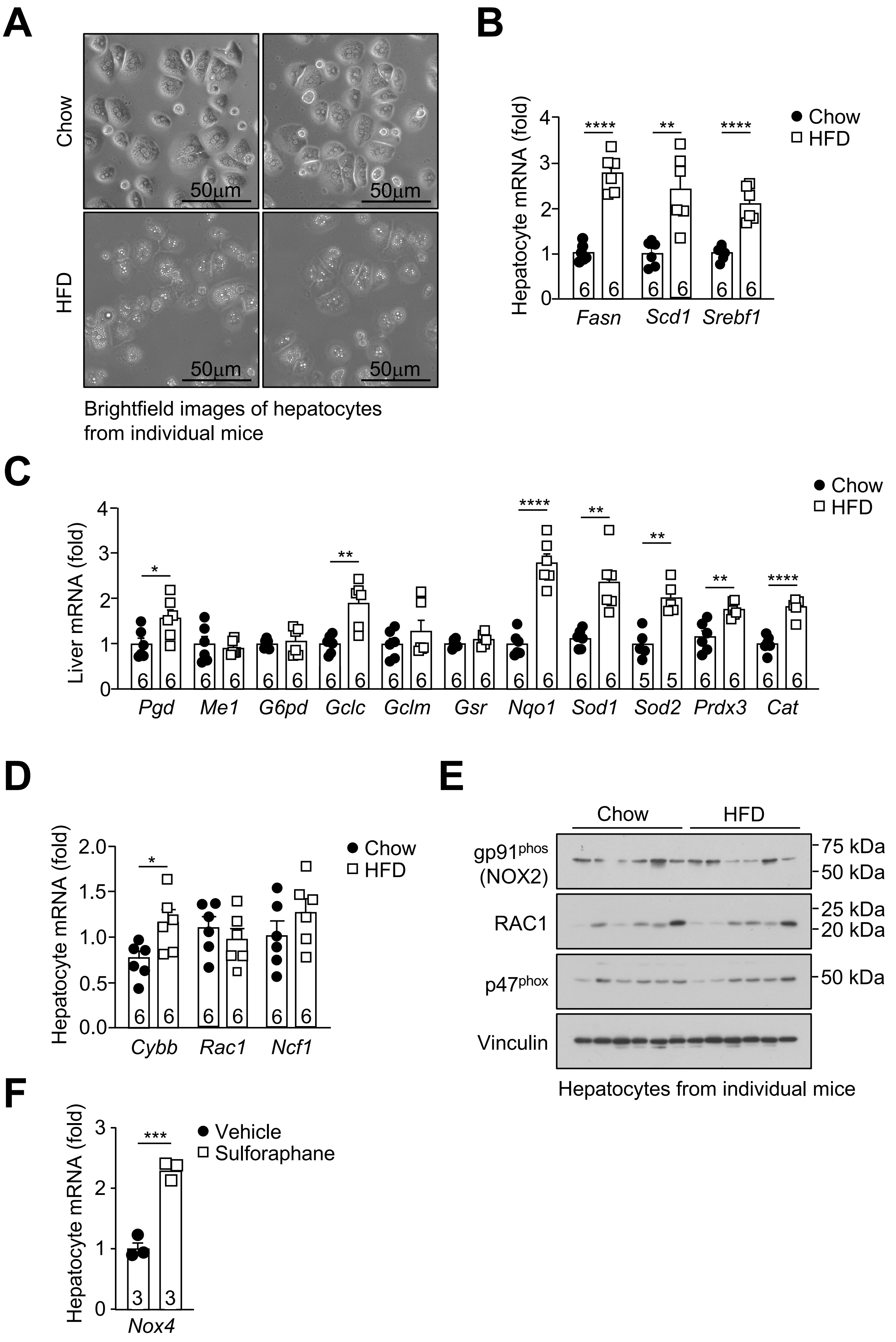
A
Chow
HFD
50μm
Brightfield images of hepatocytes from individual mice
B
Hepatocyte mRNA (fold)
Chow
HFD
Fasn
Scd1
Srebf1
C
Liver mRNA (fold)
Pgd
Me1
G6pd
Gclc
Gclm
Gsr
Nqo1
Sod1
Sod2
Prdx3
Cat
D
Cybb
Rac1
Ncf1
E
gp91phos (NOX2)
RAC1
p47phox
Vinculin
75 kDa
50 kDa
25 kDa
20 kDa
Hepatocytes from individual mice
F
Vehicle
Sulforaphane
Nox4

Supplementary Figure 1. Antioxidant defense in NAFLD. **a-c**) 8-week-old C57BL/6 mice were fed a chow diet (4.8% fat) or high fat diet (HFD; 23% fat) for 8-10 weeks and hepatocytes isolated by a two-step collagenase perfusion method. Hepatocytes were cultured for 16 h at 5% O_2 and processed for **a**) phase contrast microscopy, or **b-c**) qPCR. **d**) 8-week-old C57BL/6 mice were fed a chow diet or HFD for 12 weeks and livers (medial lobe) processed for qPCR. **e**) 8-week-old C57BL/6 mice were fed a chow diet or HFD for 8-10 weeks and hepatocytes isolated, cultured for 16 h at 5% O_2 and processed for immunoblotting. **f**) 8-week-old C57BL/6 mice were fed a chow diet (4.8% fat) and hepatocytes isolated by a two-step collagenase perfusion method. Hepatocytes were cultured for 16 h at 5% O_2 and then treated with the NFE2L2 agonist isothiocyanate sulforaphane (1 μM) for 48 h and processed for qPCR to assess *Nox4* expression. Representative and quantified results are shown (mean ± SEM) for the indicated number of mice. Significances determined using Student's t-tests.

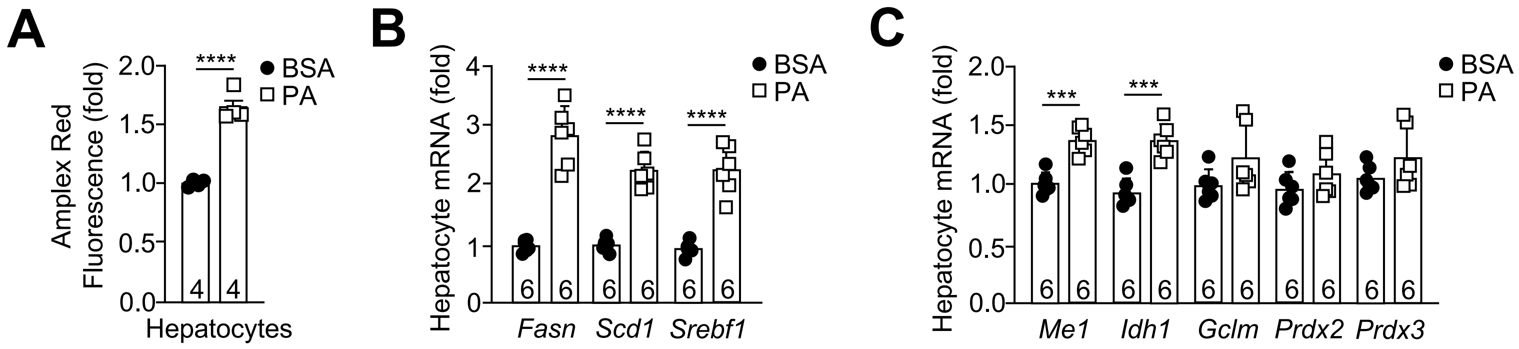

A
B
C
BSA
PA
Amplex Red Fluorescence (fold)
Hepatocytes
****
Hepatocyte mRNA (fold)
Fasn
Scd1
Srebf1
Me1
Idh1
Gclm
Prdx2
Prdx3
***

Supplementary Figure 2. Palmitate treatment increases ROS and the expression of lipogenesis and antioxidant defense genes. 8-week-old C57BL/6 mice were fed a standard chow diet (4.8% fat) for 8-10 weeks and hepatocytes isolated, cultured for 16 h at 5% O_2 and treated with either BSA-conjugated palmitate [(PA; equivalent to 200 μM free-fatty acid (FFA)] or the equivalent amount of BSA for 16 h and processed for **a**) H_2O_2 measurements using Amplex Red or **b-c**) qPCR. Representative and quantified results are shown (mean ± SEM) for the indicated number of mice. Significances determined using Student's t-tests.

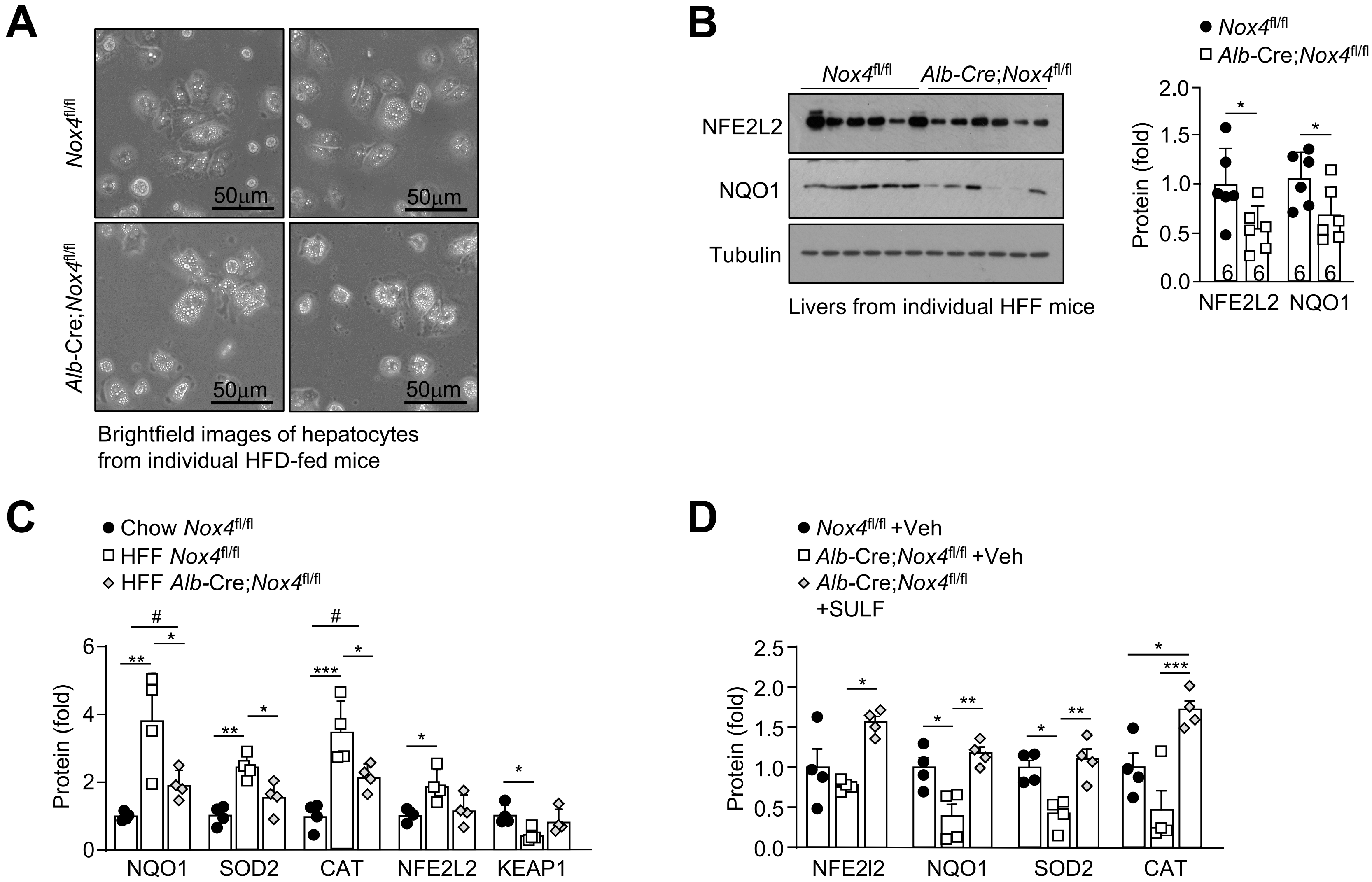
A
Nox4fl/fl
Alb-Cre;Nox4fl/fl
50µm
50µm
50µm
50µm
Brightfield images of hepatocytes
from individual HFD-fed mice
B
Nox4fl/fl
Alb-Cre;Nox4fl/fl
NFE2L2
NQO1
Tubulin
Livers from individual HFF mice
Nox4fl/fl
Alb-Cre;Nox4fl/fl
Protein (fold)
NFE2L2
NQO1
C
Chow Nox4fl/fl
HFF Nox4fl/fl
HFF Alb-Cre;Nox4fl/fl
Protein (fold)
NQO1
SOD2
CAT
NFE2L2
KEAP1
D
Nox4fl/fl +Veh
Alb-Cre;Nox4fl/fl +Veh
Alb-Cre;Nox4fl/fl +SULF
Protein (fold)
NFE2l2
NQO1
SOD2
CAT

Supplementary Figure 3. Diminished antioxidant defense in NOX4-deficient hepatocytes.
a) 8-week-old *Nox4*$^{fl/fl}$ or *Alb*-Cre;*Nox4*$^{fl/fl}$ (C57BL/6) mice were fed a HFD for 8-10 weeks and hepatocytes isolated by a two-step collagenase perfusion method. Hepatocytes were cultured for 16 h at 5% O_2 and processed phase contrast microscopy. **b**) 8-week-old *Nox4*$^{fl/fl}$ or *Alb*-Cre;*Nox4*$^{fl/fl}$ mice were fed a HFD for 12 weeks and livers (medial lobe) were extracted and processed for immunoblotting and quantification. **c**) 8-week-old *Nox4*$^{fl/fl}$ or *Alb*-Cre;*Nox4*$^{fl/fl}$ mice were fed a standard chow diet or HFD as indicated for 8-10 weeks and hepatocytes isolated, cultured for 16 h at 5% O_2 and processed for immunoblotting; antioxidant defense proteins were quantified (relates to Fig. 3k). **d**) 8-week-old *Nox4*$^{fl/fl}$ or *Alb*-Cre;*Nox4*$^{fl/fl}$ mice were fed a HFD for 8-10 weeks and hepatocytes isolated, cultured for 16 h at 5% O_2 and then treated with vehicle (DMSO) or 1 μM sulforaphane (SULF) for 36 h and processed for immunoblotting; antioxidant defense proteins were quantified (relates to Fig. 4b). Representative and quantified results are shown (means ± SEM) for the indicated number of mice. Significance determined using (b) Student's t-test or (c, d) a one-way ANOVA; # indicates significance using Student's t-tests.

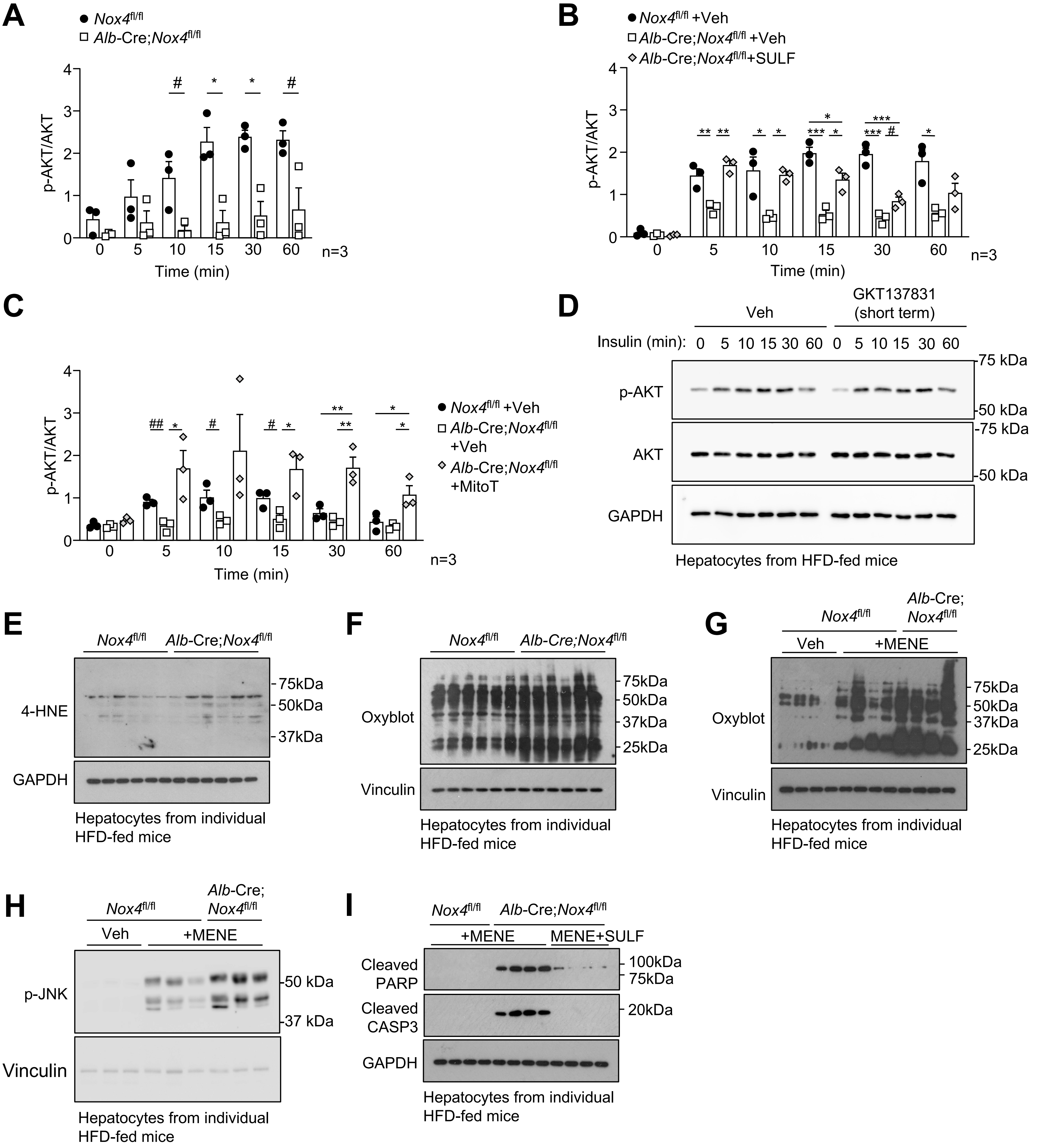
A
Nox4fl/fl
Alb-Cre;Nox4fl/fl
p-AKT/AKT
Time (min)
n=3
B
Nox4fl/fl +Veh
Alb-Cre;Nox4fl/fl +Veh
Alb-Cre;Nox4fl/fl+SULF
p-AKT/AKT
Time (min)
n=3
C
Nox4fl/fl +Veh
Alb-Cre;Nox4fl/fl +Veh
Alb-Cre;Nox4fl/fl +MitoT
p-AKT/AKT
Time (min)
n=3
D
Veh
GKT137831 (short term)
Insulin (min): 0 5 10 15 30 60 0 5 10 15 30 60
p-AKT
AKT
GAPDH
75 kDa
50 kDa
Hepatocytes from HFD-fed mice
E
Nox4fl/fl
Alb-Cre;Nox4fl/fl
4-HNE
GAPDH
75kDa
50kDa
37kDa
Hepatocytes from individual HFD-fed mice
F
Nox4fl/fl
Alb-Cre;Nox4fl/fl
Oxyblot
Vinculin
75kDa
50kDa
37kDa
25kDa
Hepatocytes from individual HFD-fed mice
G
Nox4fl/fl
Alb-Cre; Nox4fl/fl
Veh
+MENE
Oxyblot
Vinculin
75kDa
50kDa
37kDa
25kDa
Hepatocytes from individual HFD-fed mice
H
Nox4fl/fl
Alb-Cre; Nox4fl/fl
Veh
+MENE
p-JNK
Vinculin
50 kDa
37 kDa
Hepatocytes from individual HFD-fed mice
I
Nox4fl/fl
Alb-Cre;Nox4fl/fl
+MENE
MENE+SULF
Cleaved PARP
Cleaved CASP3
GAPDH
100kDa
75kDa
20kDa
Hepatocytes from individual HFD-fed mice

Supplementary Figure 4. Oxidative stress and diminished insulin signaling in NOX4-deficient hepatocytes. 8-week-old *Nox4*$^{fl/fl}$ or *Alb*-Cre;*Nox4*$^{fl/fl}$ (C57BL/6) mice were fed a HFD for 8-10 weeks and hepatocytes isolated by a two-step collagenase perfusion method. Hepatocytes were cultured for 16 h at 5% O_2 and **a**) serum starved in HBBS for 4 h and stimulated with 1 nM insulin for the indicated times and processed for immunoblotting to monitor for AKT Ser-473 phosphorylation (p-AKT); p-AKT/AKT was quantified (relates to Fig. 5b). **b-c**) Alternatively hepatocytes were treated with vehicle (DMSO), 1 μM sulforaphane (SULF) or 20 μM mitoTEMPOL (MitoT) as indicated for 16, starved for 4 h in HBBS, stimulated with 1 nM insulin and processed for p-AKT immunoblotting (relates to Fig. 5c-d). **d**) 8-week-old C57BL/6 mice were fed a HFD for 8-10 weeks and hepatocytes isolated by a two-step collagenase perfusion method. Hepatocytes were cultured for 16 h at 5% O_2, administered GKT137831 (40 μM) in HBBS for 6 h, stimulated with 2 nM insulin for the indicated times and processed for immunoblotting to monitor for AKT Ser-473 phosphorylation (p-AKT). **e-f**) 8-week-old *Nox4*$^{fl/fl}$ or *Alb*-Cre;*Nox4*$^{fl/fl}$ (C57BL/6) mice were fed a HFD for 8-10 weeks and hepatocytes isolated by a two-step collagenase perfusion method. Hepatocytes were cultured for 16 h at 5% O_2 and processed for analysis of **e**) lipid peroxidation (4-HNE) or **f**) protein carbonylation (Oxyblot) by immunoblotting. Alternatively, hepatocytes were treated with vehicle, or 2.5 μM menadione (MENE) for 36 h and processed for immunoblotting to assess **g**) protein carbonylation, or **h**) the T183/Y185 phosphorylation and activation of the mitogen-activated protein kinase JNK (p-JNK). **i**) 8-week-old *Nox4*$^{fl/fl}$ or *Alb*-Cre;*Nox4*$^{fl/fl}$ mice were fed a HFD for 8-10 weeks and hepatocytes isolated, cultured for 16 h at 5% O_2 and then treated with vehicle or 1 μM SULF for 16 h and then 2.5 μM MENE in the presence of 1 μM SULF for 24 h and processed for quantified results are shown (mean ± SEM) for the indicated number experiments.

Significance in (a) determined using Student's t-test and in (b, c) using a one-way ANOVA; # in (a, c) indicates significance using Student's t-tests.

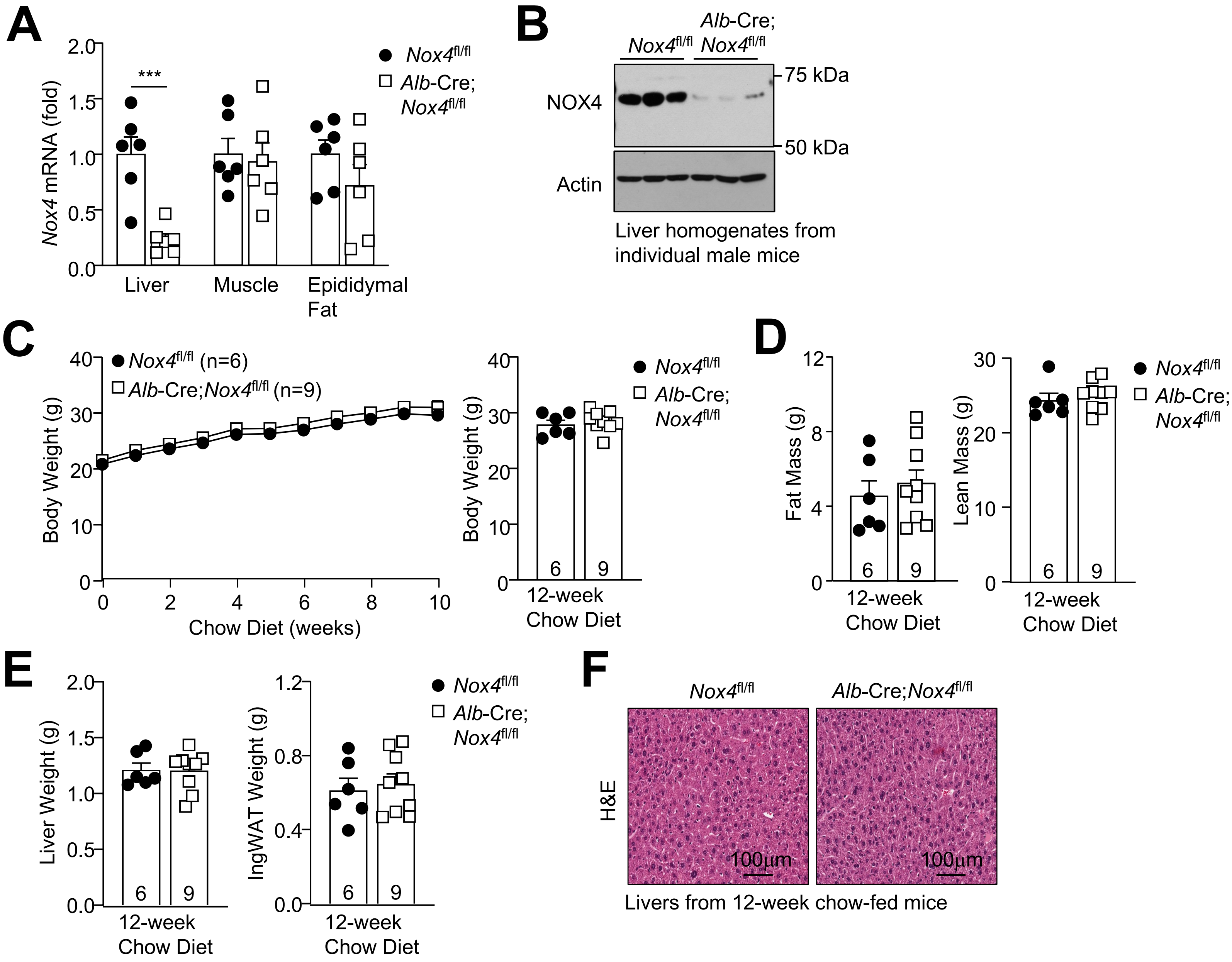
A
Nox4 mRNA (fold)
***
Liver
Muscle
Epididymal Fat
Nox4fl/fl
Alb-Cre; Nox4fl/fl
B
Nox4fl/fl
Alb-Cre; Nox4fl/fl
NOX4
Actin
75 kDa
50 kDa
Liver homogenates from individual male mice
C
Nox4fl/fl (n=6)
Alb-Cre;Nox4fl/fl (n=9)
Body Weight (g)
Chow Diet (weeks)
12-week Chow Diet
D
Fat Mass (g)
Lean Mass (g)
12-week Chow Diet
E
Liver Weight (g)
IngWAT Weight (g)
12-week Chow Diet
F
H&E
100µm
Livers from 12-week chow-fed mice

Supplementary Figure 5. Unaltered body weight and adiposity in chow fed male Alb-Cre;Nox4$^{fl/fl}$ mice. **a-f)** 8-week-old *Nox4*$^{fl/fl}$ and *Alb*-Cre;*Nox4*$^{fl/fl}$ male mice were fed a standard chow diet for 10 weeks. **a**) Tissues were processed for qPCR or **b**) livers were processed for immunoblotting to assess NOX4 expression. **c**) Body weights, **d**) body composition (DEXA) and **e**) liver and inguinal (Ing) white adipose tissue (WAT) weights were determined. **f**) Livers were formalin-fixed and paraffin-embedded and processed for histology (H&E: hematoxylin and eosin). Representative and quantified results are shown (means ± SEM) for the indicated number of mice. Significance in (a) determined using a Student's t-test.

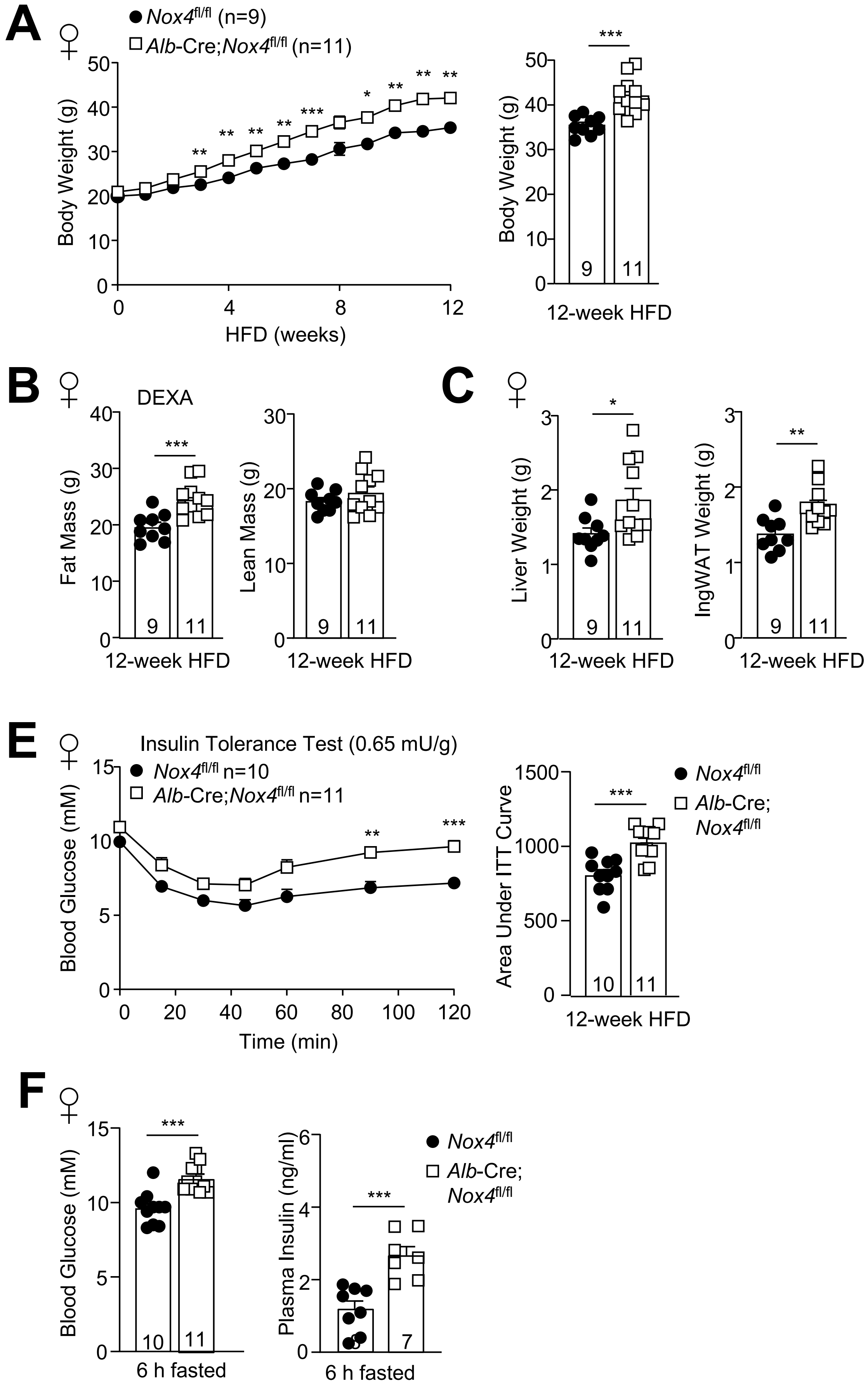
A
♀
Nox4fl/fl (n=9)
Alb-Cre;Nox4fl/fl (n=11)
Body Weight (g)
HFD (weeks)
12-week HFD
B
DEXA
Fat Mass (g)
Lean Mass (g)
12-week HFD
C
Liver Weight (g)
IngWAT Weight (g)
12-week HFD
D
H&E
Nox4fl/fl
Alb-Cre;Nox4fl/fl
200μm
Livers from 12 week HFD-fed mice
E
Insulin Tolerance Test (0.65 mU/g)
Nox4fl/fl n=10
Alb-Cre;Nox4fl/fl n=11
Blood Glucose (mM)
Time (min)
Area Under ITT Curve
12-week HFD
F
Blood Glucose (mM)
Plasma Insulin (ng/ml)
6 h fasted
Nox4fl/fl
Alb-Cre; Nox4fl/fl

Supplementary Figure 6. Hepatic Nox4 deletion promotes obesity, steatosis and insulin resistance in female mice. **a-f)** 8-week-old *Nox4*$^{fl/fl}$ and *Alb*-Cre;*Nox4*$^{fl/fl}$ female mice were fed a HFD for 12 weeks. **a**) Body weights. **b**) Body composition (DEXA). **c**) Liver and IngWAT weights. **d**) Livers were formalin-fixed and paraffin-embedded and processed for histology (H&E). **e**) Mice were subjected to insulin tolerance tests [ITTs; areas under ITT curves were determined and arbitrary units (AU) shown], or **f**) fasted for 6 h and blood glucose and plasma insulin levels determined. Representative and quantified results are shown (means ± SEM) for the indicated number of mice. Significance determined using (a-c, e-f) Student's t-tests, or (ITTs in a, e) a two-way ANOVA.

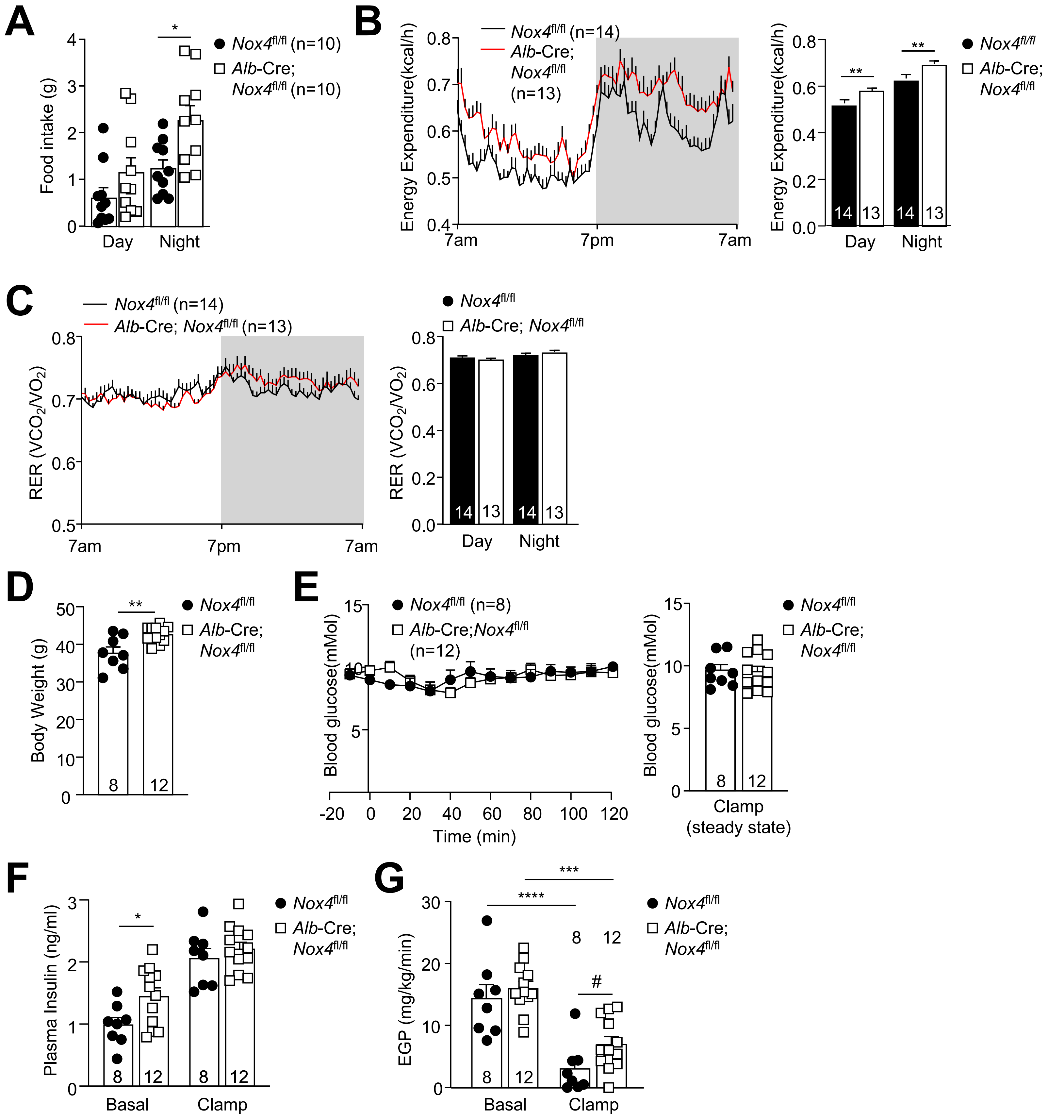

A
Food intake (g)
Nox4fl/fl (n=10)
Alb-Cre; Nox4fl/fl (n=10)
Day
Night
B
Energy Expenditure(kcal/h)
Nox4fl/fl (n=14)
Alb-Cre; Nox4fl/fl (n=13)
7am
7pm
7am
Nox4fl/fl
Alb-Cre; Nox4fl/fl
C
RER (VCO2/VO2)
Nox4fl/fl (n=14)
Alb-Cre; Nox4fl/fl (n=13)
D
Body Weight (g)
E
Blood glucose(mMol)
Nox4fl/fl (n=8)
Alb-Cre;Nox4fl/fl (n=12)
Time (min)
Clamp (steady state)
F
Plasma Insulin (ng/ml)
Basal
Clamp
G
EGP (mg/kg/min)

Supplementary Figure 7. Hepatic Nox4 deletion increases food intake, energy expenditure and EGP in male mice. **a-g)** 8-week-old *Nox4*$^{fl/fl}$ and *Alb*-Cre;*Nox4*$^{fl/fl}$ male mice were fed a HFD for 12 weeks and **a**) diurnal food intake (Promethion), **b**) energy expenditure and **c**) respiratory exchange ratios (RERs) were determined by indirect calorimetry (Promethion). **d-g**) Alternatively, after a 6 h fast, conscious and unrestrained mice were subjected to hyperinsulinaemic-euglycaemic clamps. **d**) Body weights, **e**) blood glucose, **f**) plasma insulin levels and **g**) endogenous glucose production (EGP) during basal and insulin-stimulated (clamp) conditions were determined. Representative and quantified results are shown (means ± SEM) for the indicated number of mice. Significance determined using (a, b, f, g) two-way ANOVA, or (d) Student's t-test. # in (g) corresponds to *Alb*-Cre;*Nox4*$^{fl/fl}$ versus *Nox4*$^{fl/fl}$ using a Student's t-test.

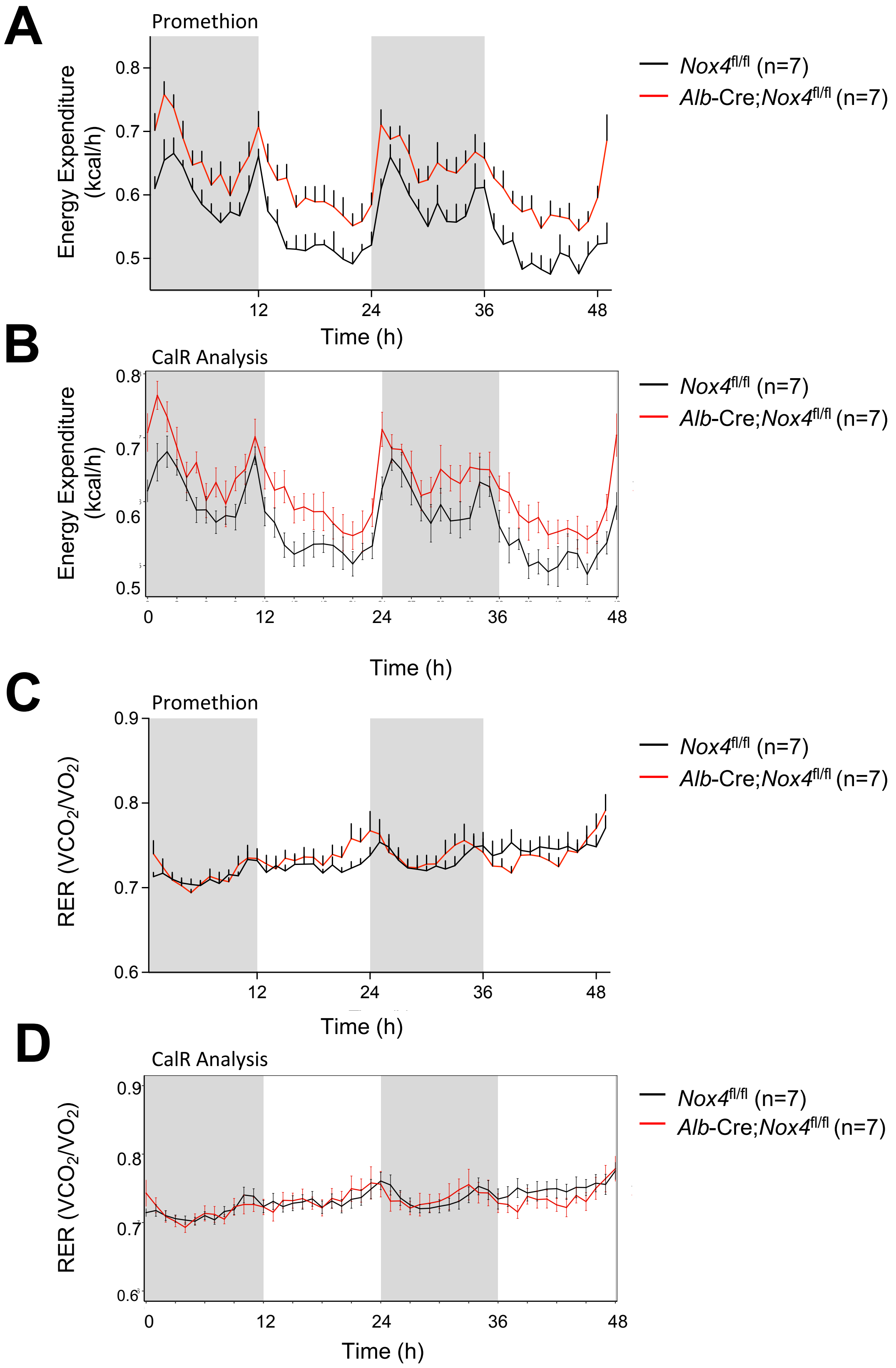
A
Promethion
Energy Expenditure
(kcal/h)
0.8
0.7
0.6
0.5
12
24
36
48
Time (h)
Nox4fl/fl (n=7)
Alb-Cre;Nox4fl/fl (n=7)
B
CalR Analysis
Energy Expenditure
(kcal/h)
0.8
0.7
0.6
0.5
0
12
24
36
48
Time (h)
Nox4fl/fl (n=7)
Alb-Cre;Nox4fl/fl (n=7)
C
Promethion
RER (VCO2/VO2)
0.9
0.8
0.7
0.6
12
24
36
48
Time (h)
Nox4fl/fl (n=7)
Alb-Cre;Nox4fl/fl (n=7)
D
CalR Analysis
RER (VCO2/VO2)
0.9
0.8
0.7
0.6
0
12
24
36
48
Time (h)
Nox4fl/fl (n=7)
Alb-Cre;Nox4fl/fl (n=7)

Supplementary Figure 8. Hepatic Nox4 deletion increases energy expenditure but does not alter RER in male mice. 8-week-old *Nox4*$^{fl/fl}$ and *Alb*-Cre;*Nox4*$^{fl/fl}$ male mice were fed a HFD for 12 weeks and **a, b**) energy expenditure and **c, d**) respiratory exchange ratios (RERs) were determined by indirect calorimetry (Promethion) and analysed **a**, **c**) without normalising for body weight, or **b**, **d**) using CalR software.

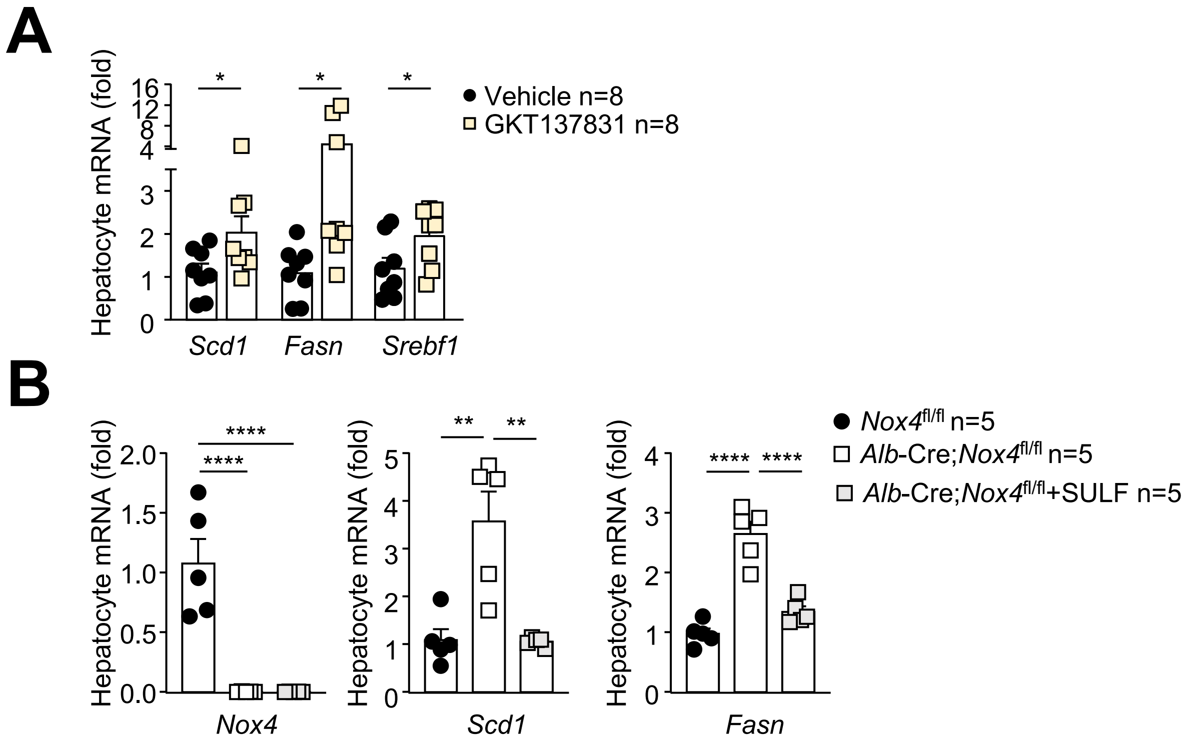

A
Hepatocyte mRNA (fold)
Vehicle n=8
GKT137831 n=8
Scd1
Fasn
Srebf1
B
Nox4fl/fl n=5
Alb-Cre;Nox4fl/fl n=5
Alb-Cre;Nox4fl/fl+SULF n=5
Nox4
Scd1
Fasn

Supplementary Figure 9. Hepatocyte NOX4 deletion or inhibition increases lipogenic gene expression. **a)** 8-week-old C57BL/6 mice were fed a HFD for 8-10 weeks and hepatocytes isolated by a two-step collagenase perfusion method. Hepatocytes were cultured for 16 h at 5% O_2 and were then administered GKT137831 (40 µM) twice per day for 48 h. Hepatocytes were then processed for qPCR to assess lipogenic gene expression. **b**) 8-week-old *Nox4*$^{fl/fl}$ or *Alb*-Cre;*Nox4*$^{fl/fl}$ (C57BL/6) mice were fed a HFD for 8-10 weeks and hepatocytes isolated by a two-step collagenase perfusion method. Hepatocytes were cultured for 16 h at 5% O_2, and then treated with vehicle (DMSO) or 1 µM sulforaphane (SULF) for 48 h to assess *Nox4* and lipogenic (*Scd1*, *Fasn*) gene expression. Representative and quantified results are shown (means ± SEM) for the indicated number of mice. Significance determined using (a) Student's t-test, or (b) a one-way ANOVA.

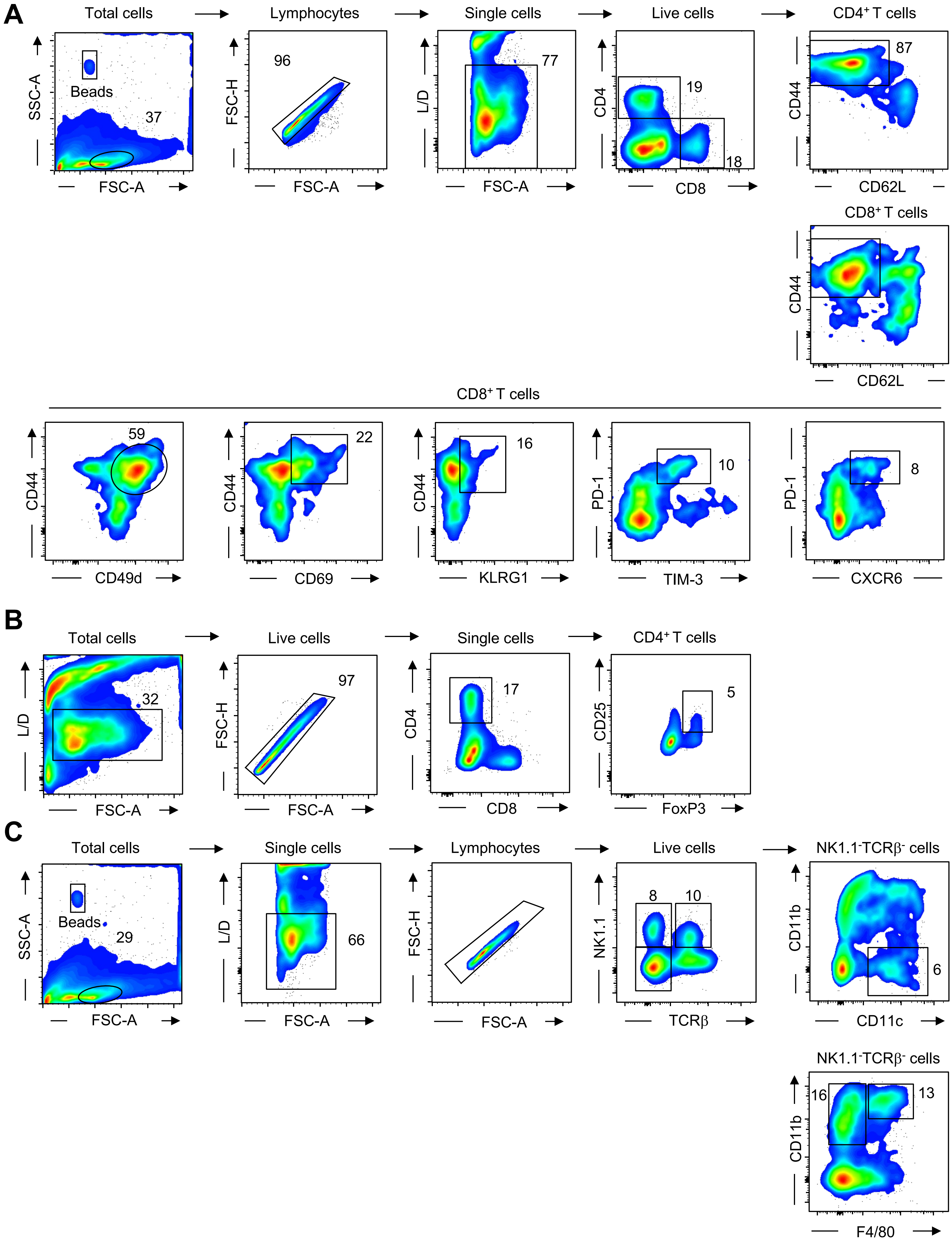
A
Total cells
Lymphocytes
Single cells
Live cells
CD4+ T cells
SSC-A
FSC-A
Beads
37
FSC-H
96
L/D
77
CD4
CD8
19
18
CD44
CD62L
87
CD8+ T cells
CD8+ T cells
CD49d
59
CD69
22
KLRG1
16
PD-1
TIM-3
10
CXCR6
8
B
Total cells
Live cells
Single cells
CD4+ T cells
L/D
FSC-A
32
FSC-H
97
CD4
CD8
17
CD25
FoxP3
5
C
Total cells
Single cells
Lymphocytes
Live cells
NK1.1-TCRβ- cells
SSC-A
FSC-A
Beads
29
L/D
66
FSC-H
NK1.1
TCRβ
8
10
CD11b
CD11c
6
NK1.1-TCRβ- cells
F4/80
16
13

Supplementary Figure 10. Gating strategy for the analysis of hepatic lymphocytes. **a**) Lymphocytes were stained with fluorophore-conjugated antibodies for CD4, CD8, CD44, CD62L, CD49d, CD69, KLRG1, PD-1, TIM-3 and CXCR6. Live L/D Fix^{-} (LIVE/DEAD® Fixable Near IR-stain) single cells were gated for CD4^{+} and CD8^{+} T cells and the proportions of CD4^{+} or CD8^{+}CD44^{hi}CD62L^{lo} effector/memory T cells, antigen-experienced CD8^{+}CD44^{hi}CD49d^{hi} T cells, previously activated CD8^{+}CD44^{hi}CD69d^{hi} T cells, effector CD8^{+}CD44^{hi}KLRG1^{hi} T cells, exhausted CD8^{+}PD-1^{hi}TIM-3^{hi} T cells and autoreactive CD8^{+}PD-1^{hi}CXCR6^{hi} T cells were determined by flow cytometry. **b**) Lymphocytes were stained with fluorophore-conjugated antibodies for CD4, CD8, CD25, CD45 and intracellular FoxP3. Live L/D Fix^{-} single cells were gated for CD4^{+} T cells and the proportions of CD4^{+}CD25^{hi}FoxP3^{+} T_{regs} were determined by flow cytometry. **c**) Lymphocytes were stained with fluorophore-conjugated antibodies for NK1.1, TCRβ, CD11b and F4/80. Live L/D Fix^{-} single cells were gated for NK1.1^{+}TCRβ^{-}, NK1.1^{+}TCRβ^{+} and NK1.1^{-}TCRβ^{-} and the proportions of NK1.1^{+}TCRβ^{-} natural killer (NK) cells, NK1.1^{+}TCRβ^{+} NK T cells, CD11b^{-} CD11c^{+} lymphoid dendritic cells (DCs), CD11b^{hi}F4/80^{lo} hepatic macrophages and CD11b^{hi}F4/80^{hi} Kupffer cells were determined by flow cytometry. Cells were quantified with Flow-Count Fluorospheres® (Beads).

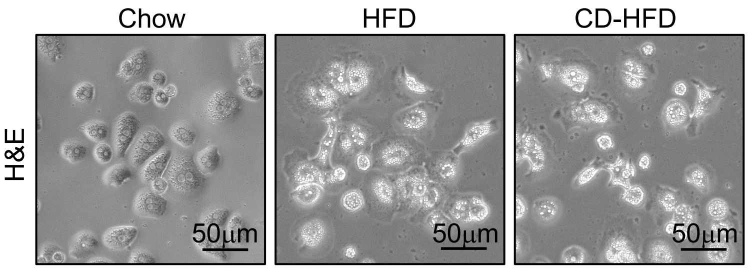

Brightfield images of hepatocytes from individual chow-, HFD- or CD-HFD-fed mice

Supplementary Figure 11. Brightfield images of hepatocytes from mice fed chow, HFD and CD-HFD. **a)** 8-week-old C57BL/6 mice were fed either a chow (4.8% fat), a HFD (23% fat) or CD-HFD (23% fat) for 12 weeks. Hepatocytes were isolated using the two-step collagenase perfusion method, cultured for 16 h at 5% O_2 and representative bright-field images taken using an EVOS M5000 cell imaging system (Thermo Fisher Scientific, Waltham, MA).

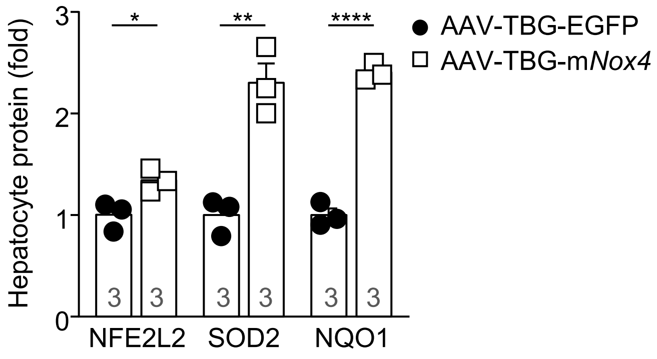
Hepatocyte protein (fold)
0
1
2
3
*
**
****
3
NFE2L2
SOD2
NQO1
AAV-TBG-EGFP
AAV-TBG-m*Nox4*

Supplementary Figure 12. Hepatocyte NOX4 overexpression increases antioxidant defense protein expression. C57BL/6 male mice were administered (intravenous) AAV-TBG-EGFP or AAV-TBG-m*Nox4* and fed a CD-HFD for up to 12 weeks and hepatocytes isolated by a two-step collagenase perfusion method. Hepatocytes were cultured for 16 h at 5% O_2 and processed for immunoblotting and quantification (relates to Fig. 15j). Quantified results are shown (means ± SEM) for the indicated number of mice. Significance determined using a Student's t-test.
