# Supplementary material for: Mitochondria- and NOX4-dependent antioxidant defense mitigates progression to nonalcoholic steatohepatitis in obesity
Source: J Clin Invest. 2024 Feb 1;134(3):e162533. doi: 10.1172/JCI162533 (PMC10849767; doi:10.1172/JCI162533)

Full unedited gel for Figure 1F

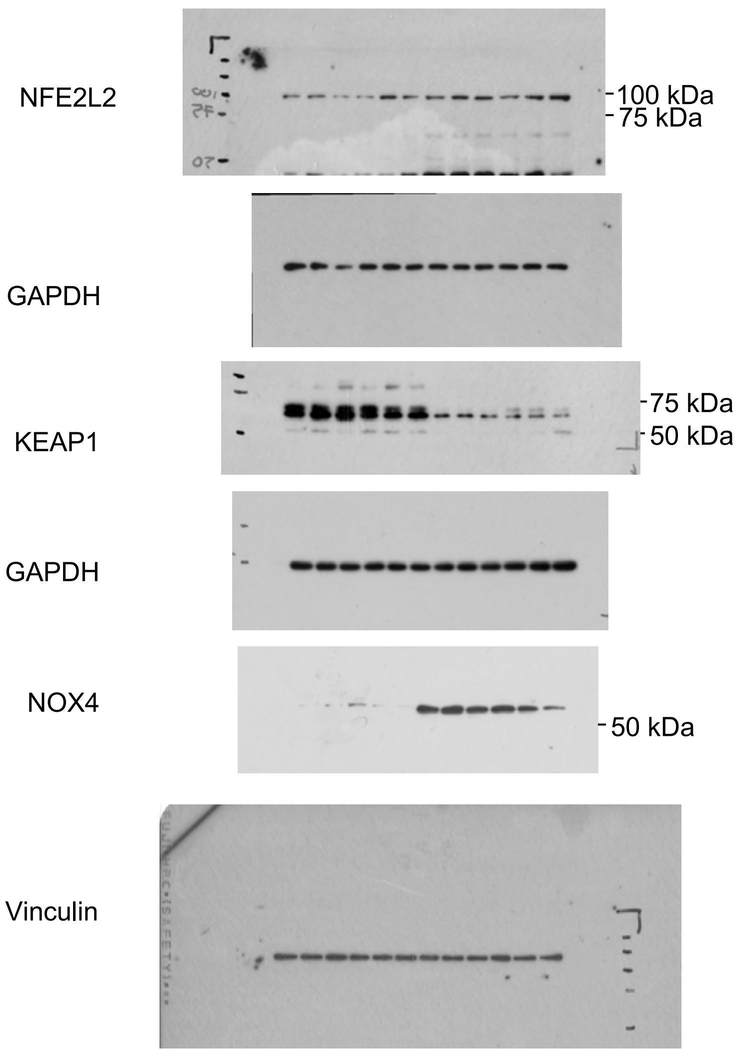

Full unedited gel for Figure 1G

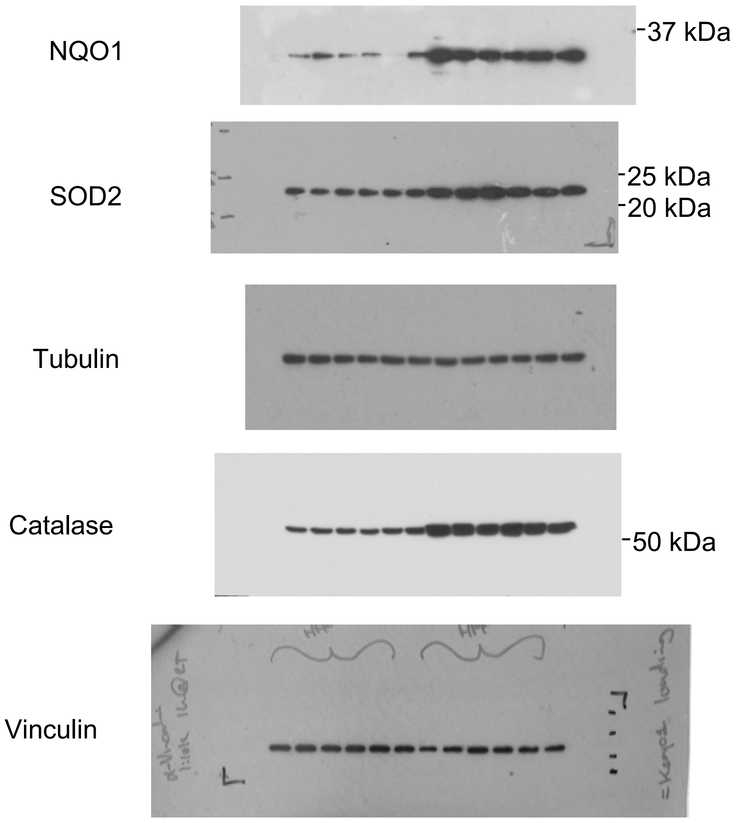

Full unedited gel for Figure 2F

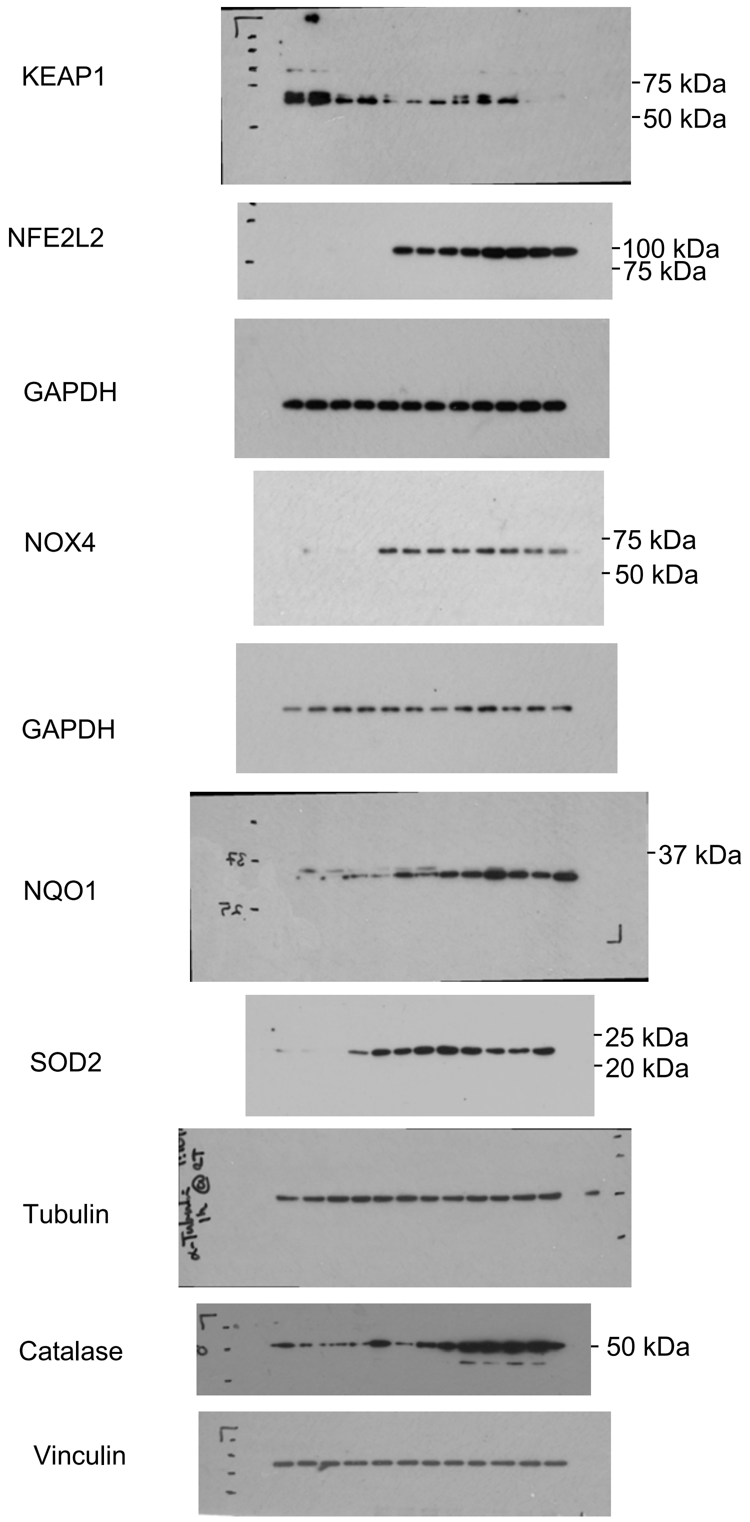

Full unedited gel for Figure 3B

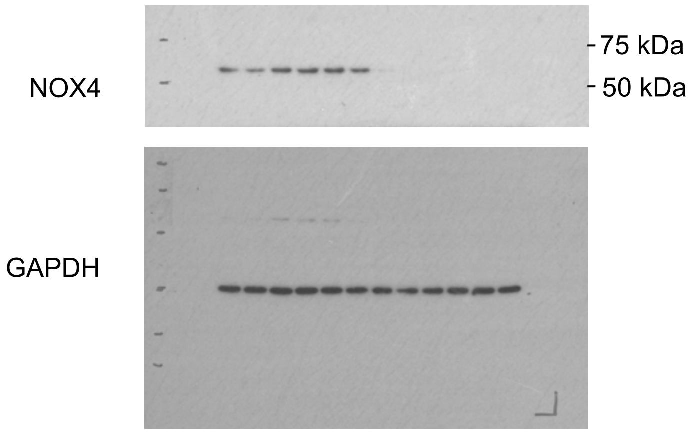

Full unedited gel for Figure 3F

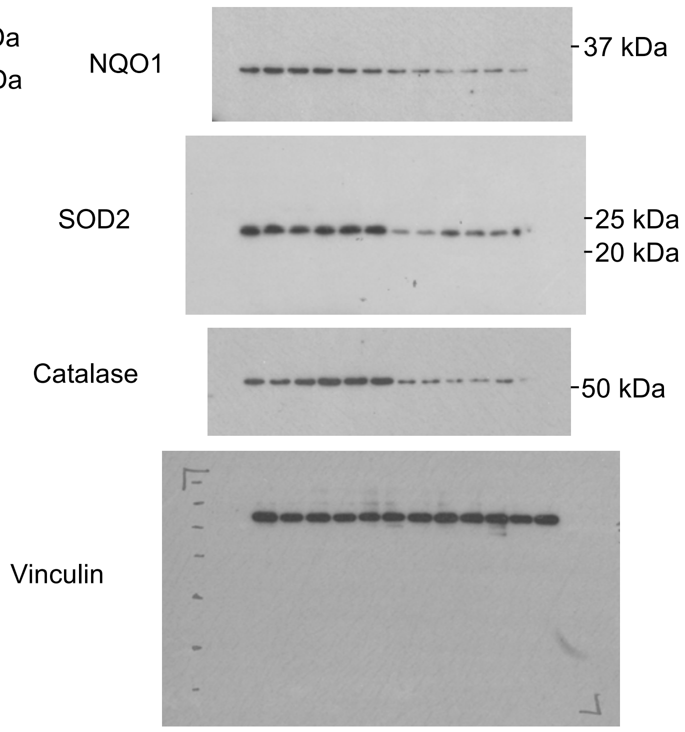

Full unedited gel for Figure 3G

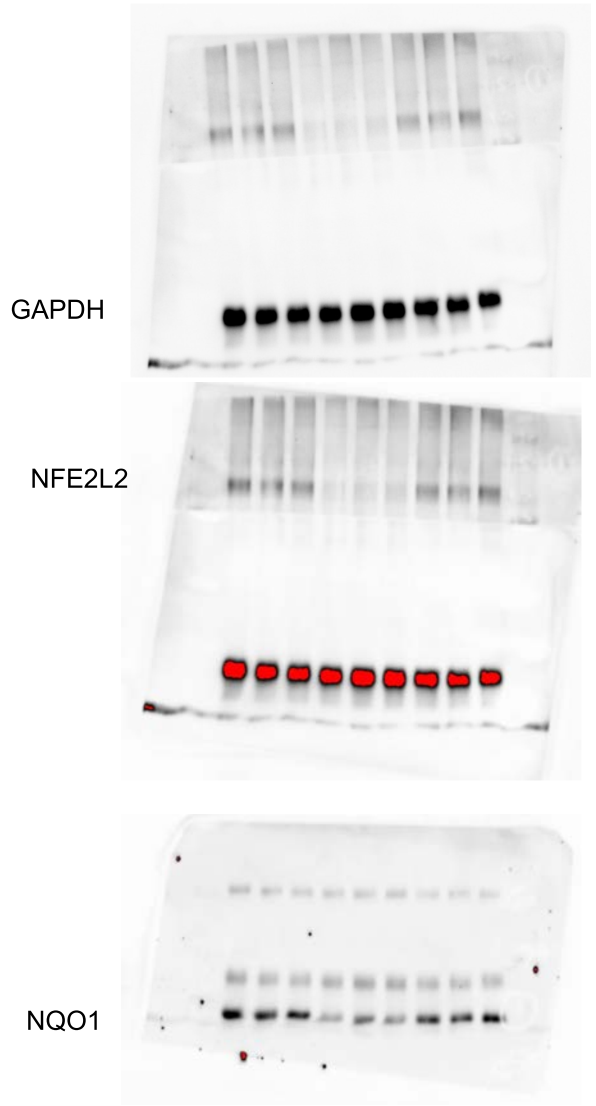

Full unedited gel for Figure 3K

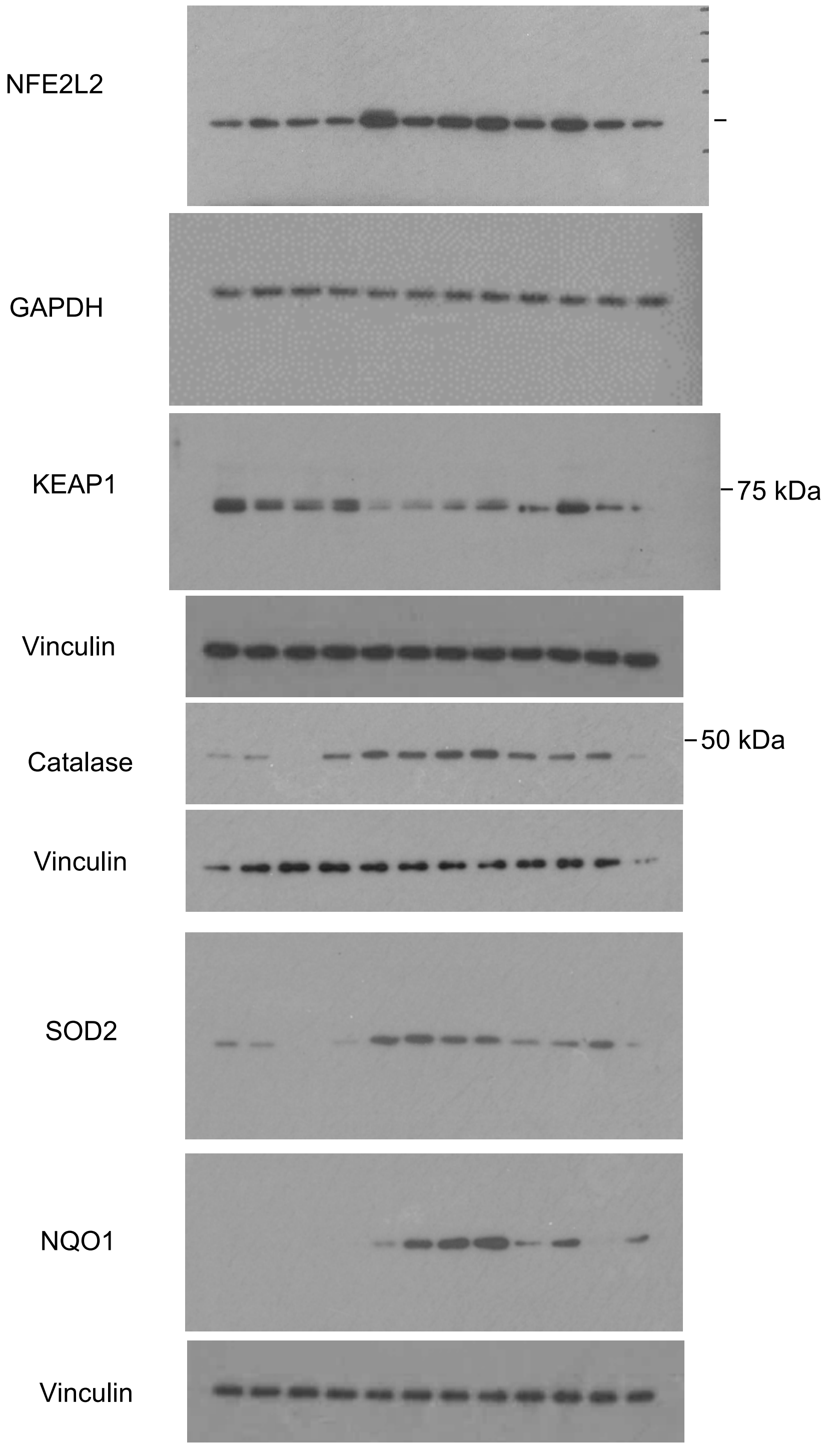

Full unedited gel for Figure 4B

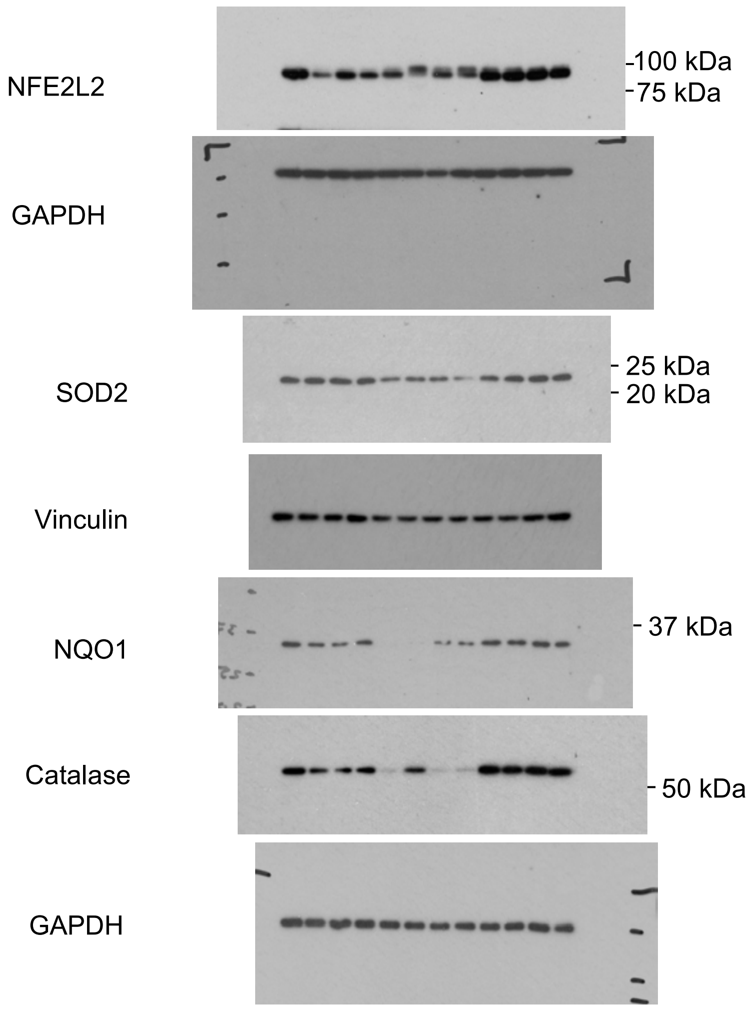

Full unedited gel for Figure 4H

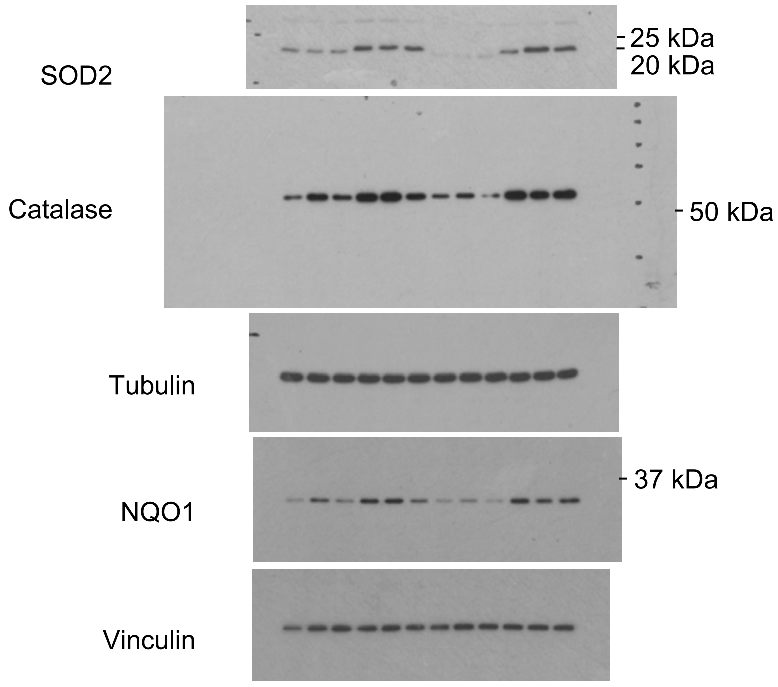

Full unedited gel for Figure 5B

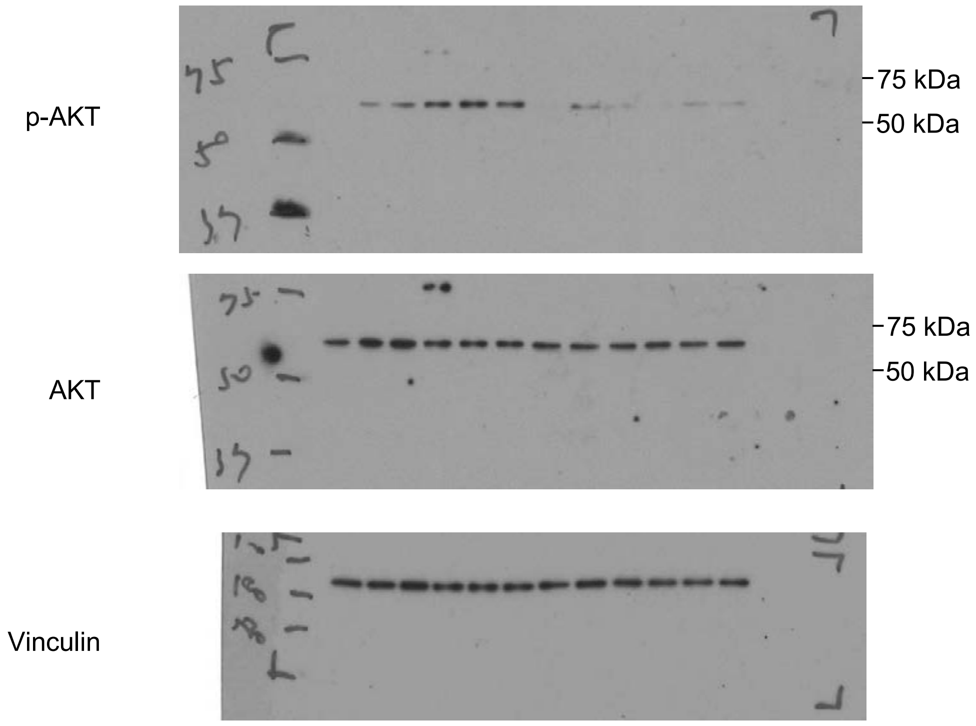

Full unedited gel for Figure 5C

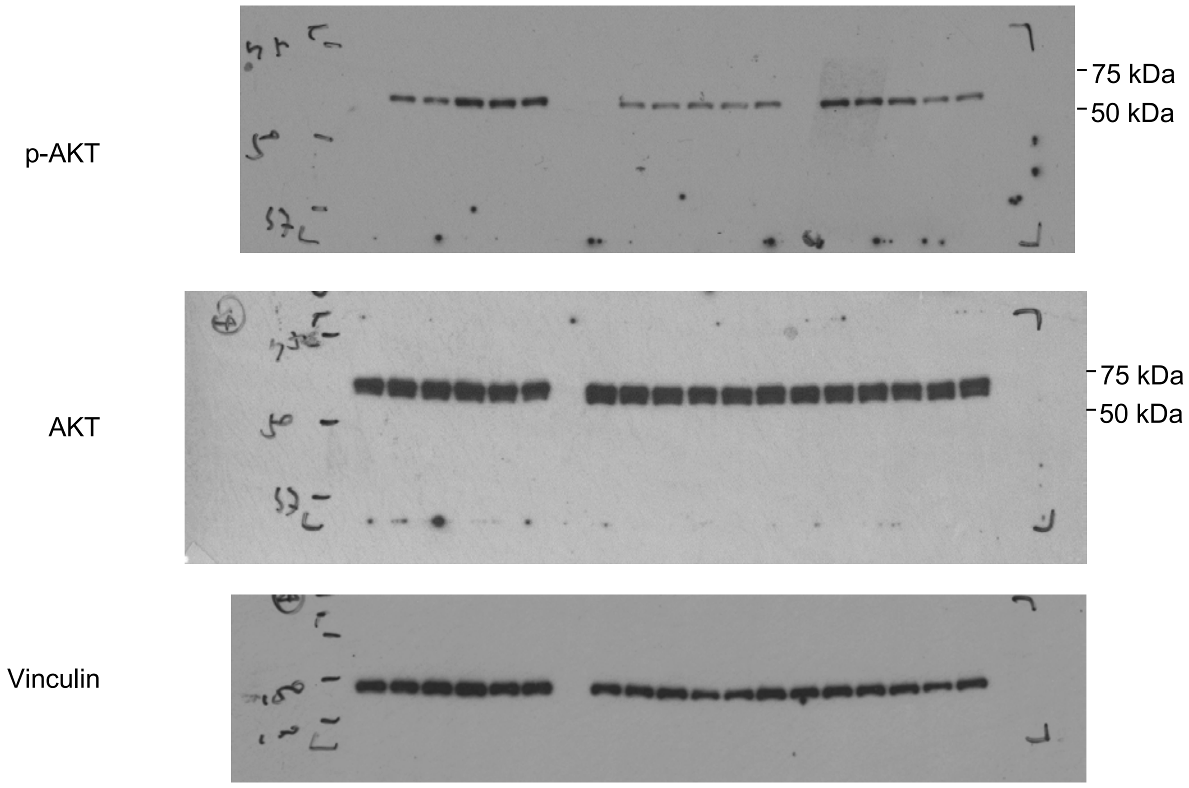

Full unedited gel for Figure 5D

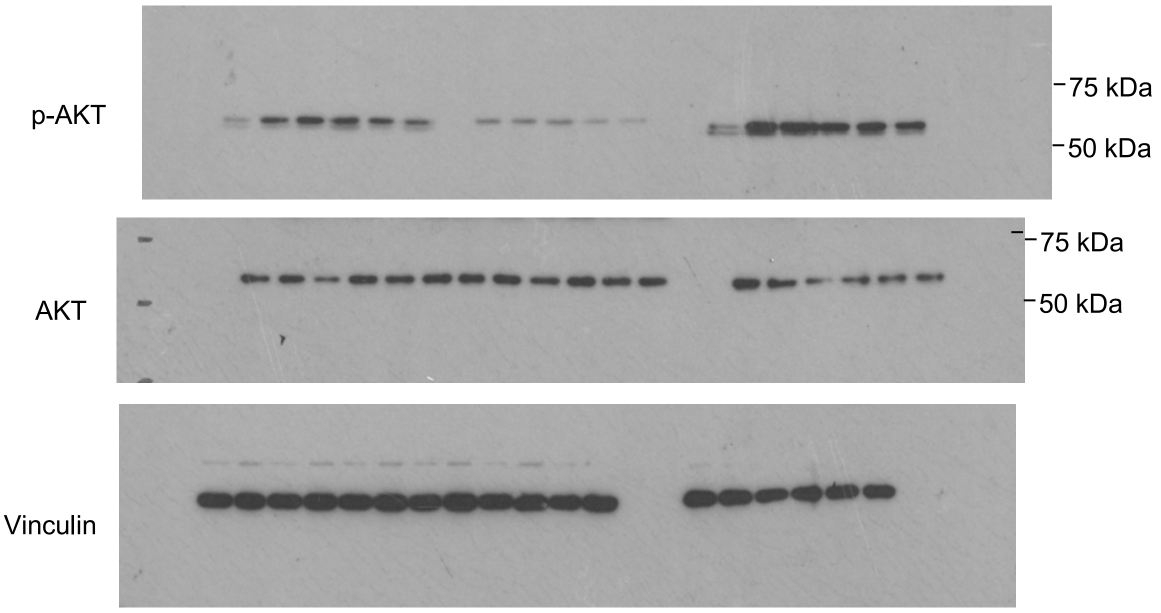

Full unedited gel for Figure 5E

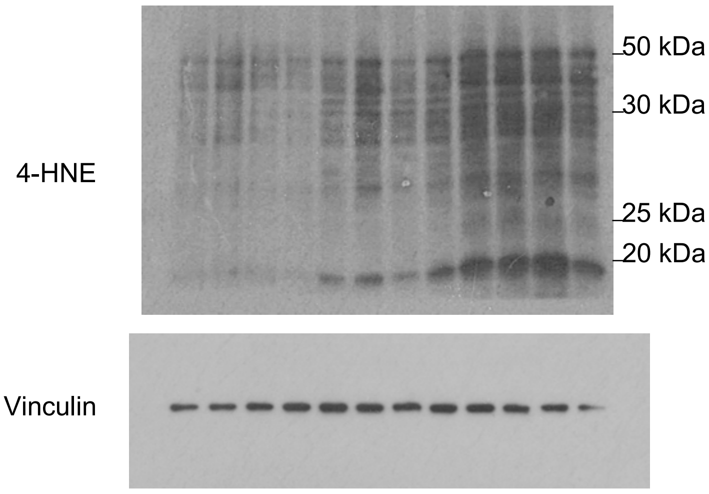

Full unedited gel for Figure 5F

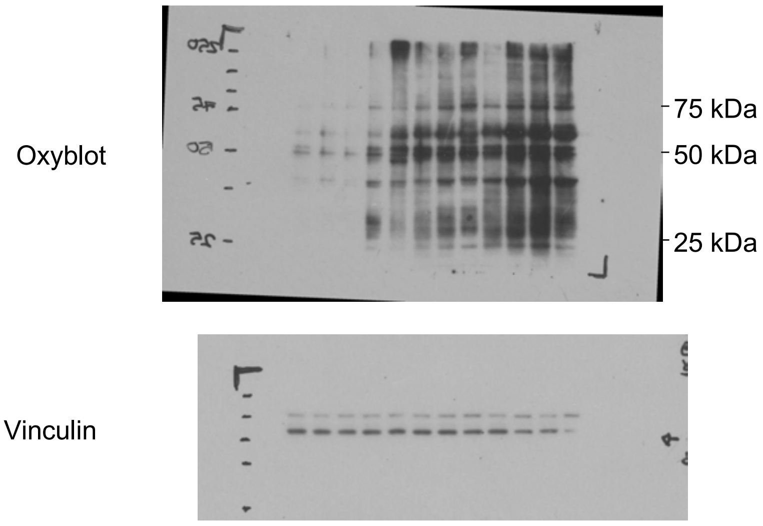

Full unedited gel for Figure 5G

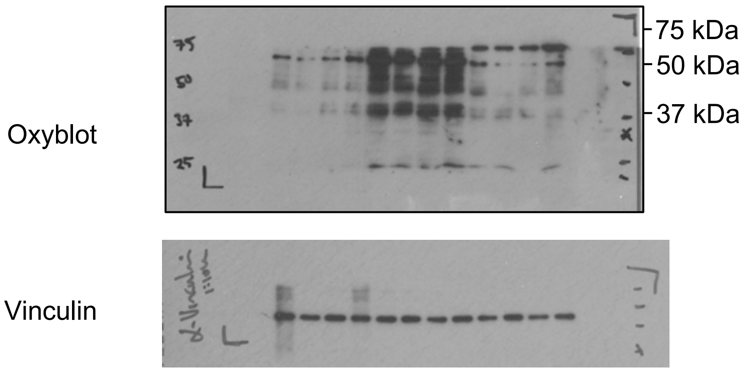

Full unedited gel for Figure 5H

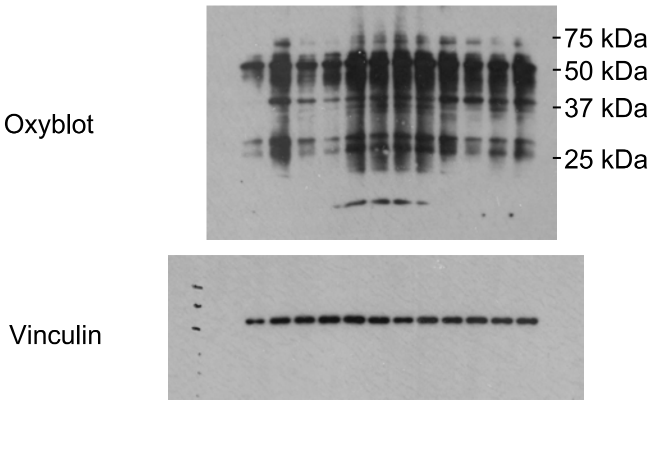

Full unedited gel for Figure 6B

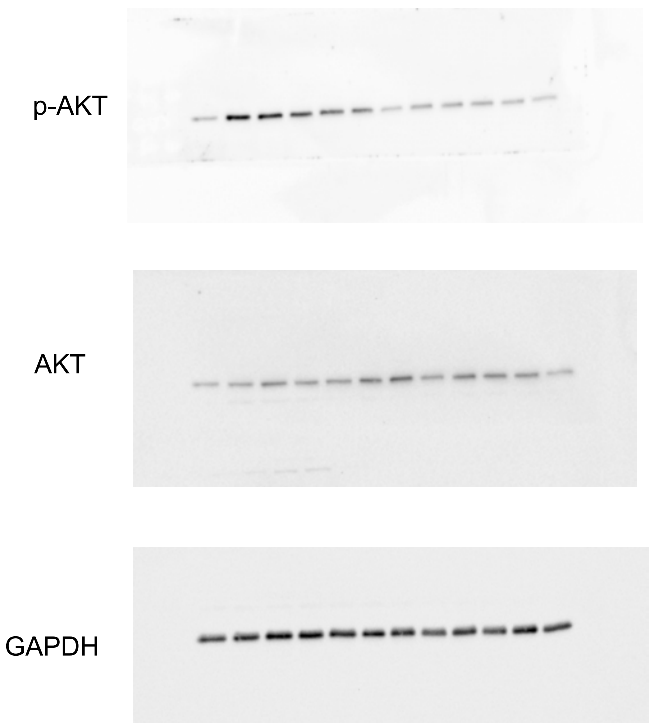

Full unedited gel for Figure 6D

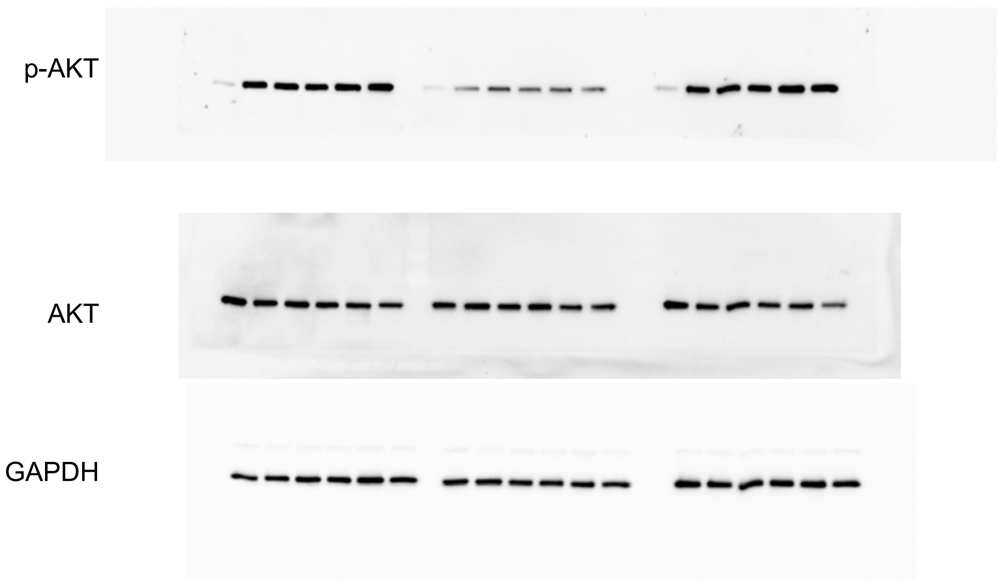

Full unedited gel for Figure 6F

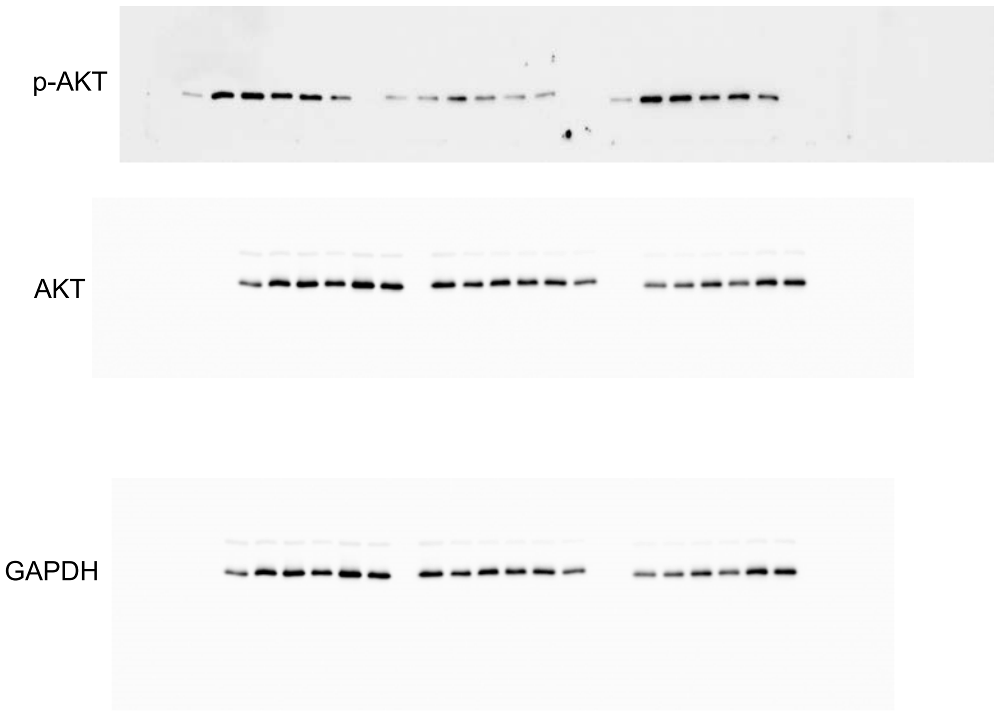

Full unedited gel for Figure 6G

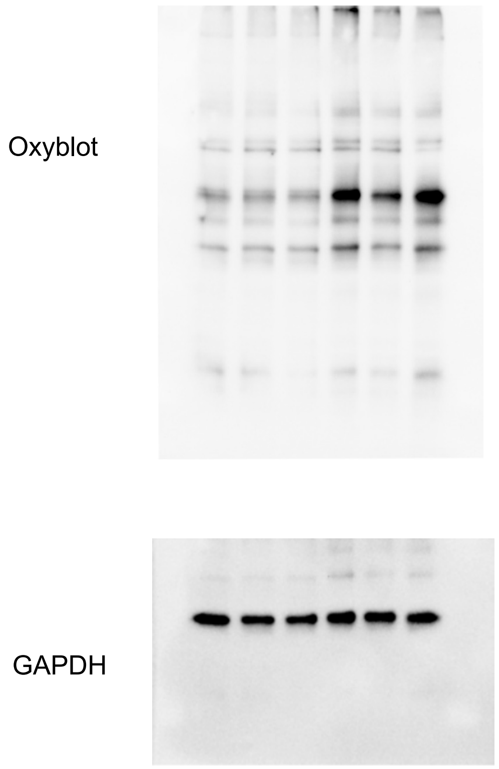

Full unedited gel for Figure 6H

Oxyblot

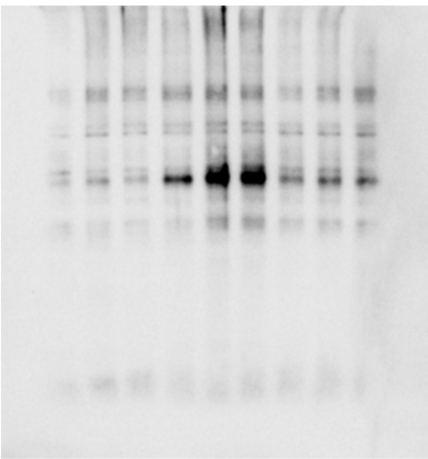

GAPDH

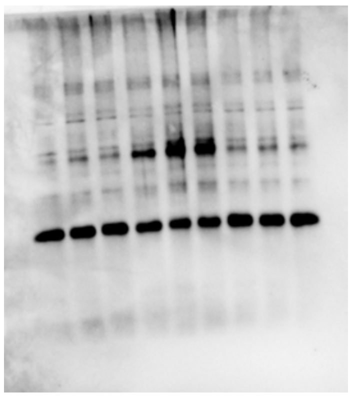

Full unedited gel for Figure 6I

Oxyblot

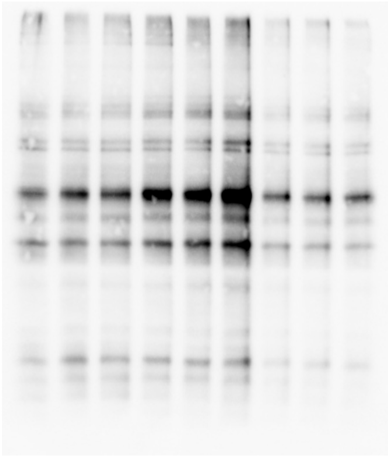

GAPDH

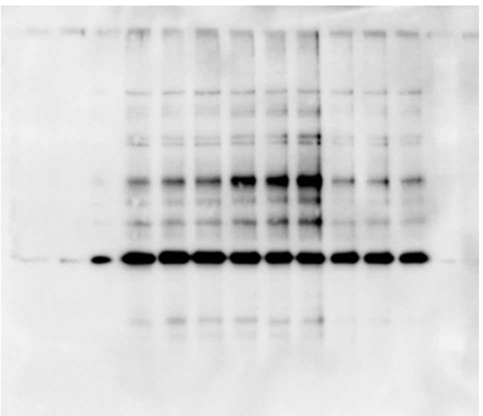

Full unedited gel for Figure 7D

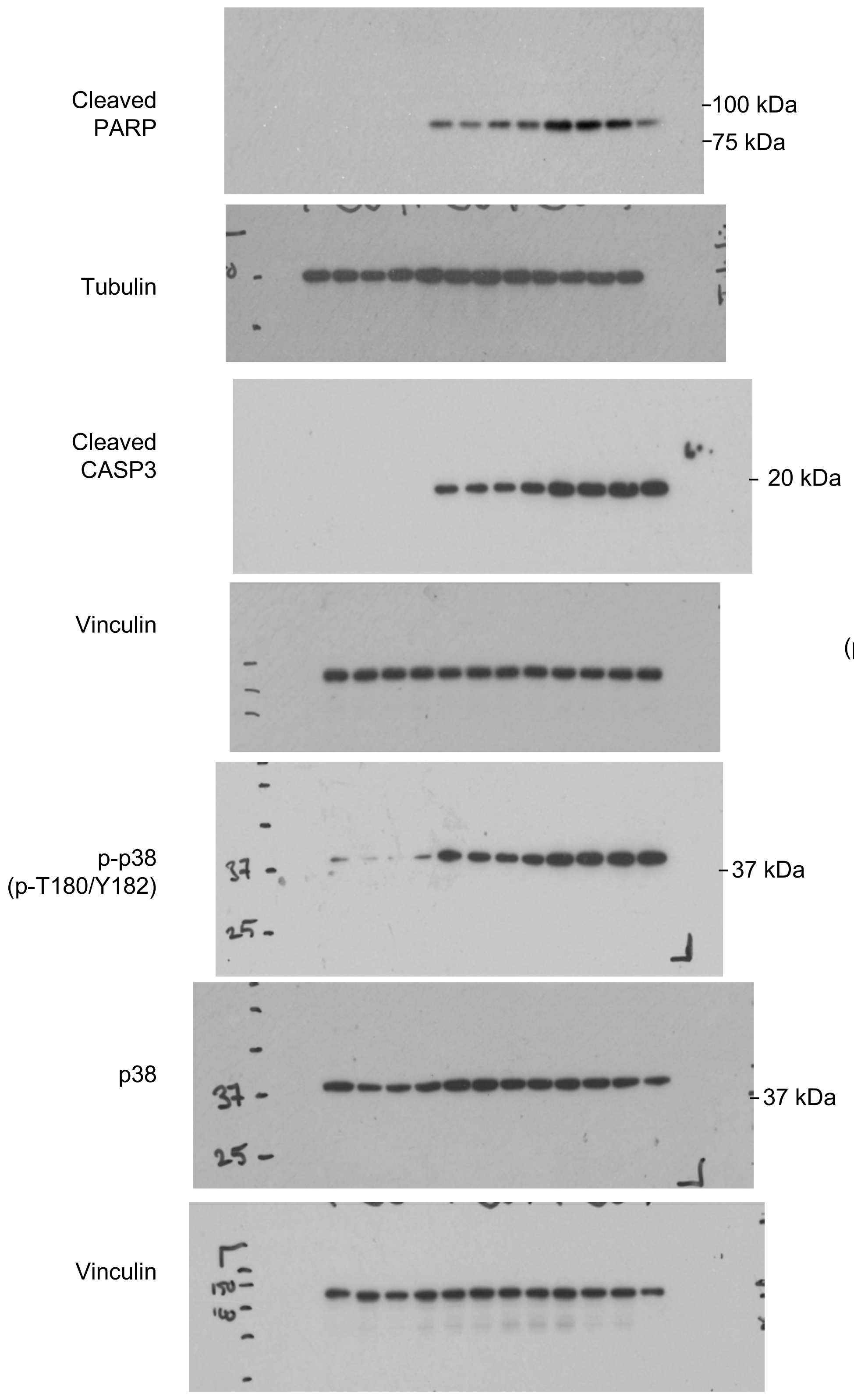

Full unedited gel for Figure 7E

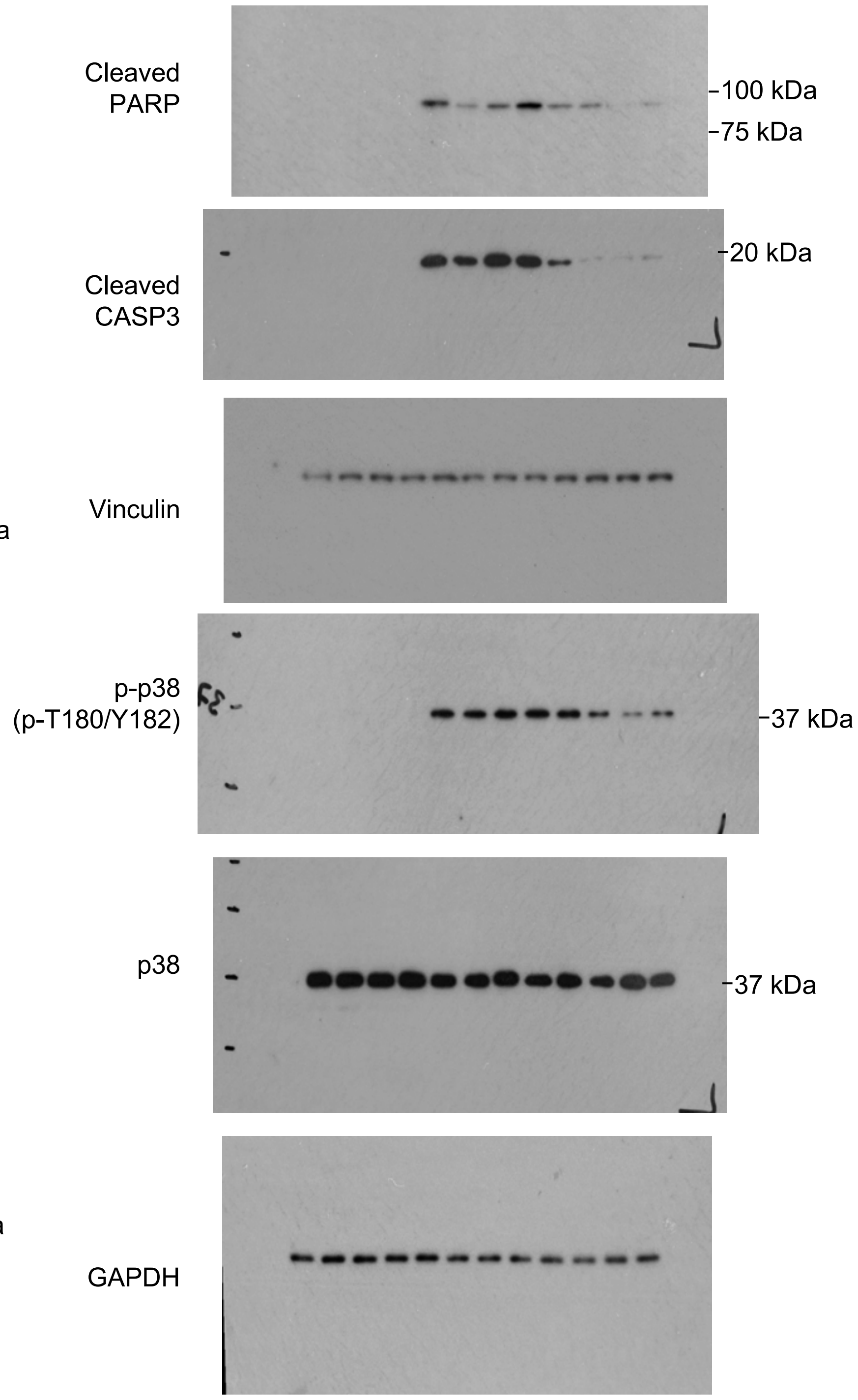

Full unedited gel for Figure 8F

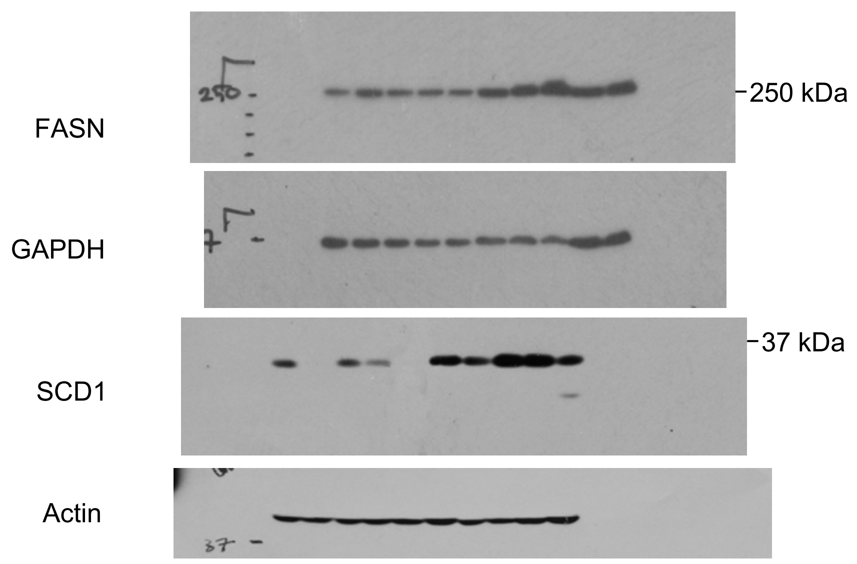

Full unedited gel for Figure 9G

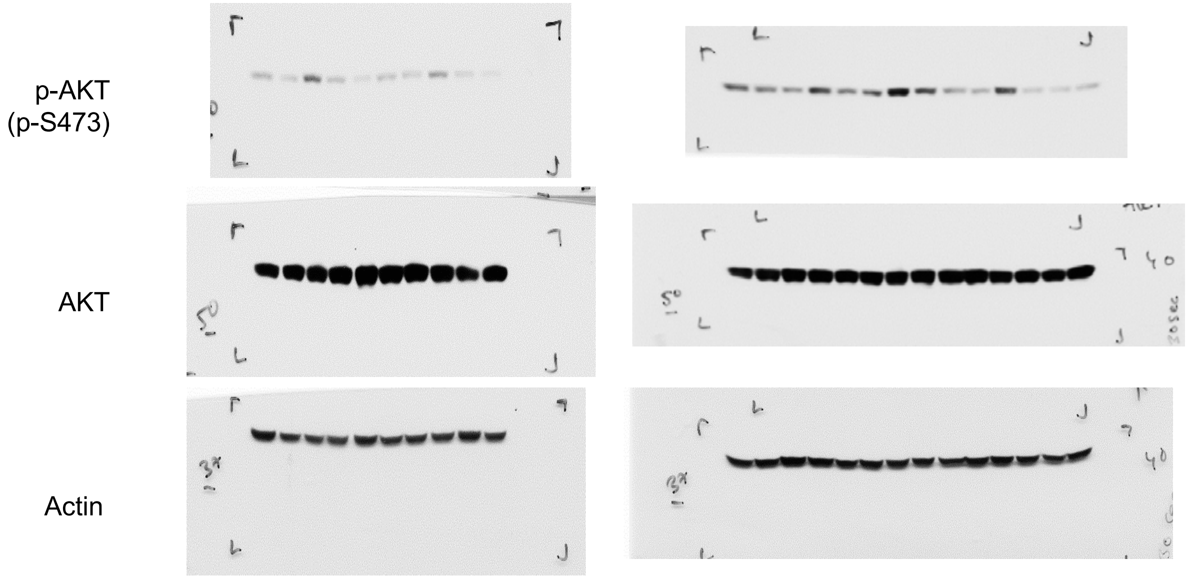

Full unedited gel for Figure 12B

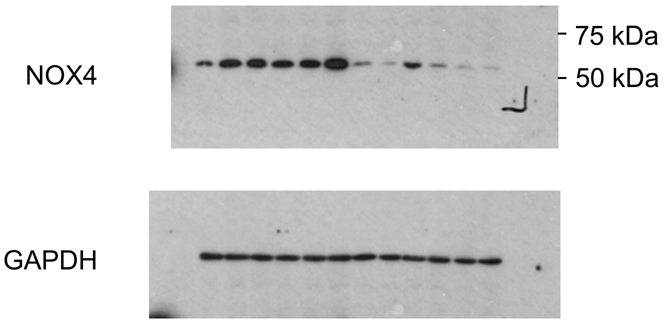

Full unedited gel for Figure 12F

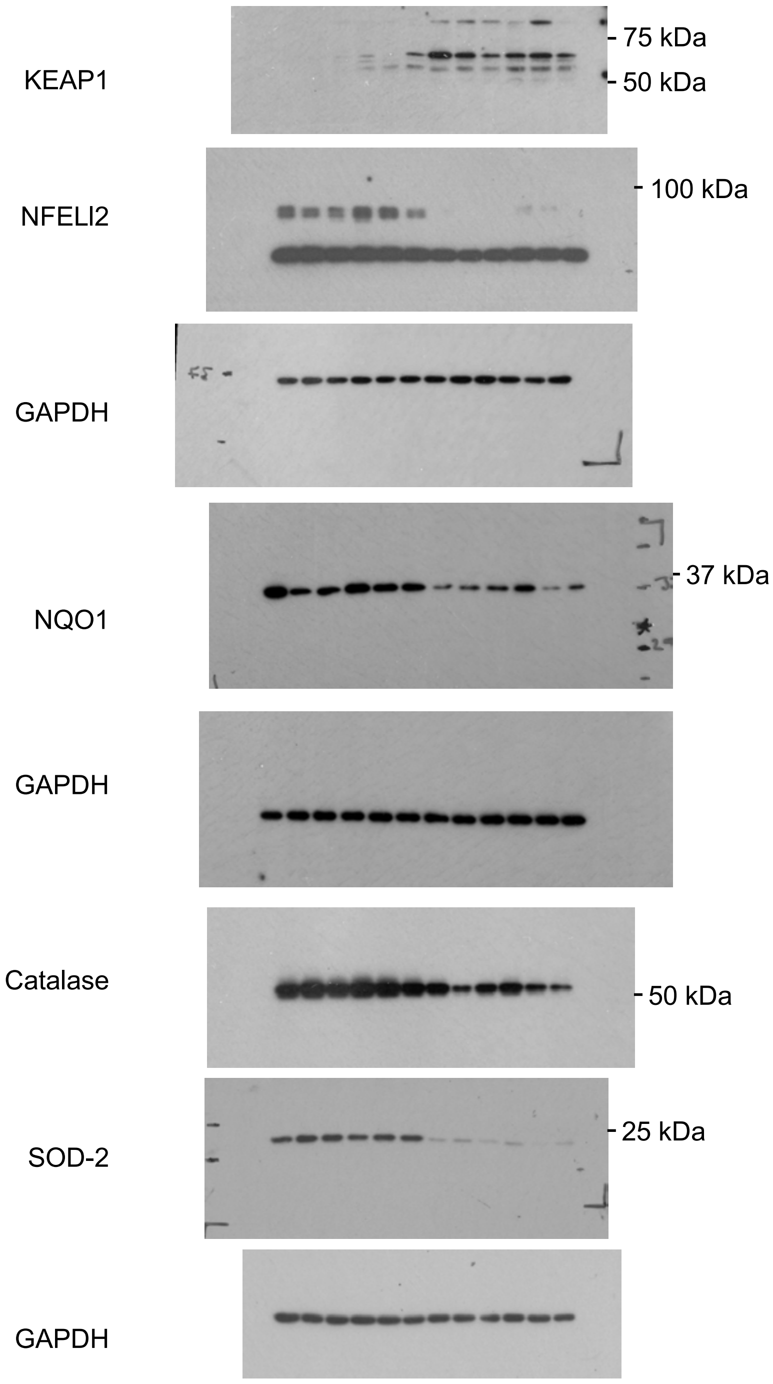

Full unedited gel for Figure 14D

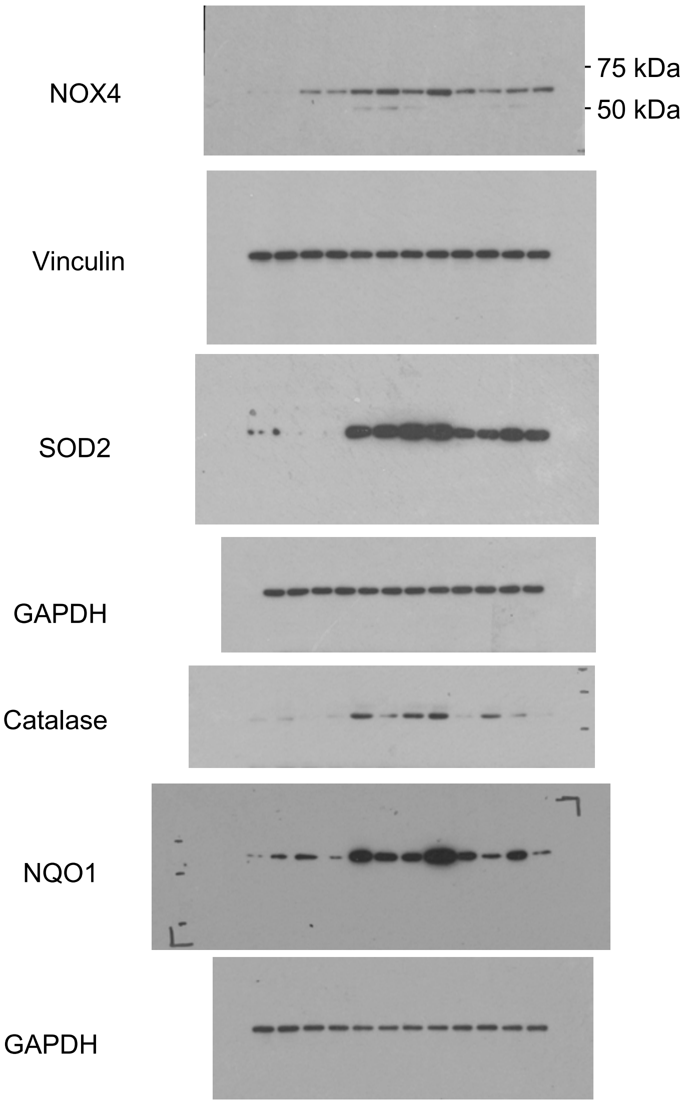

Full unedited gel for Figure 14F

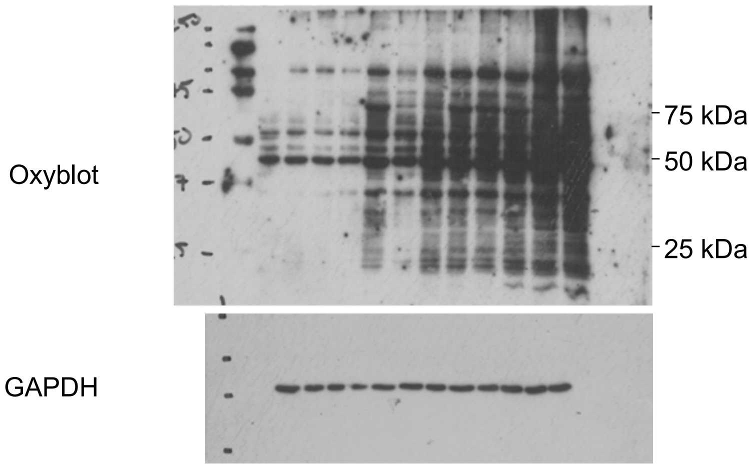

Full unedited gel for Figure 15B

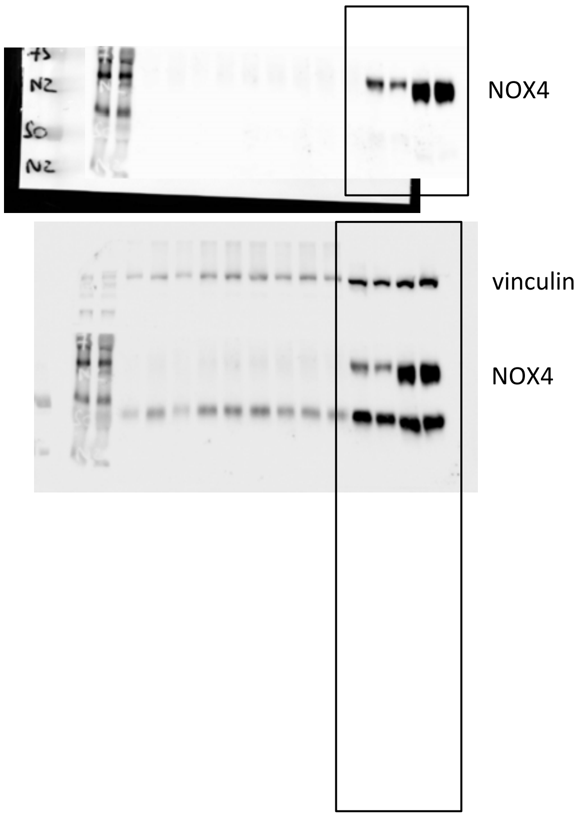

Full unedited gel for Figure 15J

NFE2L2

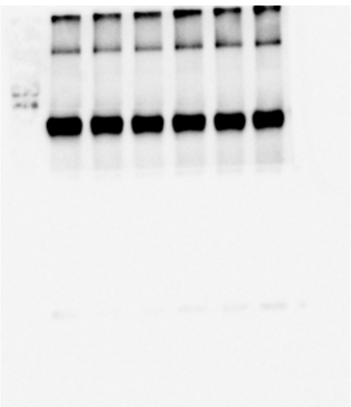

SOD2

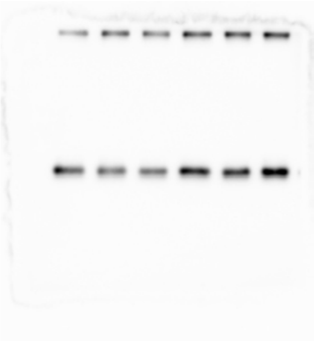

GAPDH

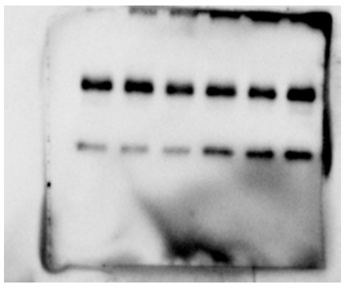

NQO1

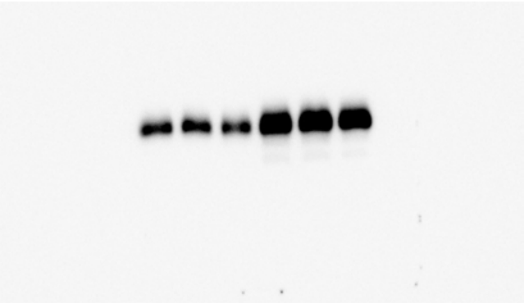

Tubulin

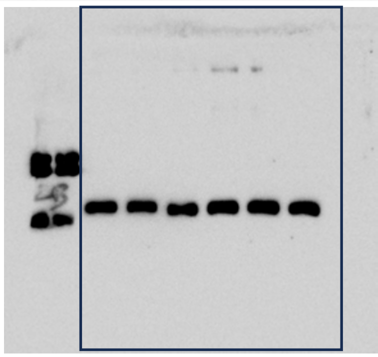

Full unedited gel for Figure 15K

Oxyblot

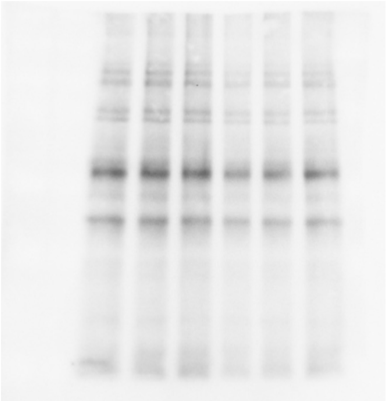

GAPDH

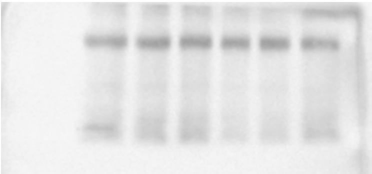

Full unedited gel for Figure S1E

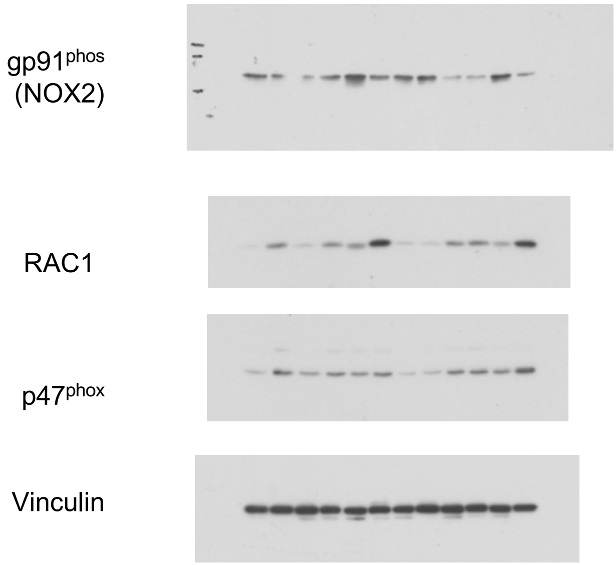

Full unedited gel for Figure S3B

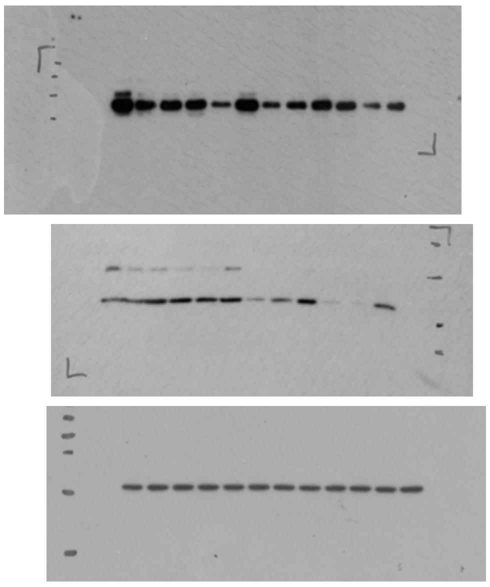

NFE2L2

NQO1

Tubulin

Full unedited gel for Figure S4D

p-AKT

AKT

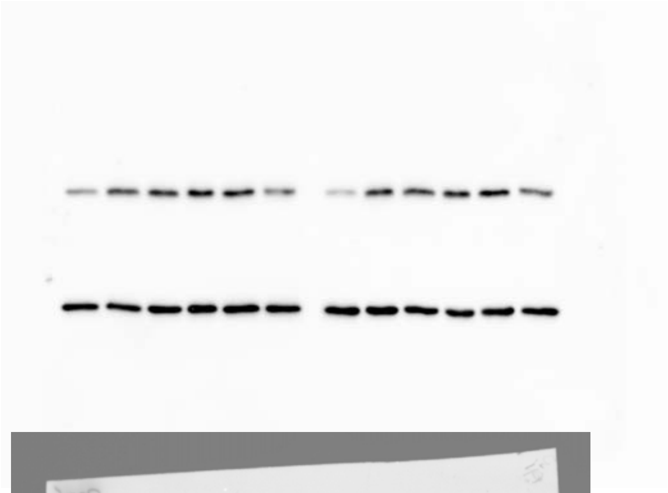

p-AKT

AKT

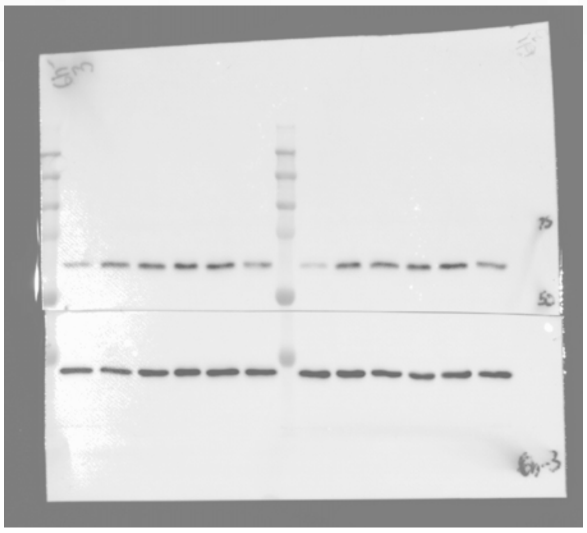

GAPDH

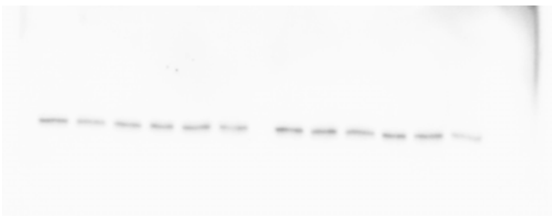

Full unedited gel for Figure S4E

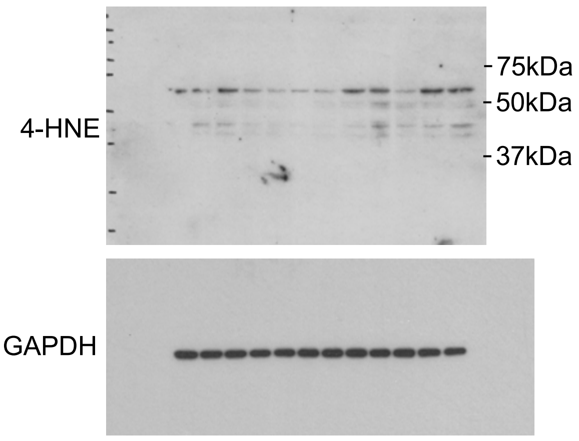

Full unedited gel for Figure S4F

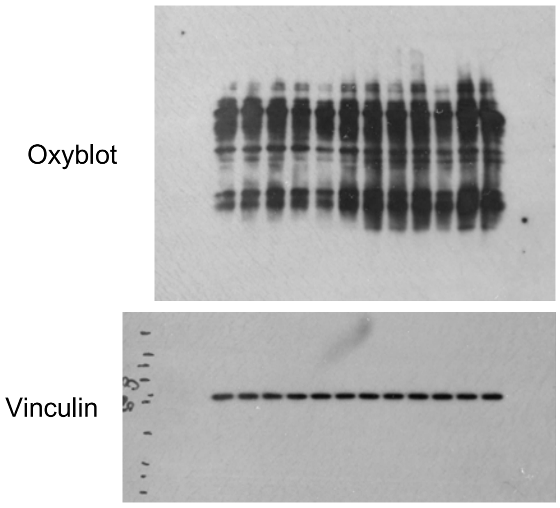

Full unedited gel for Figure S4G

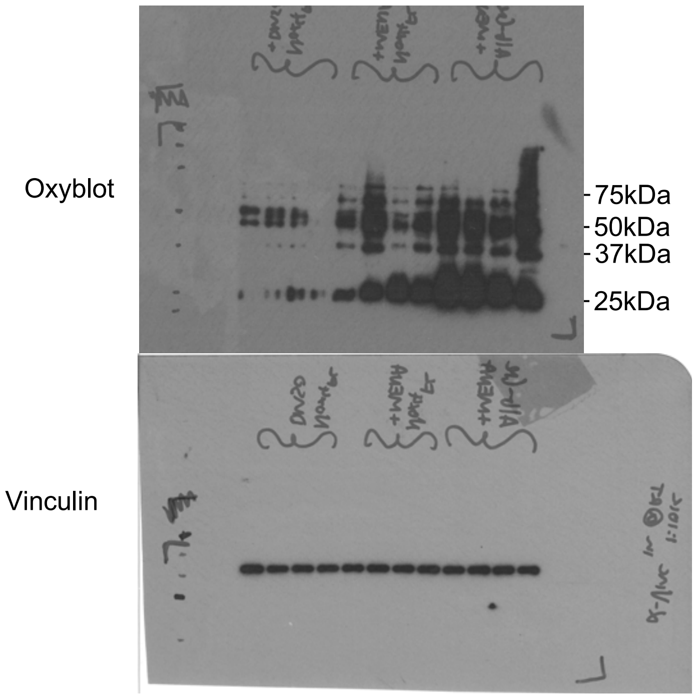

Full unedited gel for Figure S4H

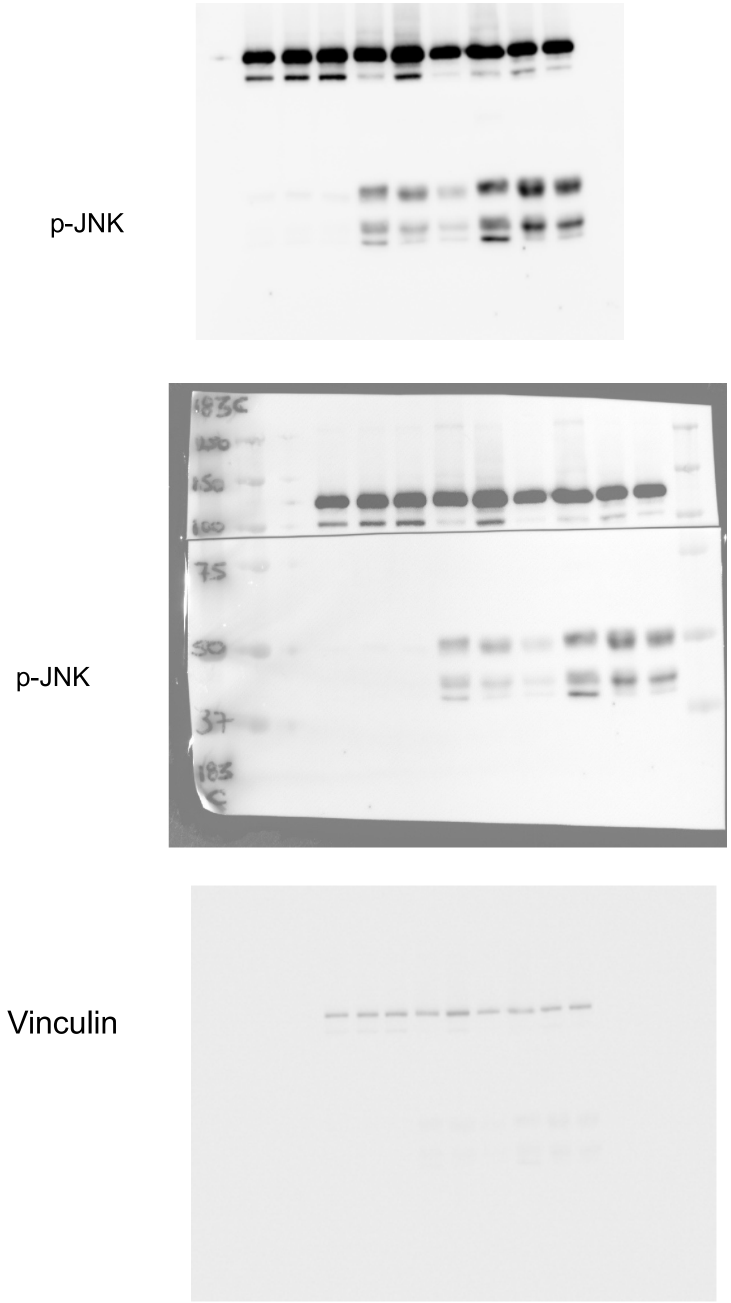

Full unedited gel for Figure S4I

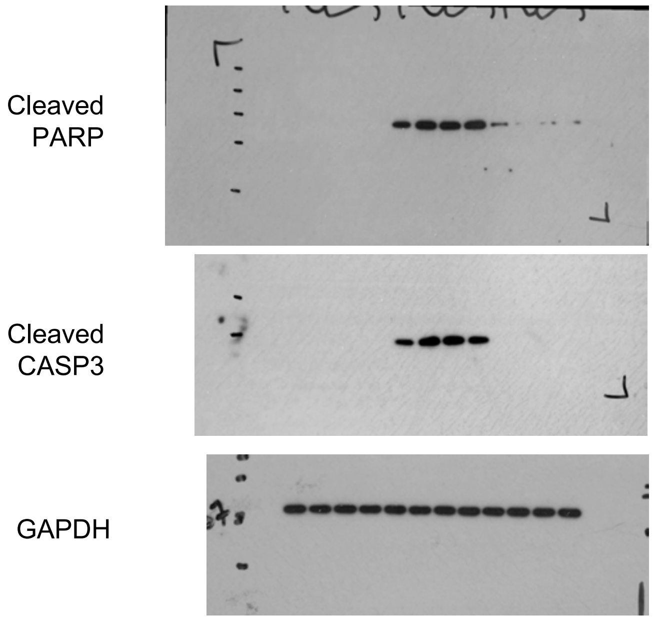

Full unedited gel for Figure S5B

NOX4

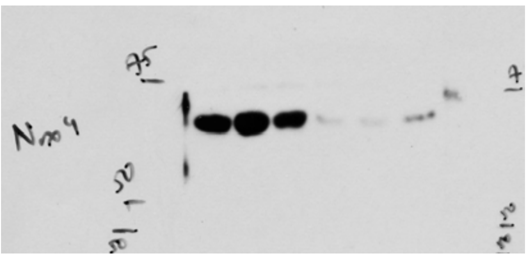

Actin

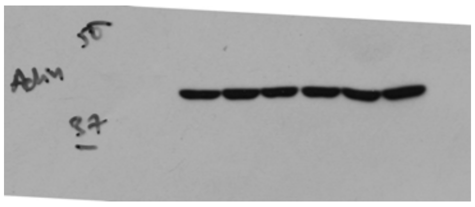

Supplement: Unedited blot and gel images [file jci-134-162533-s018.pdf]
